# Protocatechuic acid prevents obesity caused by long-chain saturated fatty acid-induced inflammation in mouse microglia via inhibition of the NF-κB pathway

**DOI:** 10.1371/journal.pone.0347055

**Published:** 2026-06-01

**Authors:** Ken-yu Hironao, Shoma Fujino, Yusei Johta, Kevin Odongo, Hitoshi Ashida, Yoko Yamashita

**Affiliations:** Department of Agrobioscience, Graduate School of Agricultural Science, Kobe University, Nada-ku, Kobe‌‌, Japan; Emory University School of Medicine, UNITED STATES OF AMERICA

## Abstract

Long-chain saturated fatty acids (LCSFAs), abundant in animal fats, can directly activate microglia and elicit inflammatory responses. Excessive intake of LCSFA-rich high-fat diets (HFDs) has been linked to microglial activation in the brain (particularly within the hypothalamus, a central regulator of energy metabolism) and to metabolic disorders, including obesity. Here, we report that protocatechuic acid (PCA) suppressed the inflammatory response in murine microglia induced by LCSFAs. PCA inhibited the ubiquitin-proteasome degradation of IκBα induced by LCSFAs, suppressing the nuclear translocation of NF-κB and the expression of pro-inflammatory cytokine genes, which was indicated to be attributed to the suppression of I kappa B kinase. In addition, PCA prevented obesity by inhibiting the accumulation of activated microglia in the ARC of HFD-fed mice. This study is the first to demonstrate that PCA suppresses the inflammatory response of microglia induced by LCSFAs and ARC inflammation in HFD-fed mice. These findings provide new evidence and insights into the mechanisms by which polyphenols, including PCA and its analogs, ameliorate diet-induced obesity.

## 1. Introduction

Overconsumption of a high-fat diet (HFD) in mice can lead to obesity via metabolic dysfunction of peripheral tissues and desynchronization of feeding rhythms from the sleep-wake cycle [1–3]. The arcuate nucleus (ARC) of the hypothalamus is the primary central regulator monitoring energy status to control metabolism and feeding behavior. Numerous studies reported that HFD-induced obesity is attributed to inflammation in the ARC [4–7]. Hypothalamic inflammation arises from the accumulation of pro-inflammatory microglia, brain macrophage-like cells. Microglial accumulation in the ARC is induced within a few days of HFD feeding, leading to excessive release of pro-inflammatory factors and neurotransmitters [5]. These factors further recruit surrounding microglia and circulating myeloid cells to the ARC, which interfere with neuronal function [5–7]. Some reports indicate that obesity induced by a HFD can be mitigated through pharmacological suppression of microglial activation, removal of microglia through radiation exposure, and a combination of genetic modification and tamoxifen administration to suppress the microglial activity [3,4,6,7]. Therefore, inhibition of microglial accumulation in the ARC can be expected to be beneficial in treatment of HFD-induced obesity.

Long-chain saturated fatty acids (LCSFAs, such as palmitic and stearic acid), major components of animal fats, contribute to microglial activation [7]. Ingested LCSFAs reach the mediobasal hypothalamus within a few hours via the circulation as part of triglycerides packaged into very low-density lipoprotein particles, and selectively accumulate within the microglia in the ARC [7]; levels of palmitic acid, a constituent of phospholipids and sphingolipids in the hypothalamus, particularly increase [7]. The toll-like receptor 4 (TLR4) pathway [8,9] is the principal pathway mediating LCSFA-induced inflammatory cytokine expression in microglia. TLR4 is a well-known receptor with ligands including endotoxins like lipopolysaccharide (LPS), which rapidly activate downstream inflammatory response signals. The inflammation triggered by palmitic acid is mainly induced through the activation of JNK and NF-κB pathways. In addition to NF-kB, inflammation is induced via various signaling pathways mediated by TLR4. Apart from the TLR4 pathway, LCSFAs can also induce inflammation through their metabolic conversion to sphingolipids. Sphingolipids contribute to endoplasmic reticulum stress and cause mitochondrial damage leading to reactive oxygen species (ROS) production [8,10,11]; both endoplasmic reticulum stress and ROS lead to pro-inflammatory gene expression. Compounds or factors capable of inhibiting these inflammatory signaling pathways could therefore offer an approach to prevent LCSFA-induced inflammation in microglial cells.

Several polyphenols have been reported to contribute to the prevention of HFD-induced obesity by reducing inflammation and accumulation of microglia in the ARC. For example, chronic (40 days) administration of Kaempferol (0.5 mg/kg/day, intraperitoneal) to obese adult mice resulted in improvement of obesity with suppression of microglial activation in the ARC [12]. Dietary supplementation of (-)-Epigallocatechin Gallate significantly inhibited HFD-induced obesity by enhancing brown adipose tissue thermogenesis, and attenuated the hypothalamic inflammation and microglia overactivation [13]. Currently, only these two polyphenols have been reported, and the detailed mechanisms of both are not sufficiently clear. In our previous research, cyanidin 3-*O*-glucoside (C3G), a type of anthocyanin, suppressed the accumulation of microglia in the ARC of HFD-fed mice, correcting disrupted feeding rhythms and improving obesity [14]. It was well known that C3G has low stability in the body and is decomposed and metabolized into various structures: orally administered C3G is quickly metabolized to cyanidin, phenolic acids, and aromatic aldehyde, and only ~12.4% is absorbed intact [15–17]. Nevertheless, in rats fed with C3G-enriched diets, C3G and its metabolites are detected in the brain and various peripheral organs [18,19]. In addition, these compounds are suggested to cross the blood-brain barrier [15]. However, it is unclear whether C3G or its metabolites could directly suppress microglial activation induced by fatty acids contained in HFD. Therefore, the objective of this study was to explore compounds, such as C3G and its major metabolites can prevent obesity by suppressing the inflammatory response induced by LCSFAs in microglial cells, and to elucidate their molecular mechanisms of action.

## 2. Materials and methods

### 2.1. Chemicals and reagents

Penicillin G sodium salt was purchased from Sigma-Aldrich Inc. (St Louis, MO, USA). Streptomycin Sulfate was purchased from MP Biomedicals (Santa Ana, CA, USA). Fetal bovine serum (FBS) was purchased from BioWest S.A.S. (Nuaillé, France). Fatty acid-free bovine serum albumin pH 7.0 (for cell culture) (BSA) was purchased from Nacalai Tesque, Inc. (Kyoto, Japan). Dulbecco’s modified Eagle’s medium (DMEM) was purchased from Nissui Pharmaceutical (Tokyo, Japan). Fatty acid-free BSA pH 7.0 (for immunostaining), 2-mercaptoethanol, insulin, palmitic acid, stearic acid, oleic acid, linoleic acid and α-linolenic acid were purchased from Fujifilm Wako Pure Chemical Co., Ltd. (Osaka, Japan). Cyanidin 3-*O*-glucoside and cyanidin chloride were purchased from Nagara Science Co., Ltd. (Gifu, Japan). Pentobarbital sodium salt, *trans*-ferulic acid, 4-hydroxybenzaldehyde, phloroglucinaldehyde, protocatechuic acid (PCA), vanillic acidand isovanillic acid were purchased from Tokyo Chemical Industry Co., Ltd. (Tokyo, Japan). 2’,7’-Dichlorofluorescein diacetate (DCFH_2_-DA) was purchased from Cayman Chemical (Ann Arbor, MN, USA). Diets for animal experiments, a standard diet (SD) [#D12450J; 3.85 kcal/g; 10.0% kcal from fat (4.4% lard and 5.6% soybean oil), 20.0% kcal from protein, and 70.0% kcal from carbohydrates] and a HFD [#D12492; 5.24 kcal/g; 60.0% kcal from fat (54.4% lard, 5.6% soybean oil), 20.0% kcal from protein, and 20.0% kcal from carbohydrates] were purchased from Research Diets, Inc. (New Brunswick, NJ, USA). Domitor^®^ (1.0 mg/mL medetomidine hydrochloride) was purchased from Nippon Zenyaku Kogyo Co., Ltd. (Fukushima, Japan). Synthesized DNA and RNA oligos were purchased from Eurofins Genomics Co., Ltd. (Tokyo, Japan). Primary antibodies for western blotting [rabbit polyclonal antibodies of anti-β-actin, anti-GAPDH, anti- NF-κB p65, anti-IκBα, anti-Erk1/2, anti-phospho-Erk1/2 (Thr202/Tyr204), anti-JNK, anti-phospho-JNK (Thr183/Tyr185), anti-Lamin B1, and anti-ubiquitin; rabbit monoclonal antibody of anti-phospho-NF-κB p65 (Ser536) (93H1); and mouse monoclonal antibody of anti-phospho-IκBα (Ser32/36) (5A5)], and secondary HRP-linked antibodies (goat anti-rabbit IgG and horse anti-mouse IgG) were purchased from Cell Signaling Technology Co., Ltd. (Danvers, MA, USA). The antibody lists for Western blotting, Immunocytochemistry and Immunohistchemistry are provided in the Supporting Information as Supplemental Tables (S1-S3 Tables). For all reagents, those of sufficient grade for each experiment were selected.

### 2.2. Cell culture

MG6 cells [20,21] (RIKEN Cell Bank, Ibaraki, Japan), a c-myc-immortalized mouse microglia cell line, were maintained in DMEM containing 10% (v/v) FBS supplemented with 0.1% (w/v) NaHCO_3_, 25 mM D-glucose, 4 mM L-glutamine, 100 U/mL penicillin, 100 µg/mL streptomycin, 100 µM 2-mercaptoethanol and 10 µg/mL insulin under humidified atmosphere of 95% (v/v) air and 5% (v/v) CO_2_ at 37°C. Cells were pre-cultured to 90% confluence, then the maintenance medium was replaced with medium containing 0.45% (w/v) fatty acid-free BSA for 16 h before the experiments. To knockdown JNK and NF-KB expression, the following small interfering RNAs (siRNAs) were used; 5’-UCU AUU ACU AGC AUU UUG GAU-3’ for JNK, 5’-AGA UAA AGA GUA AAA CGU GCC-3’ for NF-κB. MISSION^®^ siRNA Universal Negative Control #1 (Sigma-Aldrich) was used for the control. Those siRNAs were transfected into the cells using Lipofectamine^TM^ RNAiMAX Transfection Reagent (Thermo Fisher Scientific Co., Ltd., Waltham, MA, USA) following manufacturer’s instruction.

### 2.3. Preparation of the fatty acid–BSA complex

The fatty acid–BSA complex was prepared according to a previous report with minor modifications [22]. First, to make fatty acid sodium solutions, 100–300 mM DMSO stock solutions of fatty acids and 0.01 N NaOH were mixed at a 1:9 ratio, then incubated at 75°C for 30 min with occasional mixing. Dissolution of some fatty acids was accelerated by dropwise addition of 1 N NaOH. The fatty acid sodium solutions were complexed with medium containing 5% (w/v) BSA at a 1:9 ratio, then incubated at 37°C for 30 min with constant mixing. The complexed fatty acid–BSA medium was added to a serum-free medium to achieve a fatty acid concentration of 100–300 μM, BSA concentrarion of 0.5% (w/v), and a DMSO concentration of 0.1% (v/v).

### 2.4. Determination of the scavenging capacity for ROS under cell-free conditions

The scavenging capacity for ROS of test compounds was determined by measuring oxygen radical absorbing capacity using 2,2′-azobis (2-amidinopropane) dihydrochloride (AAPH) as previously reported [23]. Fluorescence was recorded every 2 min for 180 min with an excitation and emission wavelength of 485 nm and 535 nm, respectively, using a Wallac 1420 ARVOsx Multilabel Counter (Perkin-Elmer, Boston, MA, USA). ROS scavenging activity was evaluated by observing the decrease in fluorescence.

### 2.5. Determination of intracellular ROS

Evaluation of the scavenging capacity of test compounds for ROS in MG6 cells was performed as previously reported with minor modifications [24]. To determine intracellular ROS in MG6 cells, DCFH_2_-DA was introduced. MG6 cells cultured in a 96-well plate were treated with medium containing 100–300 µM fatty acid–BSA complex or 100 µM AAPH, and treated with each of the test compounds for 24 h; they were then washed with PBS and treated with 10 µM DCFH_2_-DA-containing serum-free medium for 1 h. After that, cells were washed twice with PBS and collected in RIPA buffer. Intracellular ROS levels were quantified by measuring the fluorescence of DCF, a fluorescence indicator converted from DCFH-scavenging radicals, at excitation and emission wavelengths of 485 nm and 535 nm, respectively, by an ARVO X4 plate reader.

### 2.6. Measurement of cytokine release into the culture medium

Quantification of the amount of TNF-alpha released into the culture medium was conducted using a commercial kit: Mouse TNF-alpha DuoSet ELISA (R&D Systems, Inc., Minneapolis, MN, USA). Briefly, MG6 cells cultured in a 96-well plate were treated with medium containing fatty acid–BSA complexes or test compounds for 24 h, then the culture medium was collected into 1.5 mL microtubes and centrifuged at 12,000 × g for 10 min at 4°C. The supernatant was transferred into another tube and stored at −80°C until analysis without a freeze-thaw cycle.

### 2.7. RNA isolation and real-time quantitative PCR (RT-qPCR) analysis

Total RNA was isolated from MG6 cells using TRIzol™ Reagent (Invitrogen, Carlsbad, CA, USA), following the manufacturer’s guidelines. Total RNA was qualified and quantified with a NanoDrop™ ND-1,000 spectrophotometer (Thermo Fisher Scientific), then reverse-transcribed to cDNA with ReverTra Ace^®^ (Toyobo Co., Ltd.), and subjected to RT-qPCR amplification using TB Green^®^ Premix Ex Taq™ II (Takara Bio, Kusatsu, Japan) in a TaKaRa PCR Thermal Cycler Dice^®^ Real Time System II (Takara Bio). Relative gene expression was determined using the comparative CT method, with *Gapdh* as the reference gene, then presented as fold differences relative to the control group. The primer sequences used were as follows: *Gapdh* (forward, 5′-CAT GGC CTT CCG TGT TCC TA-3′ and reverse, 5′-CCT GCT TCA CCA CCT TCT TGA-3′), *Tnf-alpha* (forward, 5′-GAC AGT GAC CTG GAC TGT GG-3′ and reverse, 5′-TGA GAC AGA GGC AAC CTG AC-3′), *Il1b* (forward, 5′-GTT GAC GGA CCC CAA AAG ATG-3′ and reverse, 5′-GCT GCT GCG AGA TTT GAA GC-3′), *Il6* (forward, 5′-AGT CCG GAG AGG AGA CTT CA-3′ and reverse, 5′-ATT TCC ACG ATT TCC CAG AG-3′), *Ccl2* (forward, 5′-GAA GGA ATG GGT CCA GAC AT-3′ and reverse, 5′-ACG GGT CAA CTT CAC ATT CA-3′).

### 2.8. Predaration of whole cell lysates and nuclear fractions, and western blotting analysis

Whole-cell lysates and nuclear fractions were prepared as described in previous reports [25]. Western blotting and densitometric quantification of protein-specific bands were also performed as previously reported [14]. The antibodies used for detecting proteins are listed in Section 2.1. The primary antibody was reacted at a 5000:1 dilution, and the secondary antibody was reacted at a 20,000:1 dilution, respectively.

### 2.9. Immunocytochemistry

MG6 cells were cultured on 12-mm-diameter coverslips coated with Cellmatrix Type I-C (Nitta Gelatin Co., Ltd., Osaka, Japan) in a 24-well culture plate, and immunofluorescence staining was performed as previously described [25]. The cells were immunostained with anti-NF-κB p65 (D14E12) rabbit monoclonal antibody (1:400; Cell Signaling Technology) overnight at 4°C, followed by incubation with Alexa Fluor™ 488-conjugated goat anti-rabbit IgG (1:1,000; Invitrogen). The nuclei were counterstained with 1 μg/mL 4’,6-diamidino-2-phenylindole. Images were acquired using a fluorescence microscope (#FSX100; Olympus, Tokyo, Japan).

### 2.10. Immunoprecipitation

For the preparation of carriers used for immunoprecipitation, anti-IκBα antibody and rabbit IgG (for control) were conjugated to Dynabeads M270-Amine (Veritas Co., Ltd., Tokyo, Japan) by hydrolysis reaction with an N-hydroxysuccinimide ester, following the manufacturer’s instructions. The MG6 cell pellet was suspended with Pierce™ IP Lysis Buffer (Thermo Fisher Scientific) containing 1 mM dithiothreitol and protease/phosphatase inhibitors [30 KIU/mL aprotinin, 5 μg/mL leupeptin, 1 mM phenylmethylsulfonyl fluoride, 10 mM sodium fluoride and 1 mM sodium orthovanadate (V)], then incubated on ice for 15 min. The suspension was centrifuged at 12,000 × g for 15 min at 4°C, then the supernatant was diluted with PBS containing 0.05% (v/v) tween-20 (PBST) to 2 mg/mL protein concentration (the Input). Three milligrams of Dynabeads M270-Amine conjugated with anti-IκBα antibody or rabbit IgG was added to 500 µL of the Input, then rotated for 2 h at 4°C. The beads were washed with immunoprecipitation lysis buffer, then the target protein was eluted with 100 mM citrate buffer (pH 3.0). The elution was subjected to western blotting analysis after neutralization with 1 M Tris-HCl (pH 8.0).

### 2.11. Pull down assay

The nuclear protein extraction from MG6 cells was performed according to a previous report [26]. One nmol of 5′-biotin-labeled double-stranded DNA probe specific for NF-κB responsive element (5′-AGTTGAGGGGACTTTCCCAGGC-3′; biotin-labeled NF-κB probe), was mixed with 1 mg of streptavidin-conjugated magnetic beads (FG beads, Tamagawa Seiki Co., Ltd., Nagano, Japan) in binding buffer [5 mM Tris-HCl (pH 7.5), 0.5 mM EDTA-2Na and 1 mM NaCl], then rotated for 1 h at 4°C. For the competition assay, a 200-fold molar excess of unlabeled probe (cold probe) was added. The FG beads were reacted with 0.1 mg of isolated nucleoproteins in rotattion for 1 h at 4°C. After washing the FG beads with PBST, SDS buffer [62.5 mM Tris-HCl (pH 6.5), 2% (w/v) SDS, 10% (v/v) glycerol, 5% (v/v) 2-mercaptoethanol, and 0.02% (w/v) bromophenol blue] was added, then boiled for 5 min. The sample was centrifuged at 20,000 × g for 5 min at 4°C, then the supernatant was subjected to Western blotting.

### 2.12. Reporter assay

Reporter assays were performed as previously described [27]. *Photinus pyralis* luciferase reporter vector pGL4.20 (Promega) inserted with mouse *Il6* promoter together with a TATA box sequence (5′- TCG AGG TGG GAT TTT CCC ATG AGT CTC AAA ATT AGA GAG TTG ACT CCT AAT AAA TAT TGA −3′), and *Renilla* luciferase reporter vector pRL-SV40 (Promega) were transiently transfected into MG6 cells using Lipofectamine^TM^ 2000 reagent (Thermo Fisher Scientific). MG6 cells were treated with PCA for 1 h, followed by treatment with palmitic acid for 24 h. Luciferase activities were measured using the PicaGene Dual Sea Pansy Luminescence Kit (TOYO INK, Tokyo, Japan) and an ARVO X4 plate reader. Data were expressed as relative light units (RLU; *Photinus pyralis* luciferase activity/*Renilla* luciferase activity).

### 2.13. *In vitro* IKK kinase assay

The evaluation of the inhibitory effect of PCA on IKK kinase activity was measured by IKKβ Kinase Enzyme System with ADP-Glo™ Assay (Promega, Madison, WI, USA). Briefly, the indicated concentration of the IKK inhibitor (TPCA-1) and PCA were mixed with ATP solution, followed by the addition of IKKβ recombinant protein, which converted ATP to ADP. After the removal of unreacted ATP, an enzyme (ADP-GroTM) was added to catalyze the oxidation of luciferin corresponding to the quantity of ADP. The oxidized luciferin was quantified by using an ARVO X4 plate reader and expressed as relative light units (RLU).

### 2.14. Animal experiments

Approval of all animal experiments was granted by the Institutional Animal Care and Use Committee (approval number: #2020-10-13) and the procedures adhered to the Guidelines for Animal Experiments by Kobe University. C57BL/6J mice (male, 5 weeks old; Japan SLC, Inc., Shizuoka, Japan) were housed individually in a climate-regulated chamber (temperature: 23 ± 2°C, humidity: 50 ± 10%) following a 12:12-h light-dark cycle. The mice underwent acclimatization in their surroundings for one week, with free access to tap water and SD. Thirty mice were allocated to six groups of five each, and fed a SD or HFD alone, or supplemented with 0.5% (w/w) PCA. Powdered diets (5.0 g/day) were dispensed into a feeder and refreshed every 2 or 3 days. Food intake was calculated from the food remaining at the time of each replacement. After the experiment, mice were perfused with 4% paraformaldehyde in PBS under anesthesia with intraperitoneal injection of a combination of sodium pentobarbital (65 mg/kg) as an anesthetic and medetomidine hydrochloride (0.3 mg/kg) as an analgesic to collect brain samples. To obtain the hypothalamus, the collected brain was cut out with a range of bregma –1.00 to –2.50 using a rodent brain matrix, then pre-fixed with 4% paraformaldehyde in PBS followed by standard dehydration procedure and embedded in paraffin.

### 2.15. Immunohistochemistry and activated microglia counting

The coronal brain paraffin blocks were sliced into 10 µm-thick sections and subjected to immunostaining as previously reported [14]. The nuclei were stained with 1 µg/mL 4’,6-diamidino-2-phenylindole in PBS. For the detection of Iba1, a mouse monoclonal [EPR16589] primary antibody against Iba1 (1:200; Abcam) and an Alexa Fluor™ 594-conjugated goat anti-mouse IgG (1:1,000; Invitrogen) were used. For detection of cluster of differentiation 68 (CD68), a rabbit monoclonal primary antibody (E3O7V) against CD68 (1:1,000; Cell Signaling Technology) and an Alexa Fluor™ 488-conjugated goat anti-rabbit IgG (1:1,000) were used. The number of Iba1-immunoreactive cells in the ARC of the matched sections was counted manually using ImageJ software. For counting the number of CD68-overwrapped Iba1-immunoreactive cells, CD68-immunoreactive particles with an area of 5–10 μm^2^ were considered positive, and counts were manually obtained in a blinded fashion from three ARC sections each per mouse as previously described [28]. The threshold was defined as the intensity where cells were clearly CD68-positive by visual inspection. The counting data are presented as averages per mouse.

### 2.16. Content of PCA

PCA content in the brain was measured by a high-performance liquid chromatography (HPLC) method. The quantity of PCA uptake into MG6 cells was also measured. Briefly, the hypothalamus was homogenized with PBS. In the case of MG6 cells, the cells were treated with PCA at 10 μM for 24 h and homogenized in a 1% triton-X 100 solution. Obtained homogenate from the hypothalamus or MG6 cells was transferred to perfluoroalkoxy alkanes (PFA) tubes (15 ml, Savillex, Eden Prairie, MN, USA) and acidified by adding 2% (w/v) ascorbic acid. Then, quercetin was added to the homogenate as an internal standard. To remove lipids, 1 ml hexane was added to the mixture, vigorously mixed for 30 s and centrifuged at 3000 × g for 10 min. After removal of the hexane layer, 1 ml acetonitrile was added to precipitate the protein and the mixture was again vigorously mixed and centrifuged under the same conditions. Supernatants were collected and transferred to a new PFA tube and PCA was extracted with 1 ml ethyl acetate. After vigorous mixing, the ethyl acetate layer was collected by centrifugation. This extraction step was repeated two more times. The obtained ethyl acetate extract was evaporated to dryness. The dried material was dissolved in 50 μL of 50% methanol and applied to HPLC. HPLC was performed using a system equipped with a DGU-20A 3R degassing unit, LC-20AD XR binary pump, SIL-20 AC XR autosampler, SPD-M20A diode array detector, CTO-20 AC column oven and CBM-20A communications bus module connected to an LC work station (Shimadzu Corporation, Kyoto, Japan). The analytical column was a Cadenza CL-C18 column (φ 250 mm × 4.6 mm, 3 μm, Imtakt, Kyoto, Japan), protected by a guard column (Cadenza CL-C18, φ 5 mm × 2 mm, 3 μm, Imtak, Kyoto, Japan). Briefly, 1% (v/v) formic acid was mobile phase A and acetonitrile was mobile phase B. Separation of PCA was achieved using these linear gradients: 5%–80% B over 0–30 min; 80%–90% B over 30–40 min. The flow rate was 1.0 ml/min, the injection volume was 10 μL and the temperature of the column oven was 40 °C. The limit of detection and quantification was 0.02 and 0.09 ng/ml, respectively.

### 2.17. Statistical analysis

After confirming the normality of all datasets using an F-test, statistical differences were investigated with a two-tailed t-test, Dunnett’s test, or the Tukey–Kramer honestly significant difference test using JMP statistical software version 11.2.0. (SAS Institute); a *p*-value < 0.05 was considered statistically significant. Data are expressed as the mean and standard deviation or standard error. The statistical analysis employed for each dataset is outlined in figure captions.

## 3. Results

### 3.1. Suppression of free fatty acid-induced inflammatory responses in murine microglia by C3G and its metabolites

**Fig 1:**
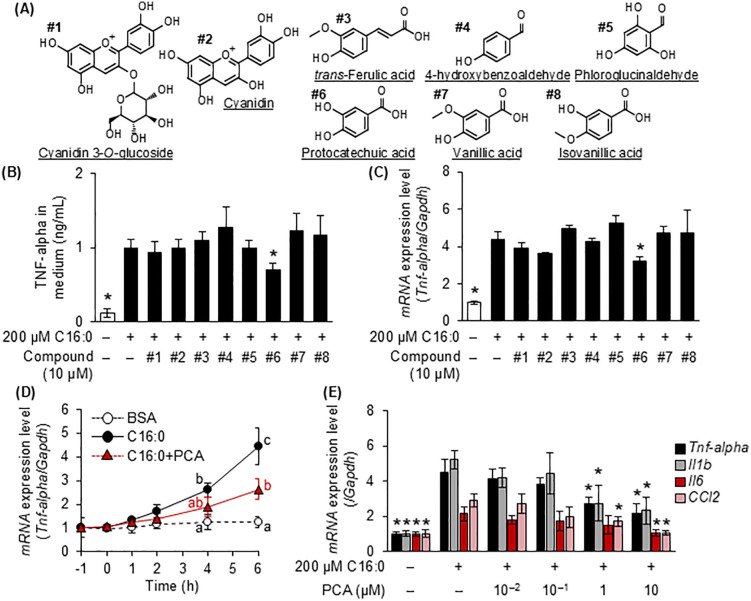
Screening test for cyanidin 3-*O*-glucoside and its metabolites which suppresses fatty acid-induced inflammatory responses in murine microglia MG6 cells. **(A)** Structure of C3G and its major metabolites used in this screening test. Compound numbers correspond to those shown in each experiment. **(B)** The amount of TNF-alpha released into the medium from MG6 cells pre-treated with C3G and its metabolites for 1 h and‌‌ then with 200 µM palmitic acid for 24 **h. (C)** After treatment with C3G and its metabolites for 1 h, *Tnf-alpha* expression level in MG6 cells treated with 200 µM palmitic acid for 6 h was measured by quantitative RT-PCR. Each value was normalized by the gene expression level of the BSA group. **(D)** After treatment with 10 µM protocatechuic acid (PCA) for 1 h, 200 µM palmitic acid was treated, and the expression level of *Tnf-alpha* in MG6 cells was measured by quantitative RT-PCR at described time points. Each value was normalized to baseline (−1 **h)**. **(E)** Gene expression levels of inflammatory markers in MG6 cells treated with PCA at each listed concentration for 1 h and then 200 µM palmitic acid for 6 h were measured by quantitative RT-PCR. Each value was normalized to BSA group. Data shown are mean ± standard deviation (*n* = 3). (B, C, and **E)** The asterisks represent significant differences vs. palmitic acid group, and (D) the different letters represent significant differences among the three groups at each time point by Tukey-Kramer honestly significant difference test (*p* < 0.05).

First, we investigated the effects of the five major fatty acids constituting lard [palmitic acid (C16:0), stearic acid (C18:0), oleic acid (C18:1), linoleic acid (C18:2), and linolenic acid (18:3)] on inflammatory responses in MG6 cells. The LCSFAs (palmitic acid and stearic acid) strongly stimulated TNF-alpha release in MG6 cells in a concentration-dependent manner, whereas unsaturated fatty acids (oleic acid, linoleic acid, and linolenic acid) did not (S1A Fig). After 6 h, treatment with LCSFAs resulted in significantly higher gene expression of pro-inflammatory cytokines (*Tnf-alpha*, *Il1b*, *Il6*) and a chemokine (*Ccl2*) compared with baseline (0 h), whereas unsaturated fatty acids did not (S1B Fig). Because palmitic acid especially accumulates in microglia in the ARC [6], we used palmitic acid as an LCSFA in the following experiments. Palmitic acid-induced TNF-alpha release and *Tnf-alpha* expression were suppressed by PCA, the #6 compound described in Fig 1A (Figs 1B and 1C), an effect that was significant 6 h after treatment (Fig 1D). PCA also suppressed the other inflammation-related genes induced by palmitic acid in a concentration-dependent manner (Fig 1E). The BSA treatment for 6 h did not produce any detectable changes in the expression of inflammation‑related genes relative to the baseline (0 h) (Fig 1E and S1B). Under the experimental conditions employed, the potential pro‑inflammatory effects of BSA described in previous studies can therefore be regarded as negligible [29,30]. These results suggest that among C3G metabolites, PCA is a candidate compound that suppresses LCSFA-induced microglial inflammation.

### 3.2. The radical-scavenging ability of C3G and its metabolites in response to palmitic acid-induced inflammation in murine microglia

**Fig 2:**
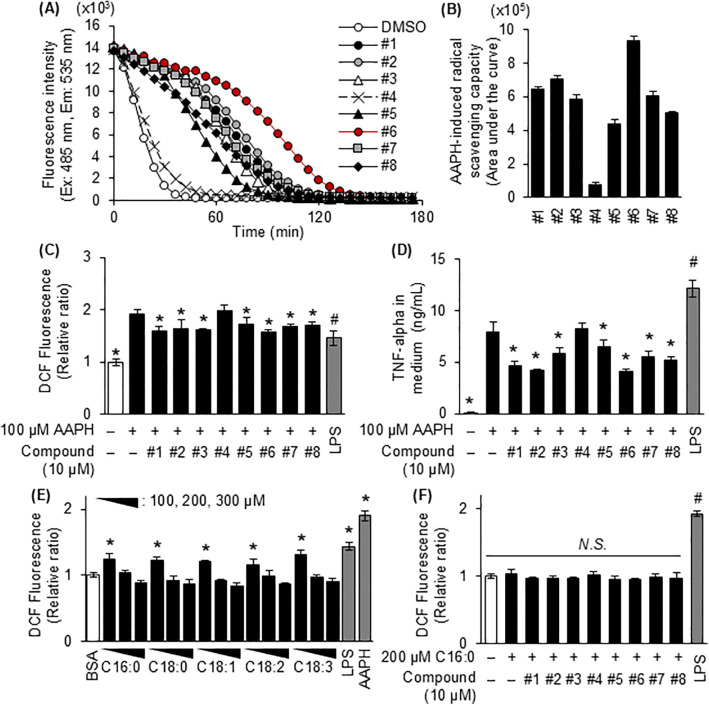
The relationship between radical scavenging activity and the anti-inflammatory effect of C3G and its metabolites. **(A)** Fluorescein fluorescence decay curve and (B) radical scavenging capacity of C3G and its metabolites. Fluorescence intensity was detected and analyzed. Radical scavenging capacity was calculated by subtracting the value for the DMSO group from the area under the fluorescence decay curve for each compound. **(C)** MG6 cells were treated with 100 µM 2’,7’-dichlorodihydrofluorescein diacetate (DCFH_2_-DA) for 30 min followed by treatment with compounds described in Fig 1A at a concentration of 10 µM for 1 **h.** Then, 30 min after the addition of 100 µM AAPH, the fluorescence intensity (Ex: 485 nm, Em: 535 nm) of dichlorofluorescein (DCF) generated from oxidized dichlorofluorescein (DCFH) in the cells was measured. Each value was normalized to control group. **(D)** The amount of TNF-alpha released into the medium was measured by ELISA after treatment of MG6 cells with C3G or its metabolites at a concentration of 10 µM for 1 h followed by 100 µM AAPH for 24 **h. (E)** Intracellular reactive oxygen species were measured after treatment of MG6 with 100-300 µM palmitic acid (C16:0), stearic acid (C18:0), oleic acid (C18:1), linoleic acid (C18:2) and α-linolenic acid (C18:3) for 24 **h.** Each value was normalized to BSA group. **(F)** The amount of ROS in MG6 cells pretreated with C3G or its metabolites described in Fig 1C for 60 min and treated with 200 µM palmitic acid for 24 h was measured as described in materials and methods section. Each value was normalized to BSA group. Data shown are mean ± standard deviation (*n* = 3). (C, D, and **F)** The asterisks represent significant differences vs. the palmitic acid group by Tukey-Kramer honestly significant difference test (*p* < 0.05), and the group with hashtags was excluded from the statistical analysis. **(E)** The asterisks represent significant differences vs. the BSA group by Dunnett’s test (*p < 0.05).

C3G and its metabolites have strong antioxidant activity. When compared with the DMSO group, all the compounds listed in Fig 1A (except 4-hydroxybenzaldehyde) delayed the decay of fluorescein induced by peroxyl radical species from AAPH (Fig 2A); PCA had the highest radical-scavenging capacity (Figs 2A and 2B). Next, we investigated the intracellular radical-scavenging activity of these compounds. Intracellular DCFH_2_-DA is metabolized to DCFH, then oxidized by intracellular ROS to fluorescent DCF. Again, other than 4-hydroxybenzaldehyde, all the compounds significantly suppressed the intracellular DCF accumulation induced by AAPH treatment (Fig 2C). These compounds also suppressed AAPH-induced TNF-alpha release in MG6 cells (Fig 2D). Excessive free fatty acids increase intracellular ROS levels in macrophages and induce the inflammatory response [31]. Unexpectedly, within the concentration range of 100–300 µM, each of fatty acids elevated intracellular DCF fluorescence in MG6 cells only at the lower concentration of 100 µM, whereas no increase was observed at 200 or 300 µM. (Fig 2E). In addition, in the MG6 cells pre-treated with 10 µM C3G and its metabolites, treatment with 200 µM palmitic acid did not affect intracellular ROS accumulation (Fig 2F). These results suggest that the inflammatory response induced by 200 μM palmitate in microglial cells occurs via a mechanism independent of ROS generation.

### 3.3. Identification of key pathways for palmitic acid-induced inflammatory responses in MG6 cells and the effect of PCA on these pathways

**Fig 3:**
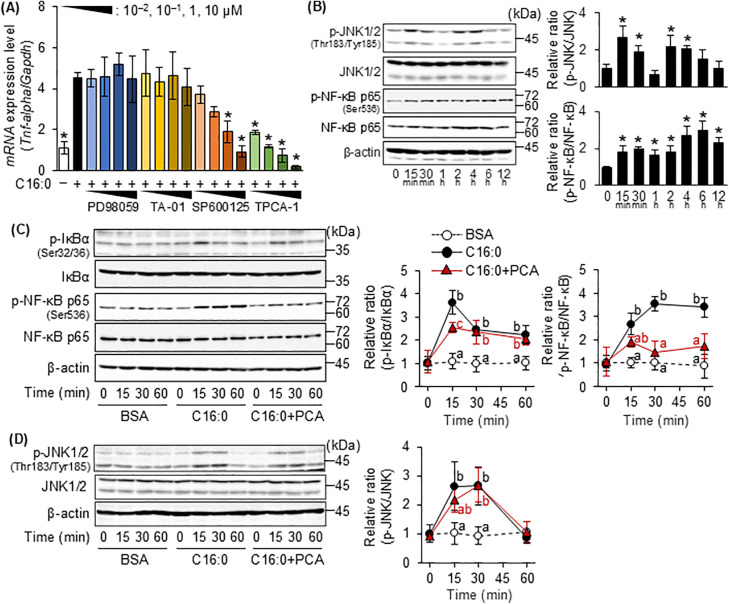
Identification of key pathways through which palmitic acid induces inflammatory responses in MG6 cells and the effect of PCA on these pathways. **(A)** *Tnf-alpha* gene expression level in MG6 was analyzed by quantitative RT-PCR after the treatment with PD98059 (MEK inhibitor), TA-01 (p38MAPK inhibitor), SP600125 (JNK inhibitor), and TPCA-01 (IKK inhibitor) at the listed concentrations for 1 h followed by 200 µM palmitic acid for 6 **h.** Each value was normalized to non-treated group. **(B)** The levels of phosphorylated-c-jun N-terminal kinase 1/2 (p-JNK1/2), JNK1/2, phosphorylated-nuclear factor kappa-B p65 subunit (p-NF-κB p65), NF-κB p65, and β-actin in MG6 cells treated with 200 μM palmitic acid for the indicated time were analyzed by Western blotting. **(C and D)** Western blotting analysis of the effects of PCA on the palmitic acid-treated MG6 cells. Expression of (C) phosphorylated-inhibitor of nuclear factor-kappa B alpha (p-IκBα), IκBα, p-NF-κB p65, NF-κB p65, β-actin, (D) p-JNK1/2, JNK1/2, and β-actin in the whole cell lysate. Quantification of the expression levels of proteins in these Western blotting analyses was performed by ImageJ and the relative amount of each protein was obtained by normalizing it to β-actin level and expressing it as a ratio vs. 0 min group. Data shown are mean ± standard deviation (*n* = 3). **(A and B)** The asterisks represent significant differences vs. (A) non-treated group or (B) 0 min group by Dunnett’s test (*p < 0.05). **(C and D)** The different letters represent significant differences among the three groups at each time point by Tukey-Kramer honestly significant difference test (*p* < 0.05).

Various previous studies have reported that LCSFAs mediate TLR4 activity and activate its downstream signaling cascades, such as MAPK (including ERK1/2, p38MAPK, and JNK) and NF-κB pathways, and induce the expression of genes encoding various inflammatory cytokines [32]. PD98059 (an inhibitor of MEK, an upstream kinase of ERK1/2) and TA-01 (a p38MAPK inhibitor) administered at concentrations ranging from 10^−2^ µM to 10 µM failed to inhibit the induction of *Tnf-alpha* mRNA expression by palmitic acid, whereas SP600125 (a JNK inhibitor) at 1 µM and more, and TPCA-01 (an inhibitor of IκB kinase (IKK), an upstream kinase of NF-κB) administered at 10^−2^ µM to 10 µM significantly inhibited palmitic acid-induced *Tnf-alpha* expression in a concentration-dependent manner (Fig 3A). Indeed, palmitic acid induced the phosphorylation of JNK1/2 and NF-κB p65 within 15 min (Fig 3B). IκBα, an upstream regulator of NFκB, was also phosphorylated within 15 min following palmitic acid treatment, and its protein levels subsequently decreased over time (S2 Fig). Pretreatment with PCA resulted in significantly lower palmitic acid-induced phosphorylation of IκBα at 15 min and NF-κB p65 at 15, 30, and 60 min (Fig 3C) compared with palmitic acid alone. In contrast, PCA failed to inhibit JNK phosphorylation (Fig 3D). In addition, silencing NF-κB inhibited *Tnf-alpha* expression induced by palmitate, whereas silencing JNK did not (S3 Fig). These results suggest that PCA suppresses the inflammatory response of MG6 cells by inhibiting palmitic acid-induced activation of the NF-κB pathway.

### 3.4. The effect of PCA on palmitic acid-induced NF-κB nuclear translocation

**Fig 4:**
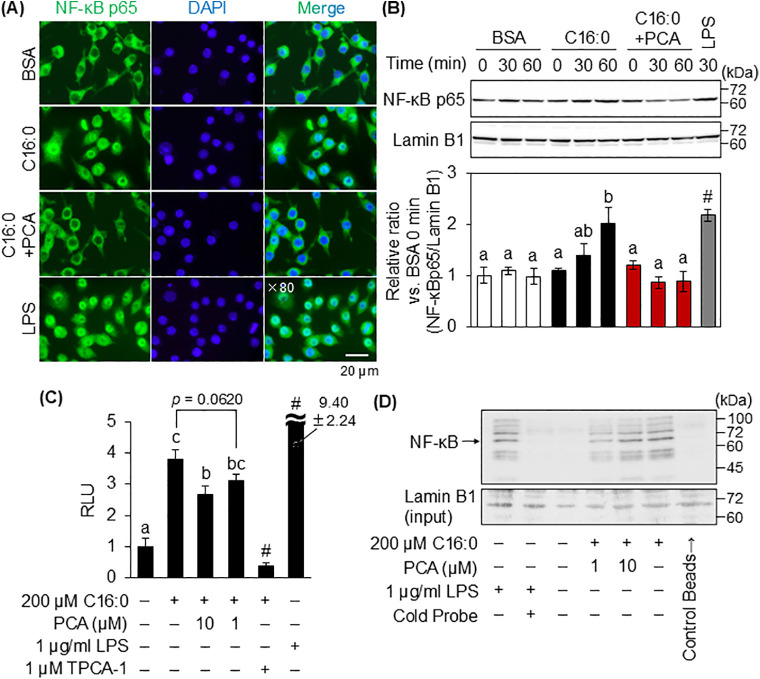
Effect of PCA pretreatment on palmitic acid-induced NF-κB nuclear translocation in MG6 cells. **(A)** MG6 cells pretreated with or without 10 µM PCA for 60 min were treated with 200 µM palmitic acid for 60 min and then fixed with 4% paraformaldehyde. The post-fixed MG6 cells were immunofluorescence-stained for NF-κB p65 with anti-NF-κB p65 antibody (green) and nuclei with 4’,6-diamidino-2-phenylindole (DAPI) (blue). As a positive control for nuclear translocation of NF-κB, MG6 cells were treated with 1 µg/ml LPS for 30 min. **(B)** Western blotting analysis of nuclear proteins isolated from the MG6 cells pretreated with or without 10 µM PCA for 60 min followed by 200 µM palmitic acid for 0, 30, or 60 min. Quantification of the expression levels of NF-κB p65 and Lamin B1 proteins were analyzed by ImageJ software and the relative amount of each protein was obtained by normalizing it to the Lamin B1 level and expressing it as a ratio vs. BSA 0 min group. **(C)** MG6 cells were transiently transfected with reporter vectors pGL4.20-NF-κB-TATA-Luc and pRL-SV40 for 24 h. Then, cells were treated with 1 or 10 µM PCA for 1 h followed by 200 µM palmitic acid for 12 h. TPCA-1 was used as a positive control, and LPS was used as a negative control. **(D)** The nucleoprotein in MG6 cells were reacted with biotin-labeled NF-κB probe, then pull downed with streptavidin-linked magnetic beads (FG beads). The elution was subjected to Western blotting to detect activated NF-κB translocated into the nucleus. Lamin B1 was detected from Input (the extracted nucleoprotein). Control beads mean the Intact FG beads that were not reacted with biotin-labeled NF-κB probe. Data shown are mean ± standard deviation (*n* = 3). Value over the highest value of the graph is noted as actual values on each bar. Different letters represent significant differences by Tukey-Kramer honestly significant difference test (*p* < 0.05), and the group with hashtags was excluded from the statistical analysis.

Activated NF-κB translocates to the nucleus and promotes the expression of pro-inflammatory cytokine-encoding genes [33]. Indeed, 60 min treatment with 200 µM palmitic acid promoted nuclear translocation of NF-κB, whereas pretreatment with PCA inhibited this palmitic acid-mediated effect (Figs 4A and 4B). Next, a plasmid with the NF-κB response element inserted into the promoter region of the firefly luciferase gene was transfected into MG6 cells, and the transcriptional activity of NF-κB was analyzed. Palmitic acid significantly increased the transcriptional activity of NF-κB, while pretreatment with PCA suppressed this palmitic acid-mediated effect (Fig 4C). The results of the pull-down assay showed that the formation of biotin-labeled probes and NF-κB complexes were enhanced in the nucleoproteins of palmitic acid-treated MG6 cells, while pretreatment with PCA suppressed this effect (Fig 4D). These results suggest that PCA inhibits NF-κB nuclear translocation, resulting in suppression of palmitic acid-induced pro-inflammatory cytokine expression through transcriptional activity of the NF-κB responsive element.

### 3.5. The effect of PCA on palmitic acid-induced IκBα degradation

**Fig 5:**
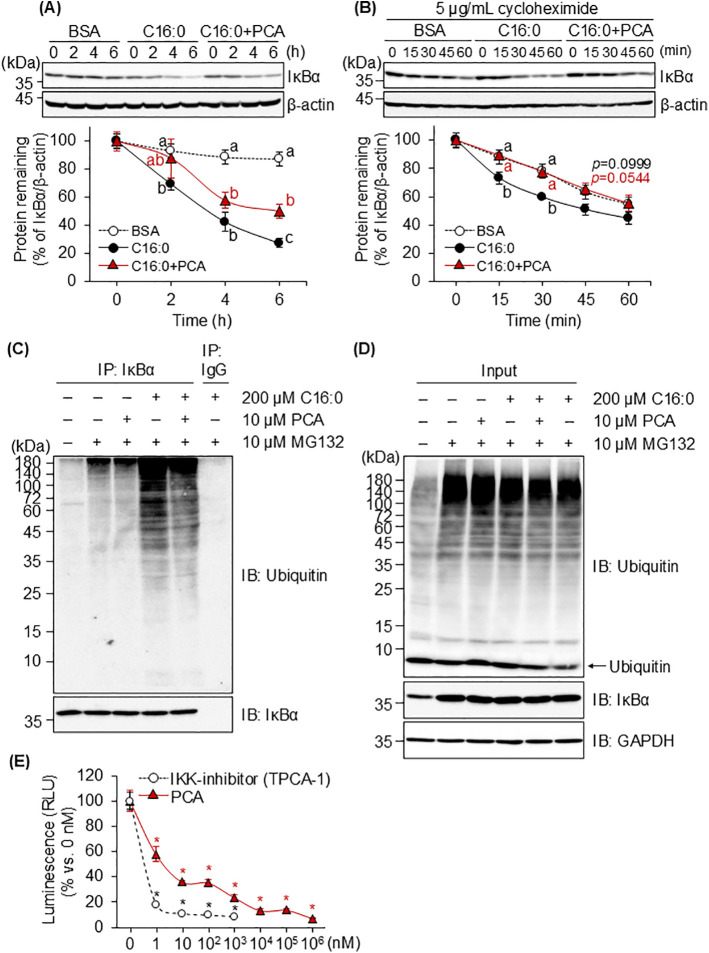
Effect of PCA on IκBα stability in MG6 cells. **(A and B)** Western blotting analysis of whole cell lysate from MG6 cells pretreated with 10 µM PCA for 1 h followed by 200 µM palmitic acid for indicated times (A) without or (B) with 5 µg/mL of cycloheximide. Quantification of the expression levels of IκBα and β-actin were analyzed by ImageJ software and the relative amount of each protein was obtained by normalizing it to the β-actin level and expressing it as a ratio vs. 0 min of each group. **(C and D)** MG6 cells pretreated with or without 10 µM PCA for 60 min were treated with or without 200 µM palmitic acid for 120 min, in the presence of MG132 as a 26S proteasome inhibitor. The western blotting analysis was performed to detect the protein expression of **(C)** Ubiquitin and IκBα in the immunoprecipitated IκBα and **(D)** Ubiquitin, IκBα, and glyceraldehyde-3-phosphate dehydrogenase (GAPDH) in the cytoplasm (Input). **(E)** TPCA-01 (IKK inhibitor) and protocatechuic acid (PCA) were mixed with IKKβ recombinant protein at the listed concentrations. IKKβ kinase activity was expressed as relative luciferase units (RLU) based on the fluorescence intensity of luciferin oxidized by ADP-GroTM according to the amount of ADP material converted from ATP. Data shown are mean ± standard deviation (*n* = 3). **(A, B)** Different letters represent significant differences among the three groups at the same time point by Tukey-Kramer honestly significant difference test (*p* < 0.05). **(E)** The asterisks represent significant differences vs. 0 nM group by Dunnett’s test (*p < 0.05).

NF-κB is normally localized in the cytoplasm through the masking of its nuclear translocation signal by IκBα [34,35]. Phosphorylation of IκBα at Ser32/36 leads to polyubiquitination at Lys48 followed by proteasome degradation, resulting in nuclear translocation of NF-κB [36–38]. The expression of IκBα in MG6 cells treated with palmitic acid was significantly lower compared with the BSA control group 2 h after treatment, whereas PCA significantly suppressed the palmitic acid-induced decrease in IκBα expression at 6 h (Fig 5A). To clarify whether PCA inhibited proteasomal degradation of IκBα, MG6 cells were treated with palmitic acid in combination with cycloheximide as a protein synthesis inhibitor. Under these conditions, palmitic acid promoted IκBα degradation even more rapidly, within 15 min, whereas PCA inhibited this degradation to the same level as the BSA control group (Fig 5B). The effect of palmitic acid and PCA on polyubiquitination of IκBα in the presence of MG132, a 26S proteasome inhibitor, was evaluated. Palmitic acid promoted IκBα ubiquitination, whereas pretreatment with PCA inhibited this effect (Fig 5C). In contrast, PCA did not suppress the ubiquitination of IκBα by MG132 in the absence of palmitic acid (Fig 5C). Furthermore, PCA did not affect the ubiquitination of whole cytoplasmic proteins by MG132 (Fig 5D). The phosphorylation of IκBα by the upstream kinase IKK/NEMO complex facilitates its ubiquitin-proteasome degradation. PCA markedly suppressed the kinase activity of recombinant IKK protein at a concentration of just 1 nM, achieving over 50% inhibition at 10 nM (Fig 5E). These results suggest that PCA inhibits the IKK kinase activity, resulting in inhibition of palmitic acid-induced ubiquitin-proteasome degradation of IκBα.

### 3.6. The anti-inflammatory effect of PCA on microglial activation in the arcuate nucleus of the hypothalamus in HFD-fed mice

**Fig 6:**
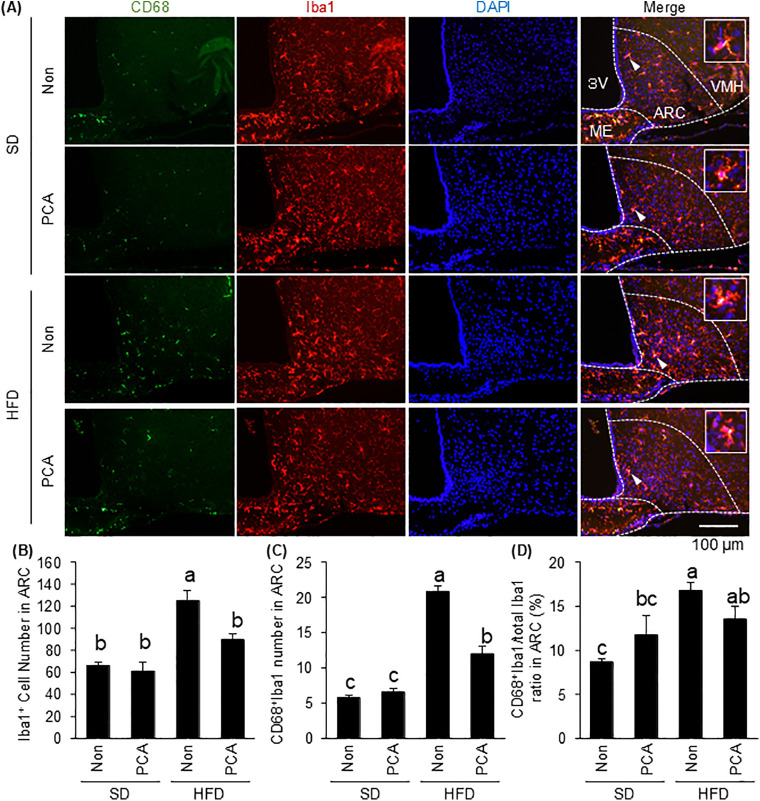
Effects of PCA on the HFD-induced microglial activation in the ARC of the hypothalamus. **(A)** Sections of the mediobasal hypothalamus (10 μm-thick), immunofluorescence-stained for cluster of differentiation 68 (CD68) (green), ionized calcium-binding adapter molecule 1 (Iba1) (red) and nuclei with 4’,6-diamidino-2-phenylindole (DAPI) (blue) in mice fed a standard diet (SD), SD + PCA, high-fat diet (HFD), or HFD + PCA for 4 weeks. The third ventricle (3V), median eminence (ME), hypothalamic arcuate nucleus (ARC), and ventromedial hypothalamic nucleus (VMH) of the left side of the brain of mice from each group are shown. To show the morphology of microglia, a representative microglia was randomly selected and enlarged. The selected microglia were pointed by a white triangle. Quantification of **(B)** the number of Iba1-immunoreactive cells, and **(C)** the number and **(D)** the ratio of CD68-positive Iba1-immunoreactive cells in the ARC. The cell numbers in the ARC of the left and right hemispheres of the brain were measured and mean values were calculated. Data shown are mean ± standard error (*n* = 5). Different letters represent significant differences among the three groups by Tukey-Kramer honestly significant difference test (*p* < 0.05).

**Fig 7:**
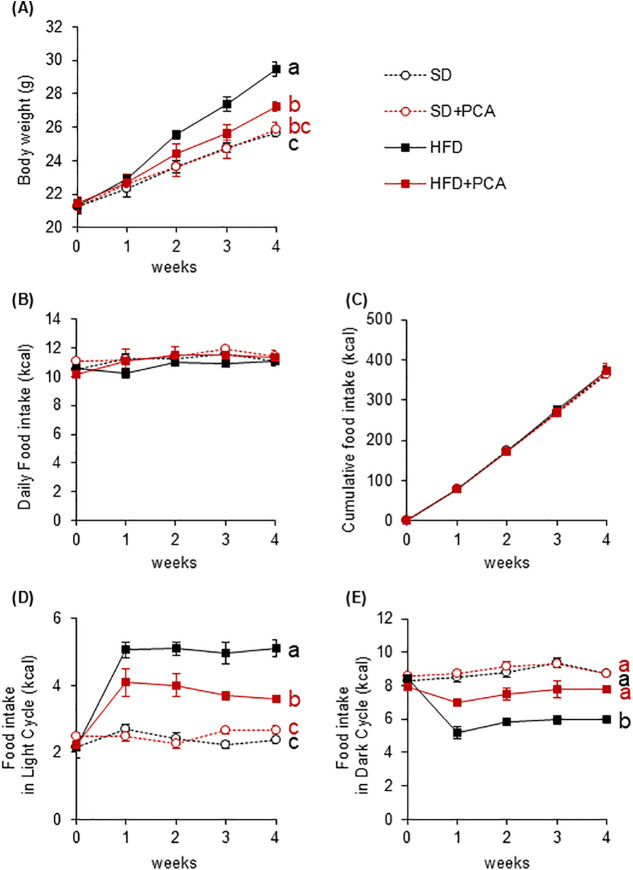
Effects of PCA on the HFD-induced body weight gain and abnormal feeding pattern of mice. **(A)** Changes in body weight in each group during the 4-week study period. Food intake **(B)** daily and **(C)** cumulative food intake; during **(D)** the light and **(E)** dark periods, during the experimental period. Data shown are mean ± standard error (*n* = 5). Different letters represent significant differences among the five groups in each experimental period by the Tukey-Kramer honestly significant difference test (*p* < 0.05). The results of statistical analysis are shown only at the endpoints.

Next, we examined whether PCA suppressed microglial activation in the ARC of the hypothalamus in HFD-fed mice. The number of microglial cells accumulated in the ARC was significantly higher in HFD-fed mice than in SD-fed mice, whereas supplementation with PCA suppressed the accumulation of microglial cells in the ARC induced by HFD ingestion (Figs 6A and 6B). Here, Iba1-positive cells immunoreactive to CD68, which is specifically expressed in phagocytic microglia [28], were defined as activated microglia, and the number and ratio of activated microglia in the ARC were determined. The number and ratio of activated microglia were significantly higher in HFD-fed mice than in SD-fed mice, while supplementation with PCA significantly suppressed the accumulation of activated microglia (Fig 6A, 6C, and 6D). The inflammation of the hypothalamus which is the central regulator of energy metabolism and feeding behavior causes obesity in mice. In this study, the supplementation of PCA, which exerted anti-inflammatory effects, suppressed HFD-induced weight gain (Fig 7A). In all groups, food intake was maintained at approximately 10 kcal/day, with no significant differences in cumulative food intake over the 4 weeks (Figs 7B and 7C). Focusing on the feeding pattern, HFD-fed mice showed increased food intake during the light period and decreased food intake during the dark period, but the intake of PCA corrected these changes (Figs 7D and 7E). These results reproduce the effect of C3G that we previously reported [14], and further reveal that PCA suppresses HFD-induced hypothalamic inflammation *in vivo*.

### 3.7. PCA content in mouse hypothalamus and MG6 cells

**Fig 8:**
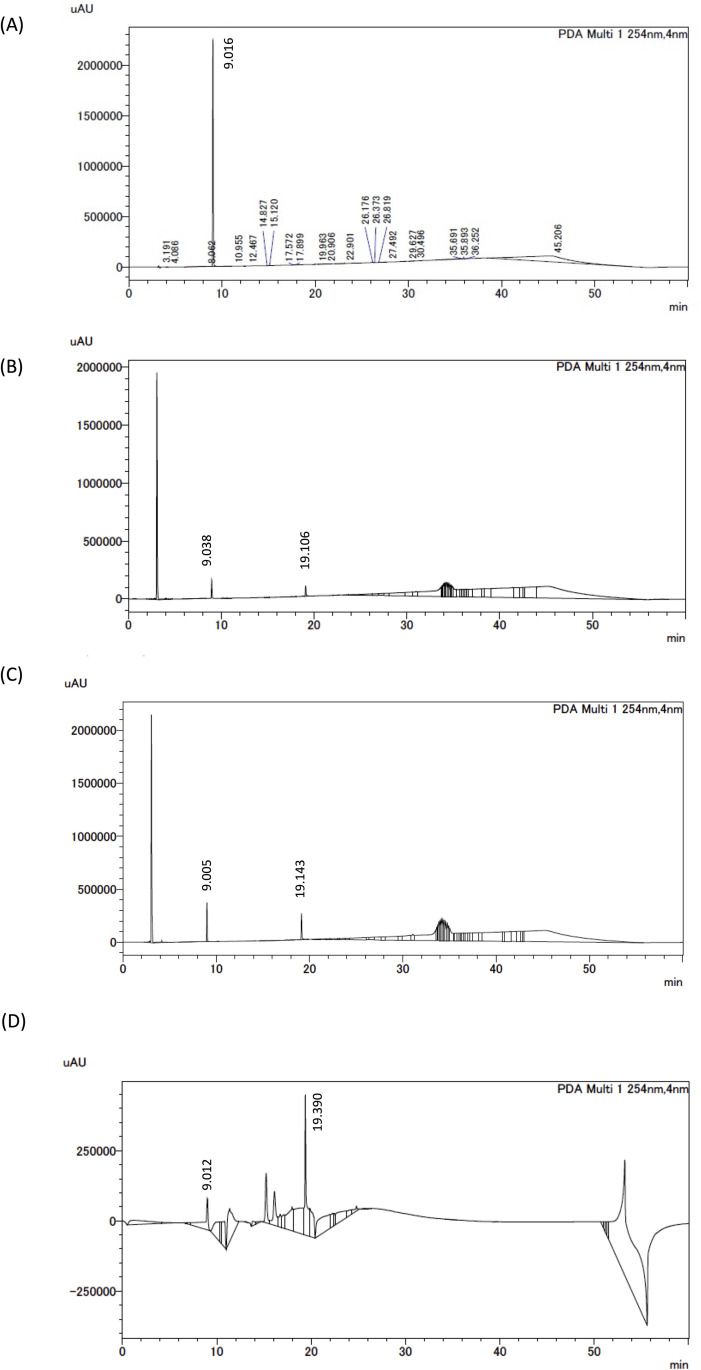
PCA content in mouse hypothalamus and MG6 cells. **(A)** Typical chromatogram of PCA compound. **(B)** Typical chromatogram of MG6 cells treated with 10 μM PCA for 24 hours. **(C)** Typical chromatogram when PCA‌‌ was added to the sample in **(B)**. **(D)** Typical chromatogram in the hypothalamus when mice were fed PCA for 1 week.

The HPLC analysis showed that PCA and quercetin were detected with retention times of approximately 9 (Fig 8A) and 19 min (Figs 8B–8D), respectively. In MG6 cells treated with 10 μM PCA for 24 hours, PCA was detected at a concentration of 31.54 ± 7.21 nmol/mg protein (Fig 8B). The credibility of this result was confirmed by the increase in the peak area corresponding to PCA when the authentic compound was added to the same sample and analyzed by HPLC (Fig 8C). In addition, PCA was detected at the concentration of 0.32 ± 0.11 nmol/mg tissue in the hypothalamus of mice fed an HFD containing 2% of PCA for one week. (Fig 8D). These results indicated that the ingested PCA reached the mouse hypothalamus and microglial cells could take up PCA.

## 4. Discussion

It is known that HFDs, especially those containing LCSFAs such as palmitic acid, cause inflammation and microglial activation in the ARC, leading to obesity due to disruption of the feeding rhythm and metabolic disorders in peripheral tissues. The present study found that among of C3G and its major metabolites, PCA most strongly suppressed palmitic acid-induced inflammatory responses in murine microglia. In accordance with this finding, supplementation with PCA in mice corrected HFD-induced feeding alterations and prevented obesity. PCA is a type of widely distributed naturally occurring phenolic acid and more than 500 plants contain PCA as active constituents imparting various pharmacological activity. PCA has been widely studied for its vast nutraceutical benefits [39]. However, the effect of PCA on inflammation and activation of microglia cells in the ARC was unknown. In this study, it was first demonstrated that, PCA suppress the expression of inflammation-related factors mediated by NF-κB through inhibition of IKK in microglia cells.

AAPH-induced intracellular ROS production stimulated microglial cells to release TNF-alpha, whereas 8 compounds shown in Fig 1A, which known as major metabolites of C3G, inhibited ROS-induced cytokine release in microglia (Figs 2C and 2D). However, the LCSFAs-induced inflammation did not appear to be mediated by intracellular ROS production (Figs 1C, 2E and 2F). Previous research suggests‌‌ that fatty acids facilitate the stimulation of forward electron flow by mediating the uncoupling of the inner mitochondrial membrane, thereby inhibiting ROS generation at Complex I during reverse electron transport [40,41]. Although the seven compounds shown in Fig 1A had strong antioxidant activity (Figs 2A and 2B), only PCA suppressed the inflammatory response of microglial cells induced by palmitic acid (Figs 1D and 1E). These results suggest that the mechanisms of antioxidant activity and the anti-inflammatory effects of polyphenols should be considered separately.

PCA suppressed the expression of pro-inflammatory cytokines in microglia induced by LCSFAs, but vanillic acid and isovanilic acid had no effect. PCA possesses a catechol structure characterized by the presence of two adjacent phenolic hydroxyl groups on the benzene ring. In the case of vanillic acid and isovanilic acid, however, one of the two hydroxyl groups undergoes methoxylation. Therefore, the catechol structure is likely the key to the anti-inflammatory effects of PCA. However, compounds like C3G and its aglycone cyanidin, which also possess a catechol structure, did not show anti-inflammatory effects. The overall electrostatic potential and characteristics of molecules are significantly influenced not only by their catechol structure but also the diverse functional groups bound to the benzene ring [42]. Comparing the anti-inflammatory effects of various analogous using PCA as a benchmark may lead to the discovery of more effective compounds. In addition, clarifying the *in vivo* dynamics of these compounds and undertaking further verification of their effects may lead to the discovery of food components or compounds that are more effective in inhibiting hypothalamic inflammation.

Investigating whether PCA reaches the brain is important, as many functional compounds have been reported to have difficulty crossing the blood-brain barrier. In this study, we detected PCA in the hypothalamus of mice following oral ingestion. Our findings align with previous reports showing that PCA was detected in the mouse brain at a concentration of 0.24 ± 0.09 nmol/mg protein, where it suppressed brain glycation and inflammation [43]. The same study demonstrated that PCA can cross the blood-brain barrier. Another study reported that PCA reaches the brain and suppresses microglial activation [39]. Since humans and mice lack the enzyme required for PCA metabolism, PCA is absorbed from the digestive tract and circulates in its intact form in both species [44,45]. These findings, along with our results, suggest that the observed effects of PCA in the brain are directly attributable to its presence in microglial cells in the hypothalamus. However, further study is needed to elucidate how PCA reaches the ARC in the brain. While PCA reaches the brain in mice, there are no published studies yet on whether PCA reaches the human brain. Another study reported that when humans ingested approximately 40 mg of PCA, the maximum blood concentration exceeded 3 µM [46]. Additionally, a maximum blood concentration of PCA was detected at 146 nM in humans who ingested 500 mg of C3G [16]. Taken together, these findings highlight the need for further studies to validate the effects of PCA in humans at feasible dietary concentrations.

Hypothalamic inflammation induced by HFD intake may result not only from the direct action of circulating LCSFAs, but also the propagation of inflammatory signals in the gastrointestinal tract mediated through the circulation and the vagal afferent nerves [47,48]. HFDs stimulate the gut microbiota to release gastrointestinal endotoxins and harm tight junctions within the intestinal mucosa increasing permeability [49,50]. In addition, HFD-exposed intestinal macrophages release inflammatory factors, causing inflammation to spread not only to surrounding tissues, but also to organs throughout the body [49]. The experimental design established in the present study assumes that PCA directly reach microglia in the ARC. However, it is also plausible that PCA could effectively inhibit the initiation and propagation of inflammatory signals in the intestinal tract. For instance, some previous studies show that C3G and PCA prevent HFD-induced degradation of gastrointestinal tight junctions, reduce intestinal endotoxins, and suppress gastrointestinal inflammation [51–53]. Therefore, future studies should consider inter-organ communication, including the interaction with the central nervous system. Finally, this study did not assess sex-specific differences. Male mice are generally more susceptible to HFD-induced metabolic alterations than females [54,55], and microglial functions are also known to exhibit sexual dimorphism [56]. In the present study, MG6 microglial cells were isolated from postnatal day 1 mice, and the sex of the animals was not determined. Thus, potential sex-dependent effects should be considered when interpreting the results.

## 5. Conclusion

Oral consumption of PCA was found to suppress hypothalamic inflammation induced by a HFD in mice, thereby preventing abnormal feeding behavior and obesity. PCA was also detected in the hypothalamus. In addition, in an *in vitro* study, PCA suppressed LCSFAs-induced inflammation in murine microglia via inhibition of the NF-κB pathway in HFD-fed mice. These findings provide new evidence and insights into the mechanisms by which polyphenols, including PCA and its analogs, ameliorate diet-induced obesity.

## Supporting information

S1 Fig Identification of fatty acids that induce inflammatory responses in MG6 cells. Palmitic acid (C16:0), stearic acid (C18:0), oleic acid (C18:1), linoleic acid (C18:2), and α-linolenic acid (C18:3) were treated to MG6 cells. (A) The amount of TNF-alpha released into the medium from MG6 cells treated with the listed fatty acids for 24 h was measured by ELISA. (B) The expression levels of inflammatory cytokines (*Tnf-alpha*, *Il1b*, *Il6*) and chemokine (*Ccl2*) in MG6 cells treated with the listed fatty acids for 6 h were analyzed by quantitive RT-PCR. Each value was normalized to baseline (0 h). Values over the highest value of the graph are noted as actual values on each bar. (A and B) The asterisks represent significant differences vs. the BSA group by Dunnett’s test (*p < 0.05).
(TIF)

S2 Fig Effects of palmitic acid and LPS on phosphorylation and degradation of IκBα in MG6 cells. (A and B) The MG6 cells were treated with 200 µM palmitic acid (C16:0) or 1 µg/mL LPS and then harvested at the timings indicated in this Figure. The whole cell lysates were subjected to Western blotting.
(TIF)

S3 Fig Impact of JNK or NF-κB silencing on the upregulation of Tnf-alpha expression triggered by palmitic acid in MG6 cells. (A and B) Control siRNA and siRNA targeted for JNK or NF-κB (sequences shown in Section 2.2) were transfected into MG6 cells by lipofection, and whole cell lysates were subjected to Western blotting 24 h later. (C) MG6 cells transfected with the siRNAs were treated with 200 µM palmitic acid for 6 h, and the expression level of *Tnf-alpha* was quantified by RT-PCR. Data shown are mean ± standard deviation (*n* = 3). The asterisks represent significant differences vs. BSA group of each by two-tailed t-test (*p < 0.05).
(TIF)

S1 Table Antibody list for Western blotting. (DOCX)

S2 Table Antibody list for Immunocytochemistry. (DOCX)

S3 Table Antibody list for Immunohistchemistry. (DOCX)

S1 File RAW blots for Main Data & Review_Hironao.pptx. Unprocessed raw data and corresponding membrane images of Western blotting experiments used for the main figures.
(PPTX)

S2 File RAW bolts for statistics_Hironao.pptx. Unprocessed raw data and corresponding membrane images of Western blotting experiments used for protein expression quantification and statistical analyses in the main figures.
(PPTX)

## Abbreviations

AAPH: 2,2′-azobis (2-amidinopropane) dihydrochloride
ARC: arcuate nucleus
C3G: cyanidin 3-*O*-glucoside
HFD: high-fat diet
LCSFAs: I kappa B kinase
IKK: long-chain saturated fatty acids
LPS: lipopolysaccharide
PCA: protocatechuic acid
ROS: reactive oxygen species
SD: standard diet
