# Supplementary figures and images for: Protocatechuic acid prevents obesity caused by long-chain saturated fatty acid-induced inflammation in mouse microglia via inhibition of the NF-κB pathway
Source: PLoS One. 2026 Jun 1;21(6):e0347055. doi: 10.1371/journal.pone.0347055 (PMC13225654; doi:10.1371/journal.pone.0347055)

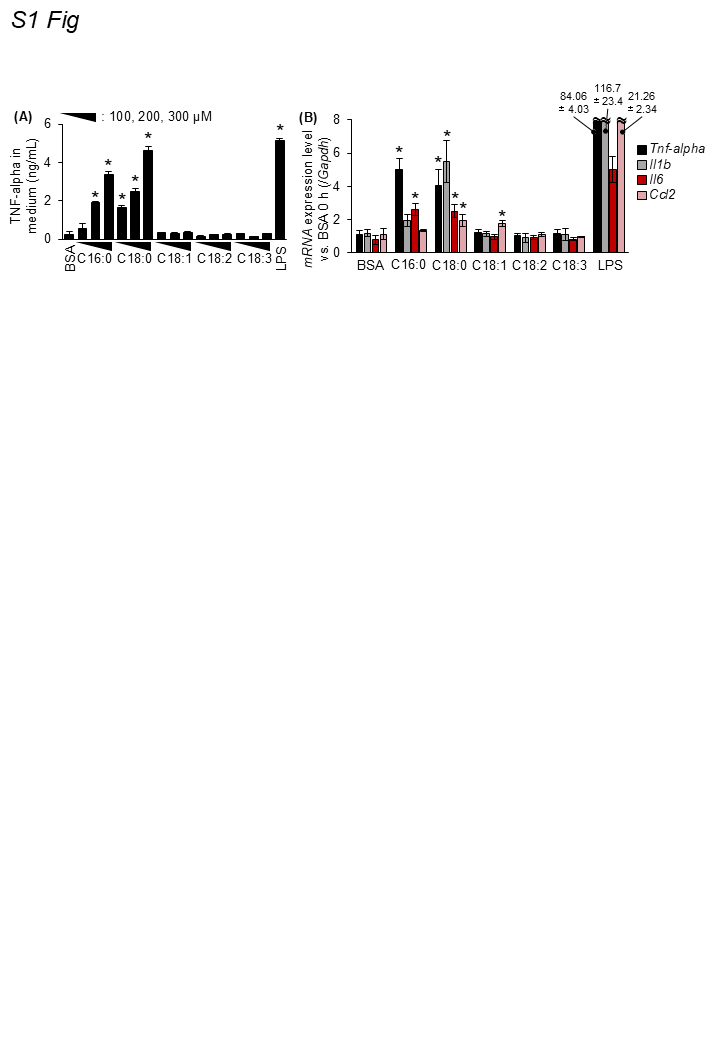

Supplement: S1 Fig — Palmitic acid (C16:0), stearic acid (C18:0), oleic acid (C18:1), linoleic acid (C18:2), and α-linolenic acid (C18:3) were treated to MG6 cells. (A) The amount of TNF-alpha released into the medium from MG6 cells treated with the listed fatty acids for 24 h was measured by ELISA. (B) The expression levels of inflammatory cytokines (Tnf-alpha, Il1b, Il6) and chemokine (Ccl2) in MG6 cells treated with the listed fatty acids for 6 h were analyzed by quantitive RT-PCR. Each value was normalized to baseline (0 h). Values over the highest value of the graph are noted as actual values on each bar. (A and B) The asterisks represent significant differences vs. the BSA group by Dunnett’s test (*p < 0.05). (TIF) [file pone.0347055.s001.tif]

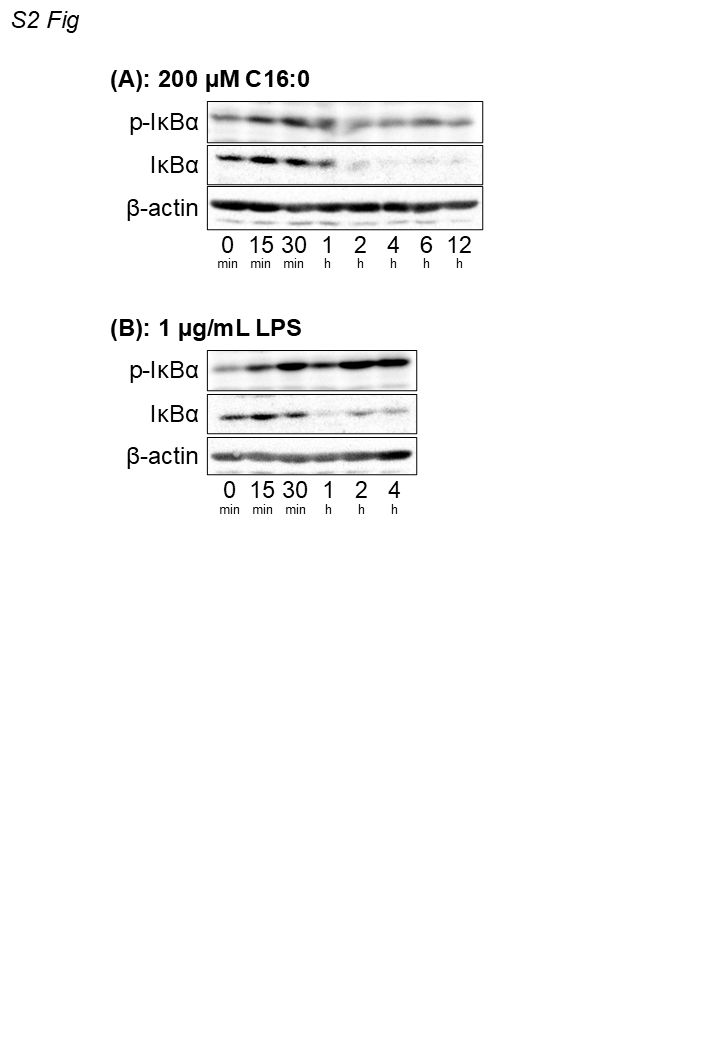

Supplement: S2 Fig — (A and B) The MG6 cells were treated with 200 µM palmitic acid (C16:0) or 1 µg/mL LPS and then harvested at the timings indicated in this Figure. The whole cell lysates were subjected to Western blotting. (TIF) [file pone.0347055.s002.tif]

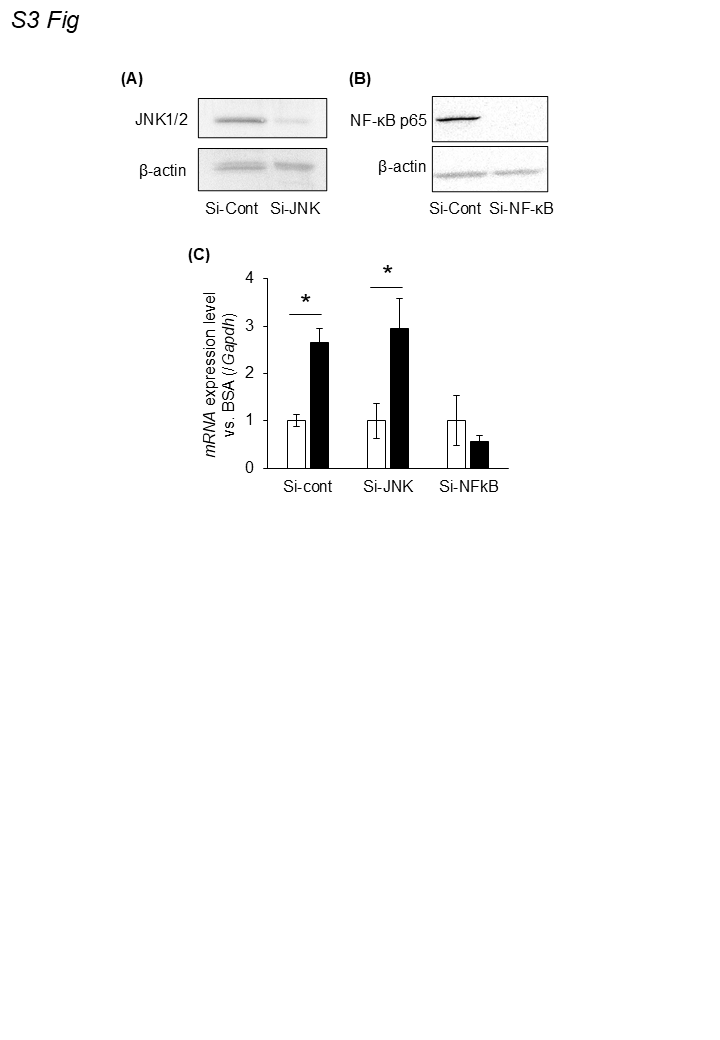

Supplement: S3 Fig — (A and B) Control siRNA and siRNA targeted for JNK or NF-κB (sequences shown in Section 2.2) were transfected into MG6 cells by lipofection, and whole cell lysates were subjected to Western blotting 24 h later. (C) MG6 cells transfected with the siRNAs were treated with 200 µM palmitic acid for 6 h, and the expression level of Tnf-alpha was quantified by RT-PCR. Data shown are mean ± standard deviation (n = 3). The asterisks represent significant differences vs. BSA group of each by two-tailed t-test (*p < 0.05). (TIF) [file pone.0347055.s003.tif]
