# Supplementary material for: Protocatechuic acid prevents obesity caused by long-chain saturated fatty acid-induced inflammation in mouse microglia via inhibition of the NF-κB pathway
Source: PLoS One. 2026 Jun 1;21(6):e0347055. doi: 10.1371/journal.pone.0347055 (PMC13225654; doi:10.1371/journal.pone.0347055)
Supplement: S1 Table — (DOCX) [file pone.0347055.s004.docx]

S1 Table

| Product | Cat No. | Manufacture | Working dillution |
| --- | --- | --- | --- |
| anti-β-Actin rabbit polyclonal antibody | 4967 | CST | 1:5000 |
| anti-GAPDH rabbit monoclonal (14C10) antibody | 2118 | CST | 1:5000 |
| anti-Lamin B1 mouse monoclonal (B-10) antibody | sc-374015 | SantaCrus | 1:5000 |
| anti-Ubiquitin rabbit polyclonal antibody | 3933 | CST | 1:5000 |
| anti-Erk1/2 rabbit polyclonal antibody | 9102 | CST | 1:5000 |
| anti-JNK rabbit polyclonal antibody | 9252 | CST | 1:5000 |
| anti-NF-κB p65 rabbit polyclonal antibody | 3034 | CST | 1:5000 |
| anti-IκBα rabbit polyclonal antibody | 9242 | CST | 1:5000 |
| anti-phospho-Erk1/2 (Thr202/Tyr204) rabbit polyclonal antibody | 9101 | CST | 1:5000 |
| anti-phospho-JNK (Thr183/Tyr185) rabbit polyclonal antibody | 9251 | CST | 1:5000 |
| anti-phospho-NF-κB p65 (Ser536) Rabbit monoclonal (93H1) antibody | 3033 | CST | 1:5000 |
| anti-phospho-IκBα (Ser32/36) mouse monoclonal (5A5) antibody | 9246 | CST | 1:5000 |
| anti-rabbit IgG, HRP-linked antibody | 7074 | CST | 1:20000 |
| anti-mouse IgG, HRP-linked antibody | 7076 | CST | 1:20000 |
