# Supplementary material for: Protocatechuic acid prevents obesity caused by long-chain saturated fatty acid-induced inflammation in mouse microglia via inhibition of the NF-κB pathway
Source: PLoS One. 2026 Jun 1;21(6):e0347055. doi: 10.1371/journal.pone.0347055 (PMC13225654; doi:10.1371/journal.pone.0347055)
Supplement: S3 Table — (DOCX) [file pone.0347055.s006.docx]

S3 Table

| Product | Cat No. | Manufactures | Working dillution |
| --- | --- | --- | --- |
| anti-Iba1 mouse monoclonal (EPR16589) antibody | ab283319 | Abcam | 1:200 |
| anti-CD68 rabbit monoclonal (E3O7V) antibody | 97778 | CST | 1:1000 |
| Alexa Fluor™ 488-conjugated goat anti-rabbit IgG | A-11008 | Invitrogen | 1:1000 |
| Alexa Fluor™ 594-conjugated goat anti-mouse IgG | A-11005 | Invitrogen | 1:1000 |
