# Supplementary material for: Protocatechuic acid prevents obesity caused by long-chain saturated fatty acid-induced inflammation in mouse microglia via inhibition of the NF-κB pathway
Source: PLoS One. 2026 Jun 1;21(6):e0347055. doi: 10.1371/journal.pone.0347055 (PMC13225654; doi:10.1371/journal.pone.0347055)
Supplement: S1 File — Unprocessed raw data and corresponding membrane images of Western blotting experiments used for the main figures. (PPTX) [file pone.0347055.s007.pptx]

## Slide 1
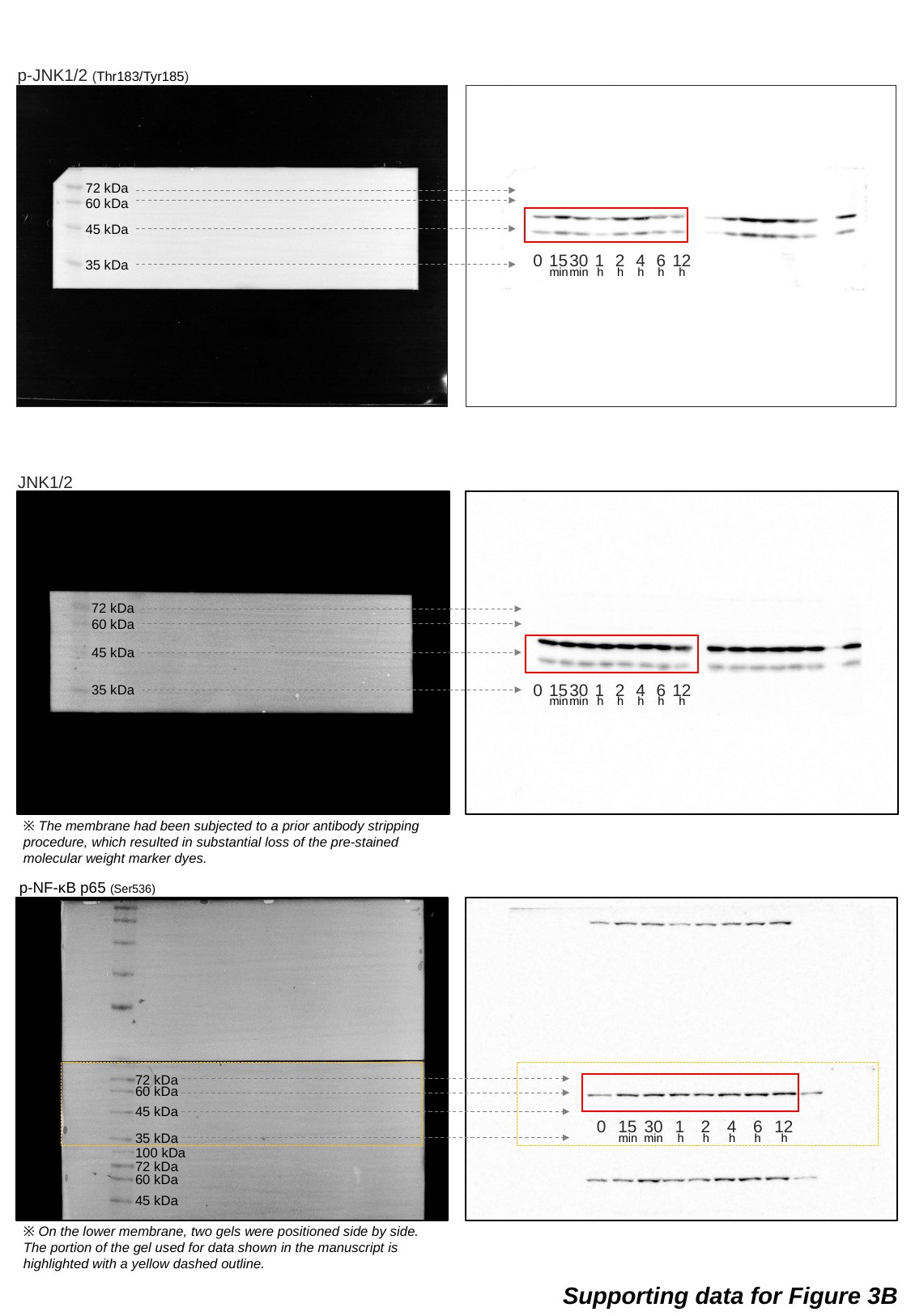

p-JNK1/2 (Thr183/Tyr185)
72 kDa
60 kDa
45 kDa
0
15
30
1
2
4
6
12
min
min
h
h
h
h
h
35 kDa
JNK1/2
72 kDa
60 kDa
45 kDa
0
15
30
1
2
4
6
12
min
min
h
h
h
h
h
35 kDa
※ The membrane had been subjected to a prior antibody stripping procedure, which resulted in substantial loss of the pre-stained molecular weight marker dyes.
p-NF-κB p65 (Ser536)
72 kDa
60 kDa
45 kDa
0
15
30
1
2
4
6
12
min
min
h
h
h
h
h
35 kDa
100 kDa
72 kDa
60 kDa
45 kDa
※ On the lower membrane, two gels were positioned side by side. The portion of the gel used for data shown in the manuscript is highlighted with a yellow dashed outline.
Supporting data for Figure 3B

## Slide 2
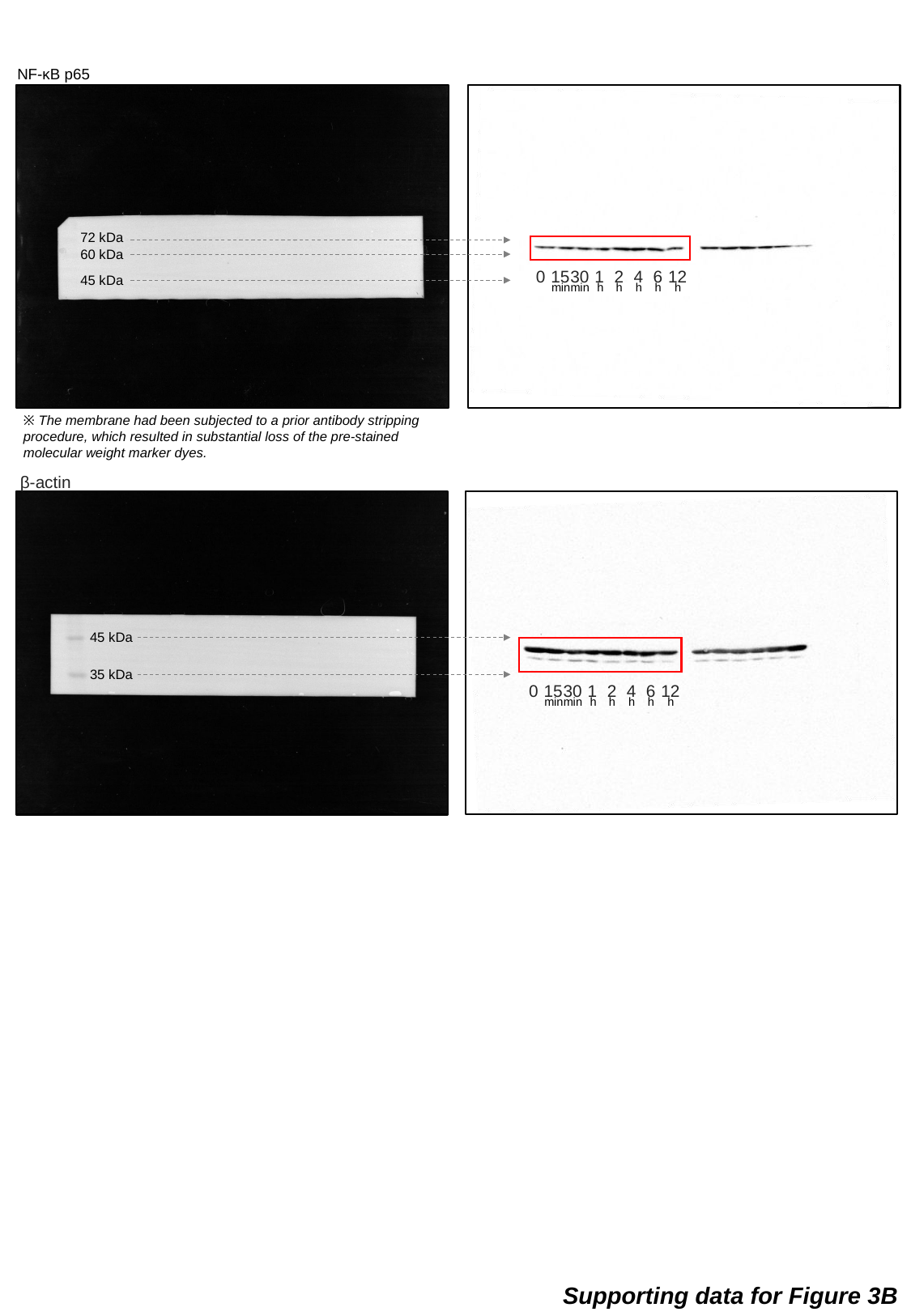

NF-κB p65
72 kDa
60 kDa
0
15
30
1
2
4
6
12
min
min
h
h
h
h
h
45 kDa
※ The membrane had been subjected to a prior antibody stripping procedure, which resulted in substantial loss of the pre-stained molecular weight marker dyes.
β-actin
45 kDa
35 kDa
0
15
30
1
2
4
6
12
min
min
h
h
h
h
h
Supporting data for Figure 3B

## Slide 3
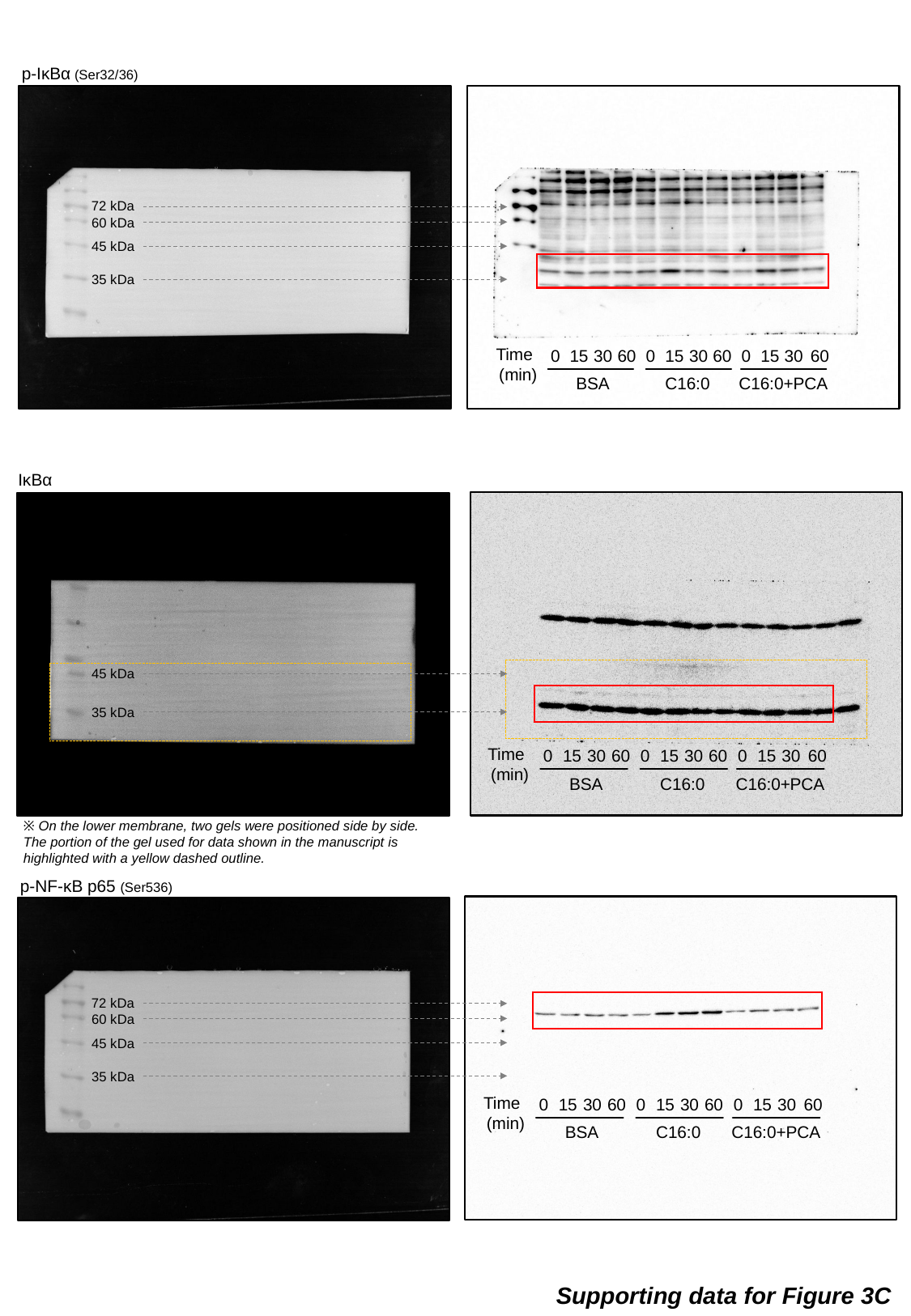

p-IκBα (Ser32/36)
72 kDa
60 kDa
45 kDa
35 kDa
Time
(min)
0
15
30
60
0
15
30
60
0
15
30
60
BSA
C16:0
C16:0+PCA
IκBα
45 kDa
35 kDa
Time
(min)
0
15
30
60
0
15
30
60
0
15
30
60
BSA
C16:0
C16:0+PCA
※ On the lower membrane, two gels were positioned side by side. The portion of the gel used for data shown in the manuscript is highlighted with a yellow dashed outline.
p-NF-κB p65 (Ser536)
72 kDa
60 kDa
45 kDa
35 kDa
Time
(min)
0
15
30
60
0
15
30
60
0
15
30
60
BSA
C16:0
C16:0+PCA
Supporting data for Figure 3C

## Slide 4
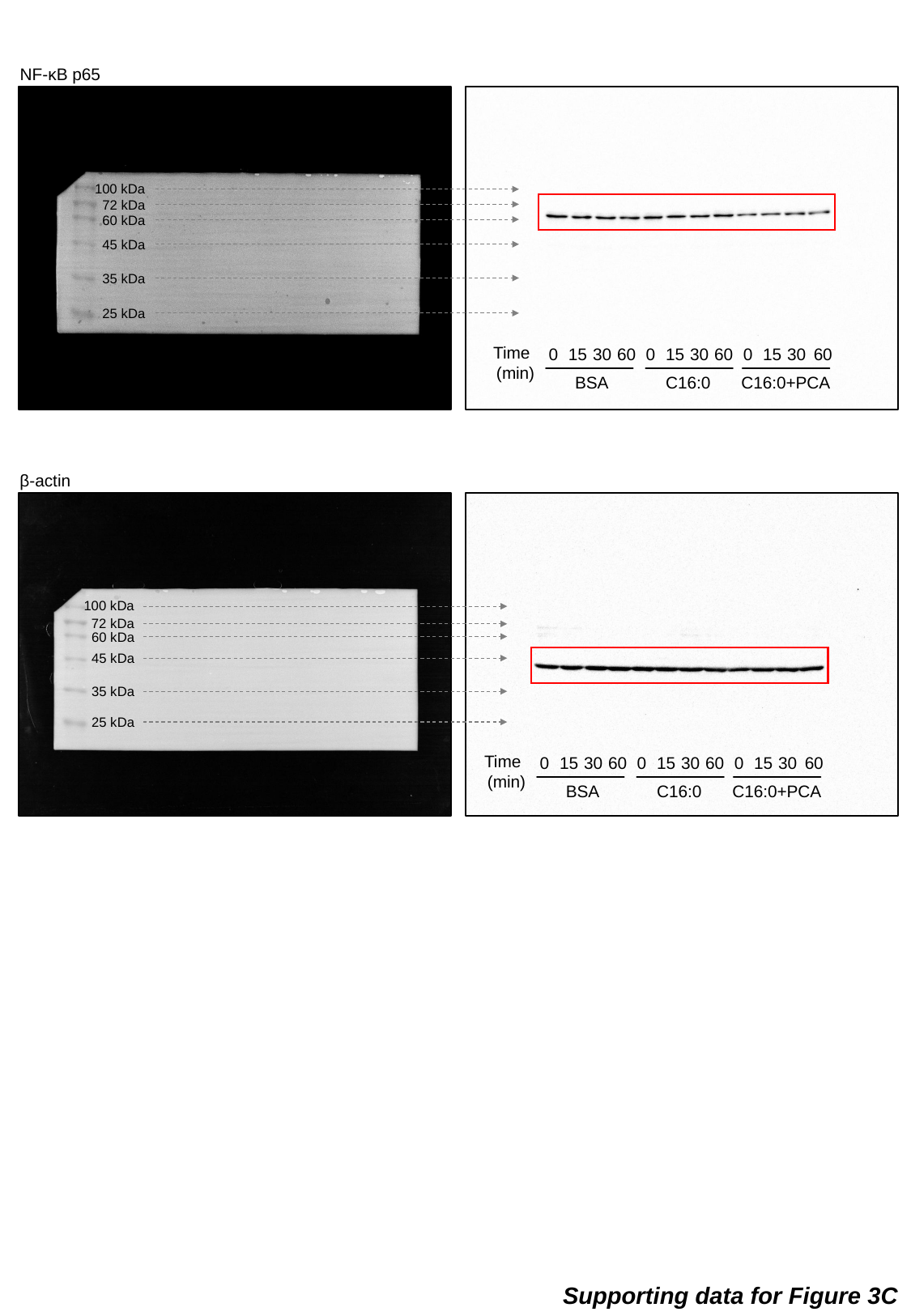

NF-κB p65
100 kDa
72 kDa
60 kDa
45 kDa
35 kDa
25 kDa
Time
(min)
0
15
30
60
0
15
30
60
0
15
30
60
BSA
C16:0
C16:0+PCA
β-actin
100 kDa
72 kDa
60 kDa
45 kDa
35 kDa
25 kDa
Time
(min)
0
15
30
60
0
15
30
60
0
15
30
60
BSA
C16:0
C16:0+PCA
Supporting data for Figure 3C

## Slide 5
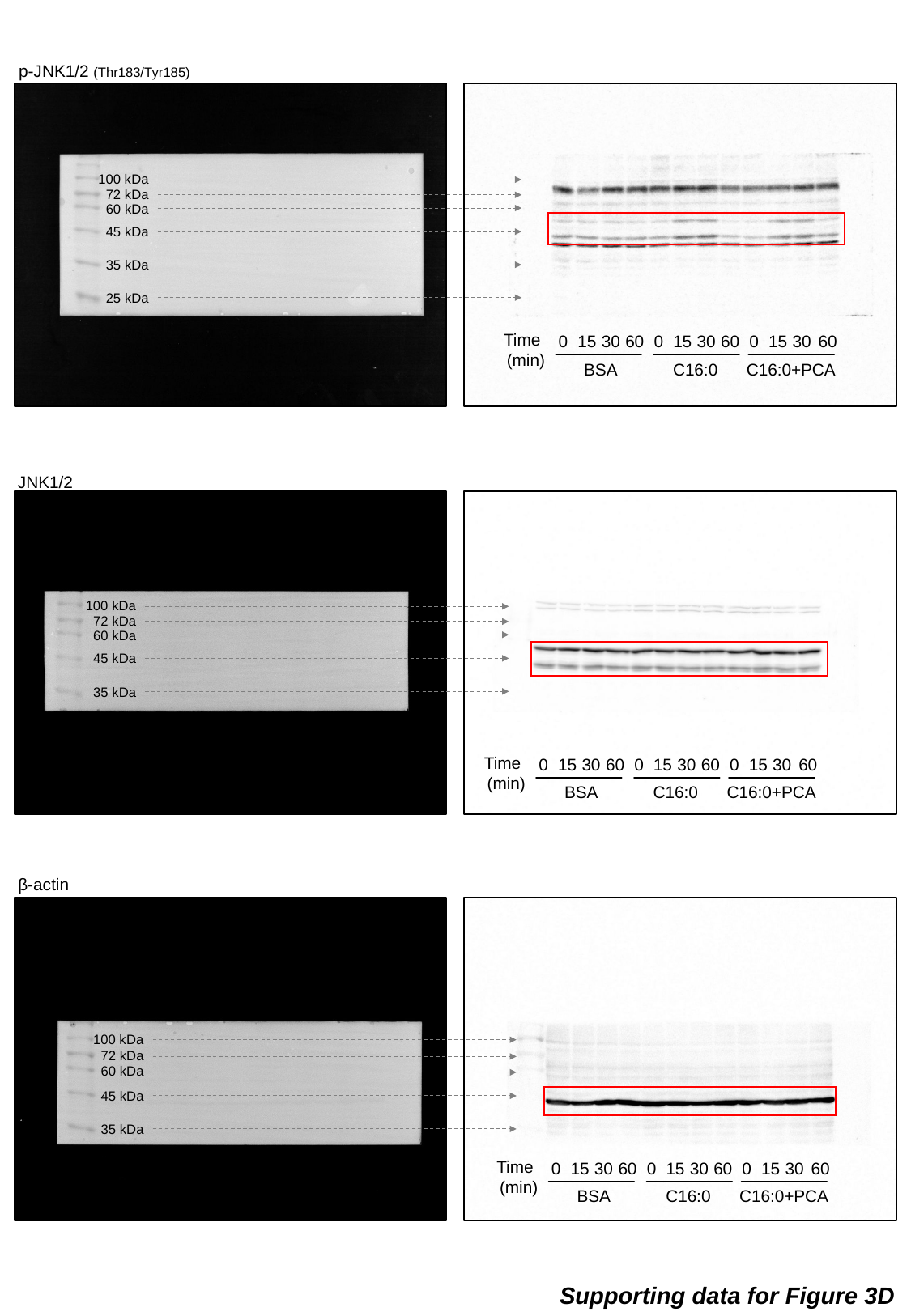

p-JNK1/2 (Thr183/Tyr185)
100 kDa
72 kDa
60 kDa
45 kDa
35 kDa
25 kDa
Time
(min)
0
15
30
60
0
15
30
60
0
15
30
60
BSA
C16:0
C16:0+PCA
JNK1/2
100 kDa
72 kDa
60 kDa
45 kDa
35 kDa
Time
(min)
0
15
30
60
0
15
30
60
0
15
30
60
BSA
C16:0
C16:0+PCA
β-actin
100 kDa
72 kDa
60 kDa
45 kDa
35 kDa
Time
(min)
0
15
30
60
0
15
30
60
0
15
30
60
BSA
C16:0
C16:0+PCA
Supporting data for Figure 3D

## Slide 6
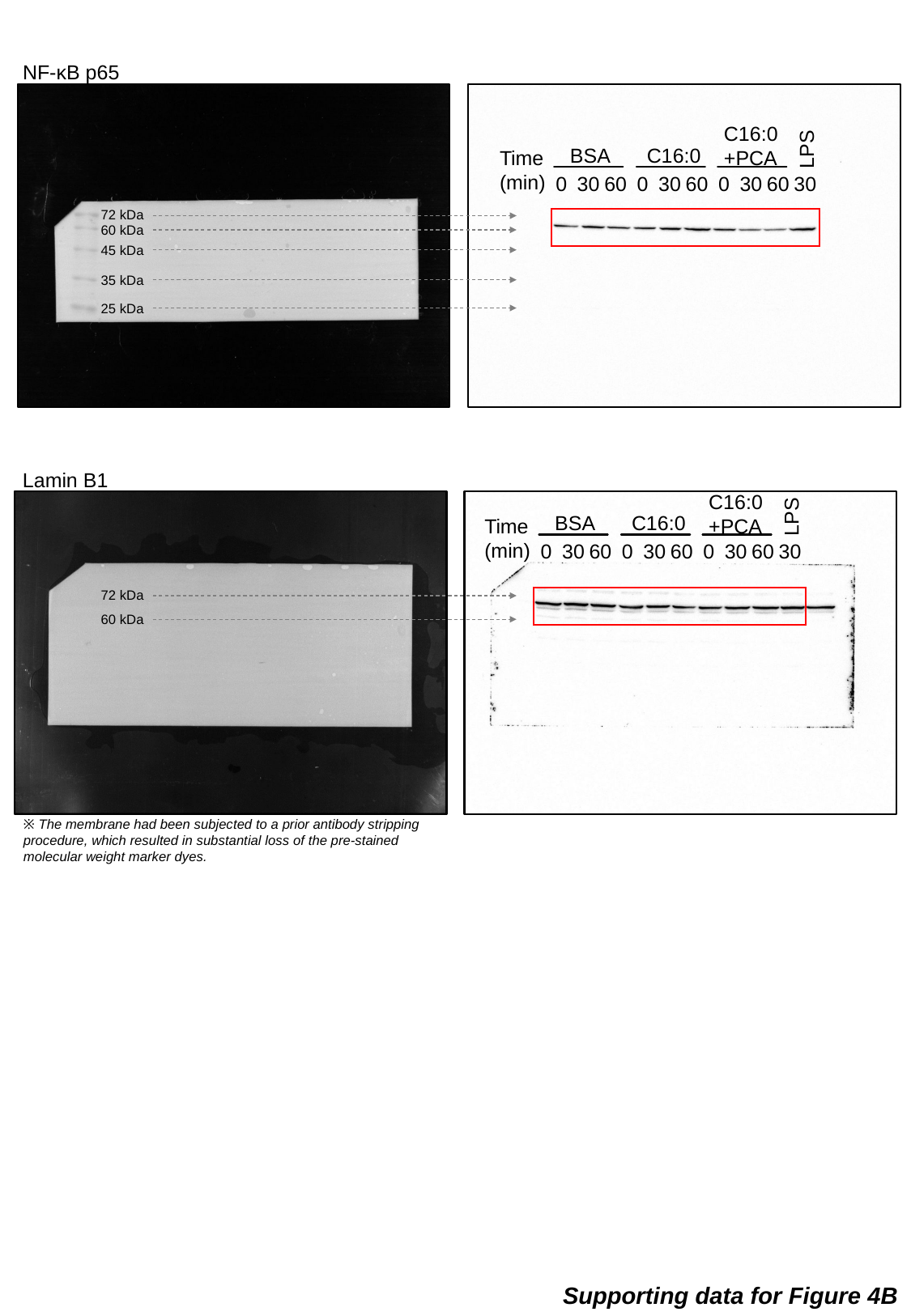

NF-κB p65
C16:0
+PCA
LPS
BSA
C16:0
Time
(min)
0
30
60
0
30
60
0
30
60
30
72 kDa
60 kDa
45 kDa
35 kDa
25 kDa
Lamin B1
C16:0
+PCA
LPS
BSA
C16:0
Time
(min)
0
30
60
0
30
60
0
30
60
30
72 kDa
60 kDa
※ The membrane had been subjected to a prior antibody stripping procedure, which resulted in substantial loss of the pre-stained molecular weight marker dyes.
Supporting data for Figure 4B

## Slide 7
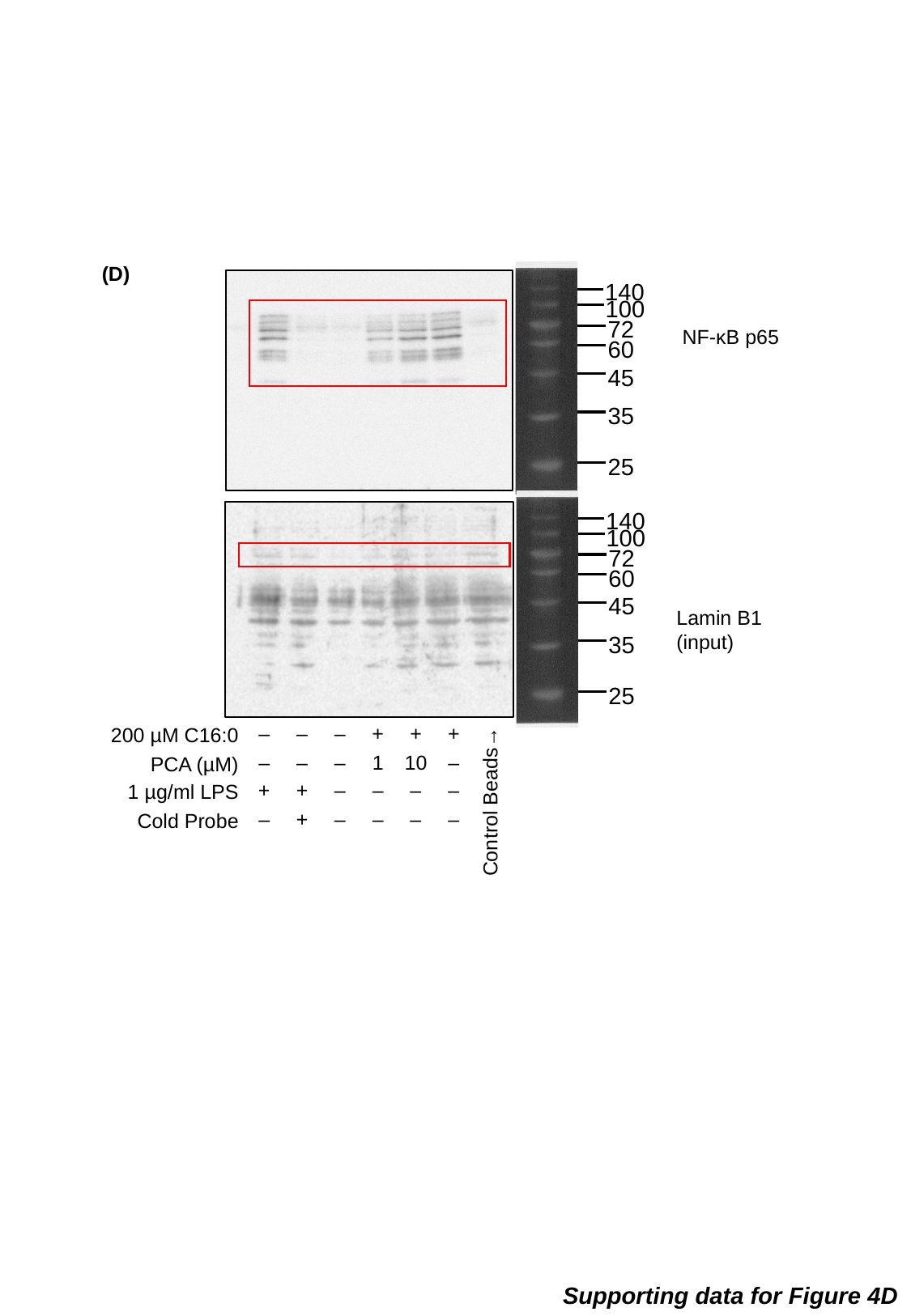

(D)
140
100
72
60
45
35
25
NF-κB p65
140
100
72
60
45
35
25
Lamin B1
(input)
200 µM C16:0
–
–
–
+
+
+
PCA (µM)
–
–
–
1
10
–
1 µg/ml LPS
+
+
–
–
–
–
Control Beads→
Cold Probe
–
+
–
–
–
–
Supporting data for Figure 4D

## Slide 8
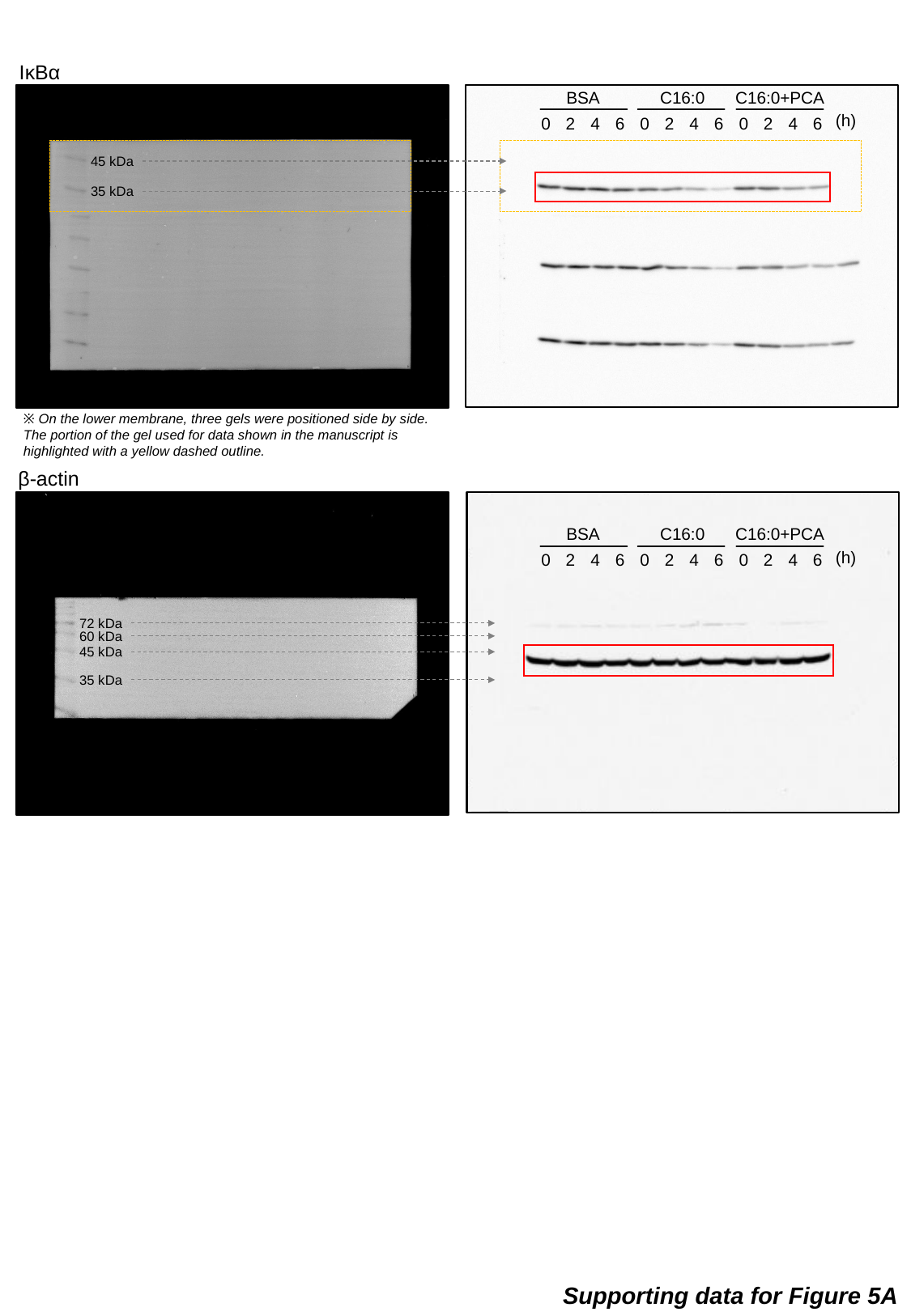

IκBα
BSA
C16:0
C16:0+PCA
(h)
0
2
4
6
0
2
4
6
0
2
4
6
45 kDa
35 kDa
※ On the lower membrane, three gels were positioned side by side. The portion of the gel used for data shown in the manuscript is highlighted with a yellow dashed outline.
β-actin
BSA
C16:0
C16:0+PCA
(h)
0
2
4
6
0
2
4
6
0
2
4
6
72 kDa
60 kDa
45 kDa
35 kDa
Supporting data for Figure 5A

## Slide 9
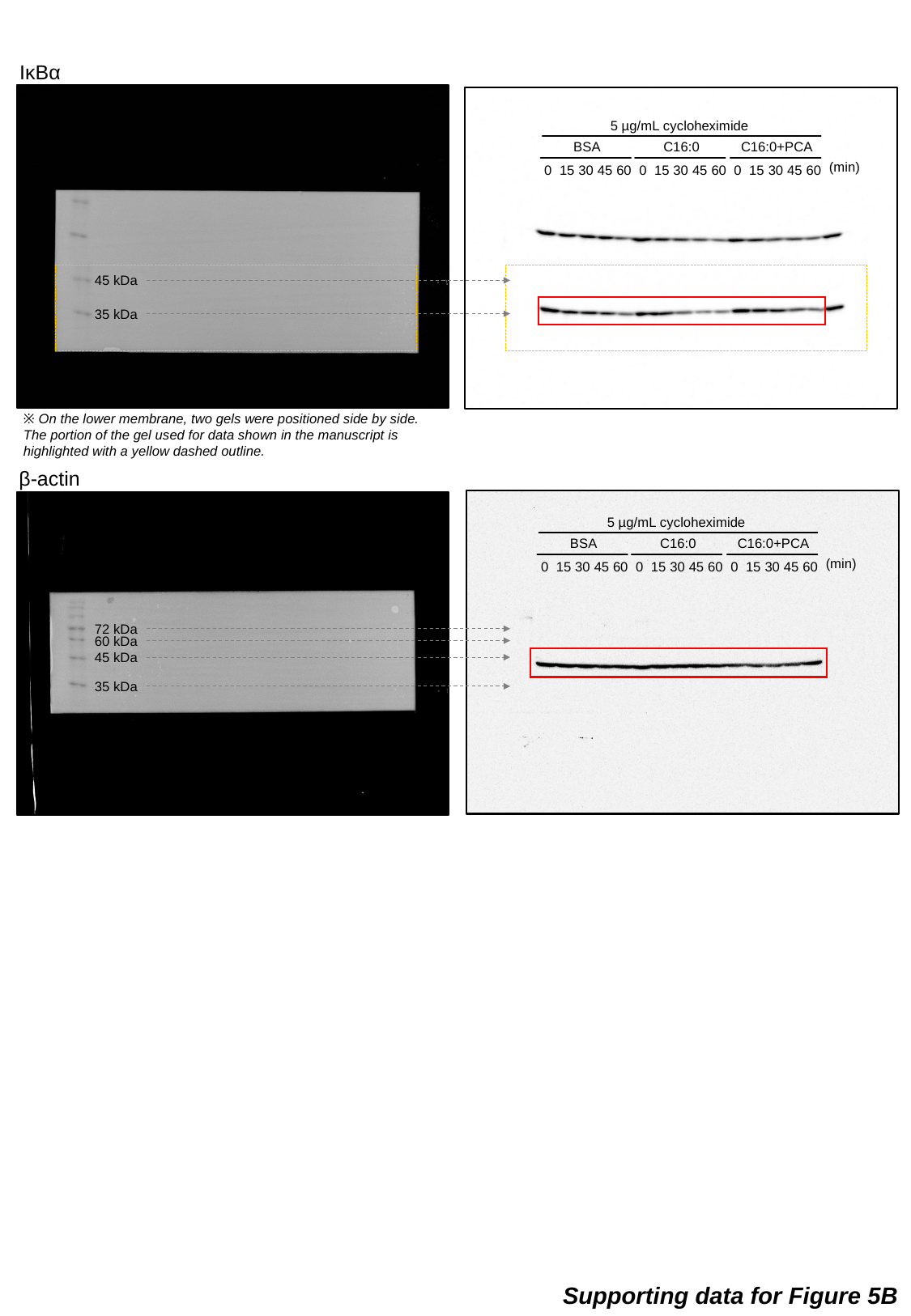

IκBα
5 µg/mL cycloheximide
BSA
C16:0
C16:0+PCA
(min)
0
15
30
45
60
0
15
30
45
60
0
15
30
45
60
45 kDa
35 kDa
※ On the lower membrane, two gels were positioned side by side. The portion of the gel used for data shown in the manuscript is highlighted with a yellow dashed outline.
β-actin
5 µg/mL cycloheximide
BSA
C16:0
C16:0+PCA
(min)
0
15
30
45
60
0
15
30
45
60
0
15
30
45
60
72 kDa
60 kDa
45 kDa
35 kDa
Supporting data for Figure 5B

## Slide 10
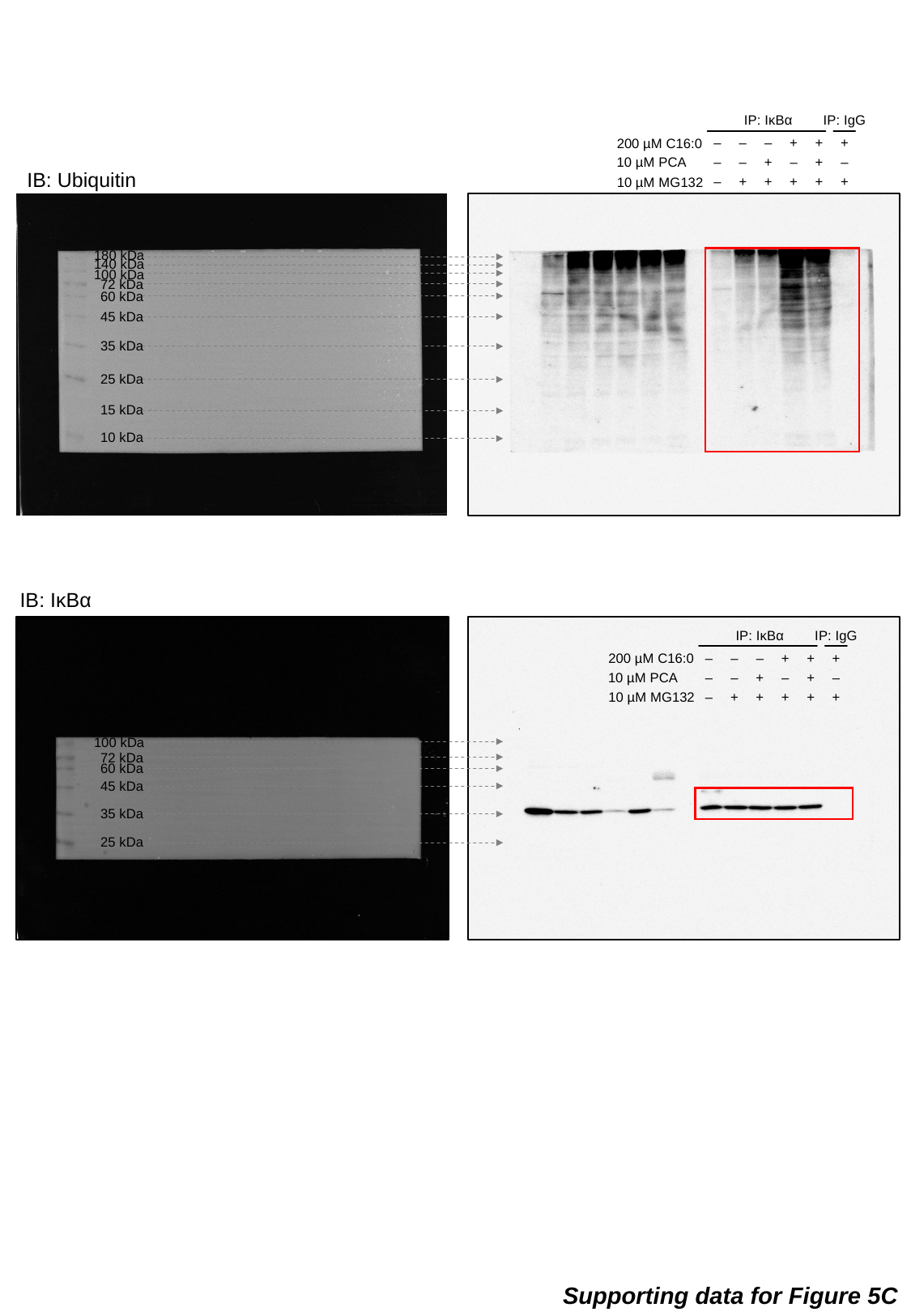

IP: IκBα
IP: IgG
–
–
–
+
+
+
200 µM C16:0
–
–
+
–
+
–
10 µM PCA
–
+
+
+
+
+
10 µM MG132
IB: Ubiquitin
180 kDa
140 kDa
100 kDa
72 kDa
60 kDa
45 kDa
35 kDa
25 kDa
15 kDa
10 kDa
IB: IκBα
IP: IκBα
IP: IgG
–
–
–
+
+
+
200 µM C16:0
–
–
+
–
+
–
10 µM PCA
–
+
+
+
+
+
10 µM MG132
100 kDa
72 kDa
60 kDa
45 kDa
35 kDa
25 kDa
Supporting data for Figure 5C

## Slide 11
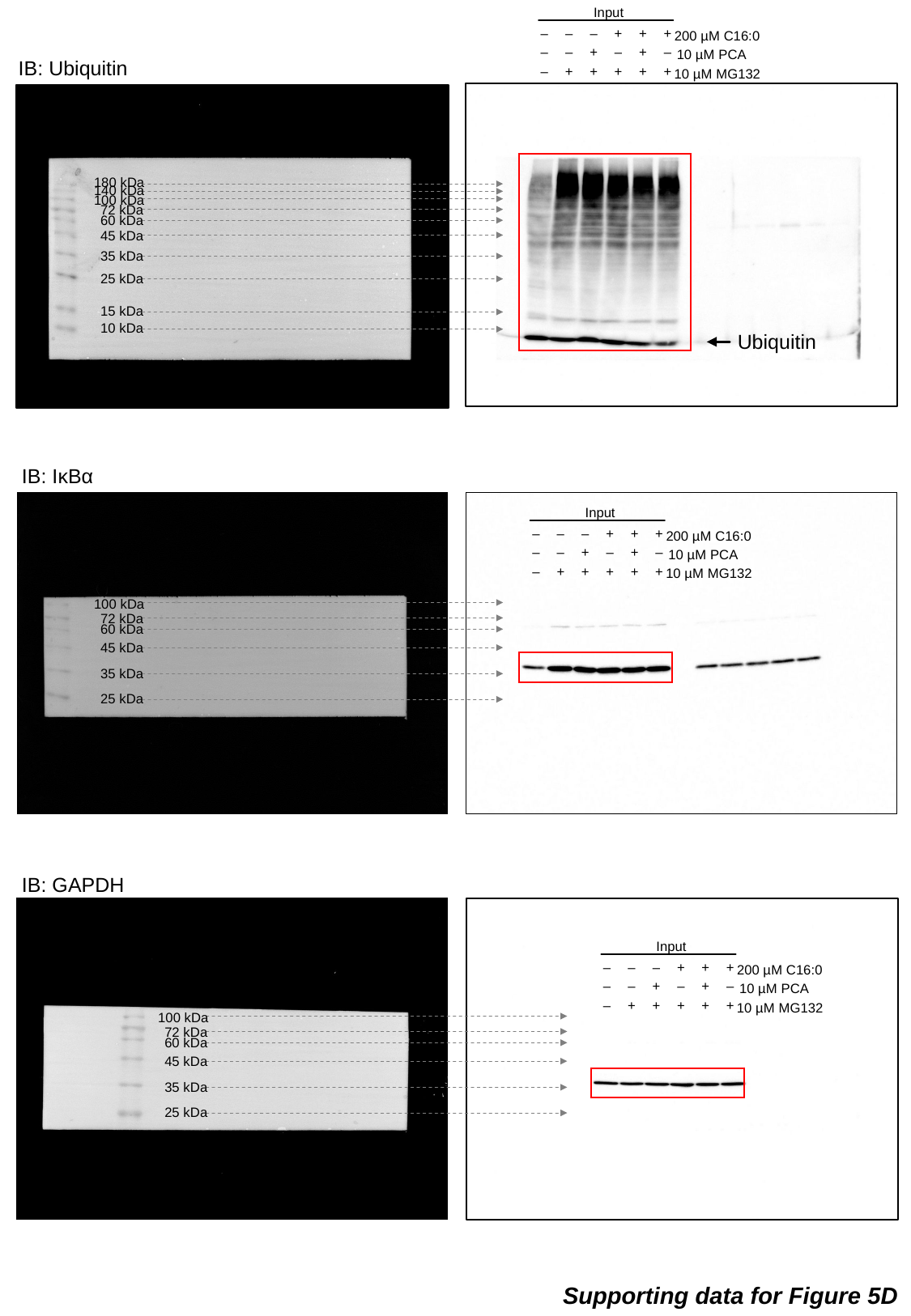

Input
–
–
–
+
+
+
–
–
+
–
+
–
–
+
+
+
+
+
200 µM C16:0
10 µM PCA
10 µM MG132
IB: Ubiquitin
180 kDa
140 kDa
100 kDa
72 kDa
60 kDa
45 kDa
35 kDa
25 kDa
15 kDa
10 kDa
Ubiquitin
IB: IκBα
Input
–
–
–
+
+
+
–
–
+
–
+
–
–
+
+
+
+
+
200 µM C16:0
10 µM PCA
10 µM MG132
100 kDa
72 kDa
60 kDa
45 kDa
35 kDa
25 kDa
IB: GAPDH
Input
–
–
–
+
+
+
–
–
+
–
+
–
–
+
+
+
+
+
200 µM C16:0
10 µM PCA
10 µM MG132
100 kDa
72 kDa
60 kDa
45 kDa
35 kDa
25 kDa
Supporting data for Figure 5D

## Slide 12
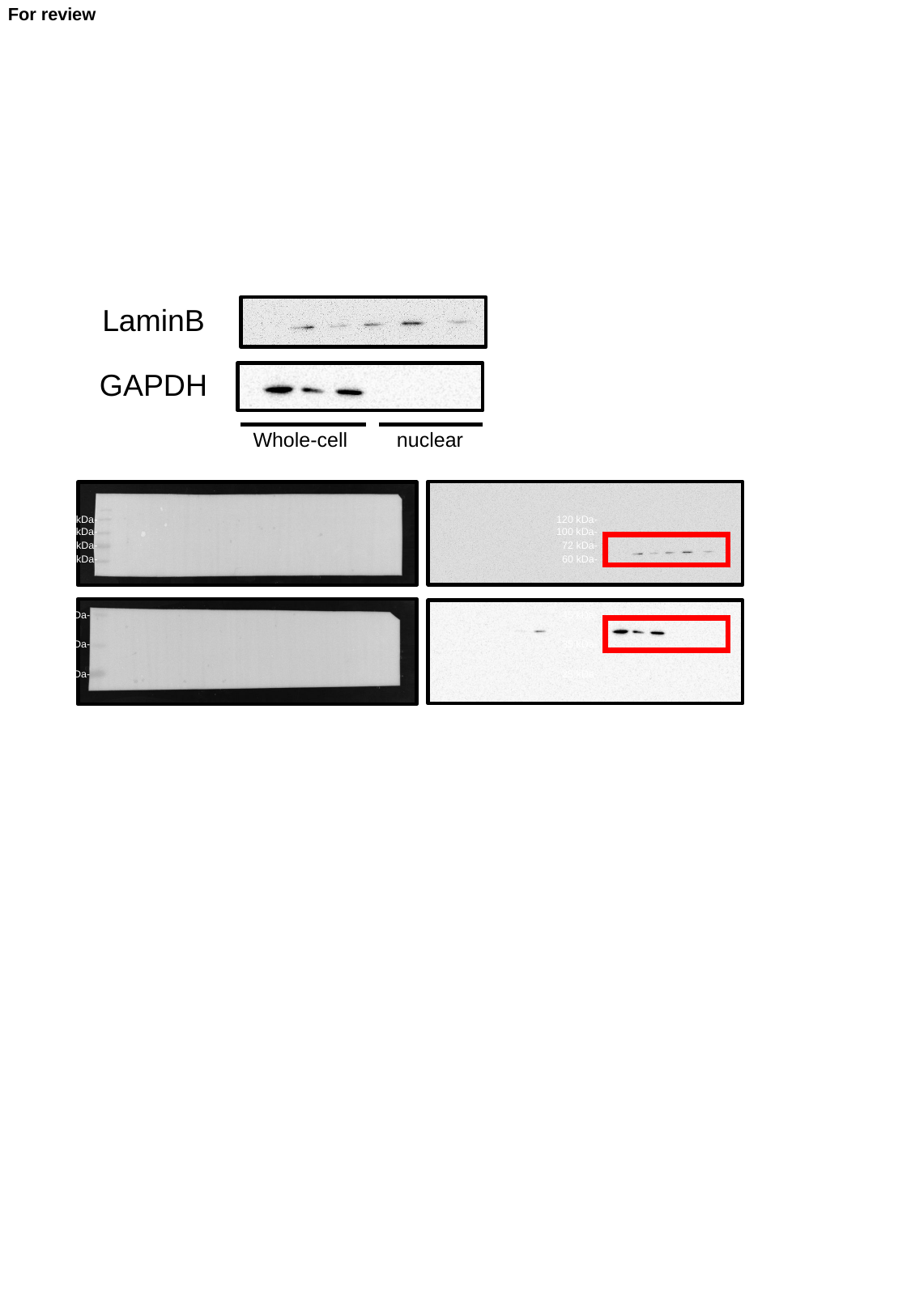

For review
LaminB
GAPDH
Whole-cell
nuclear
120 kDa-
120 kDa-
100 kDa-
100 kDa-
72 kDa-
72 kDa-
60 kDa-
60 kDa-
45 kDa-
45 kDa-
35 kDa-
35 kDa-
25 kDa-
25 kDa-
