# Supplementary material for: Protocatechuic acid prevents obesity caused by long-chain saturated fatty acid-induced inflammation in mouse microglia via inhibition of the NF-κB pathway
Source: PLoS One. 2026 Jun 1;21(6):e0347055. doi: 10.1371/journal.pone.0347055 (PMC13225654; doi:10.1371/journal.pone.0347055)
Supplement: S2 File — Unprocessed raw data and corresponding membrane images of Western blotting experiments used for protein expression quantification and statistical analyses in the main figures. (PPTX) [file pone.0347055.s008.pptx]

## Slide 1
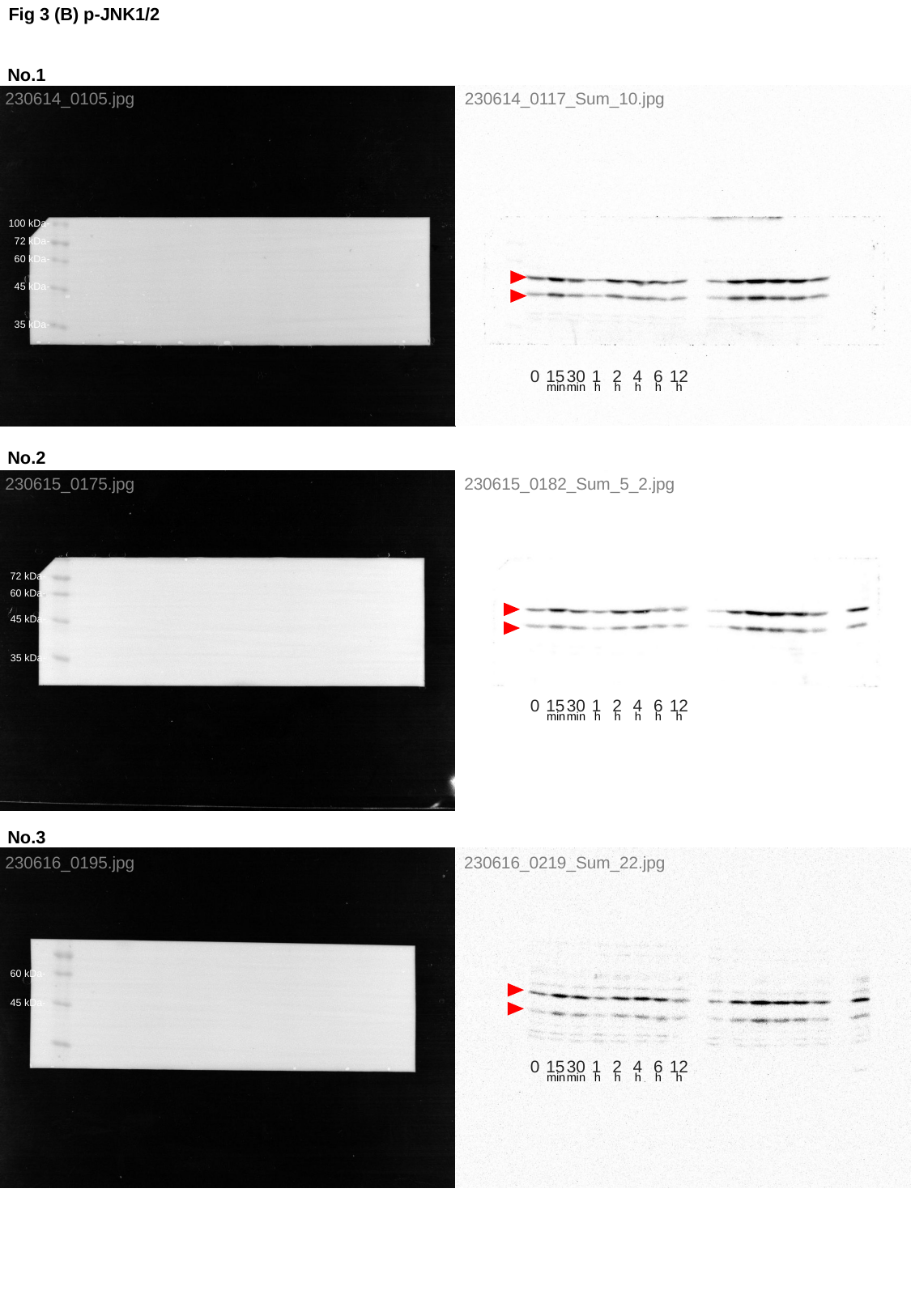

Fig 3 (B) p-JNK1/2
No.1
230614_0105.jpg
230614_0117_Sum_10.jpg
100 kDa-
100 kDa-
72 kDa-
72 kDa-
60 kDa-
60 kDa-
45 kDa-
45 kDa-
35 kDa-
35 kDa-
0
15
30
1
2
4
6
12
min
min
h
h
h
h
h
No.2
230615_0175.jpg
230615_0182_Sum_5_2.jpg
72 kDa-
72 kDa-
60 kDa-
60 kDa-
45 kDa-
45 kDa-
35 kDa-
35 kDa-
0
15
30
1
2
4
6
12
min
min
h
h
h
h
h
No.3
230616_0195.jpg
230616_0219_Sum_22.jpg
60 kDa-
62 kDa-
45 kDa-
45 kDa-
0
15
30
1
2
4
6
12
min
min
h
h
h
h
h

## Slide 2
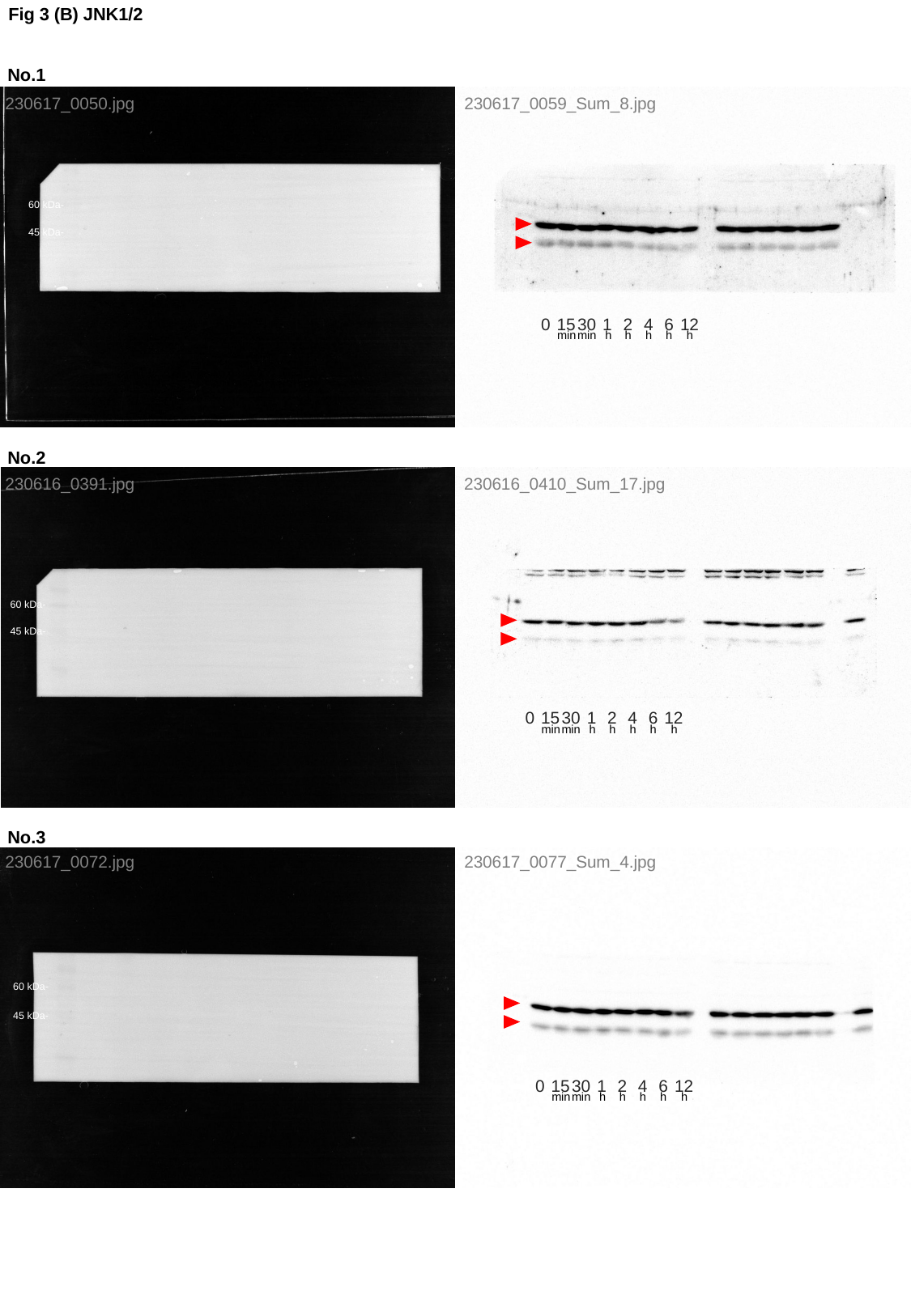

Fig 3 (B) JNK1/2
No.1
230617_0050.jpg
230617_0059_Sum_8.jpg
60 kDa-
60 kDa-
45 kDa-
45 kDa-
0
15
30
1
2
4
6
12
min
min
h
h
h
h
h
No.2
230616_0391.jpg
230616_0410_Sum_17.jpg
60 kDa-
62 kDa-
45 kDa-
45 kDa-
0
15
30
1
2
4
6
12
min
min
h
h
h
h
h
No.3
230617_0072.jpg
230617_0077_Sum_4.jpg
60 kDa-
60 kDa-
45 kDa-
45 kDa-
0
15
30
1
2
4
6
12
min
min
h
h
h
h
h

## Slide 3
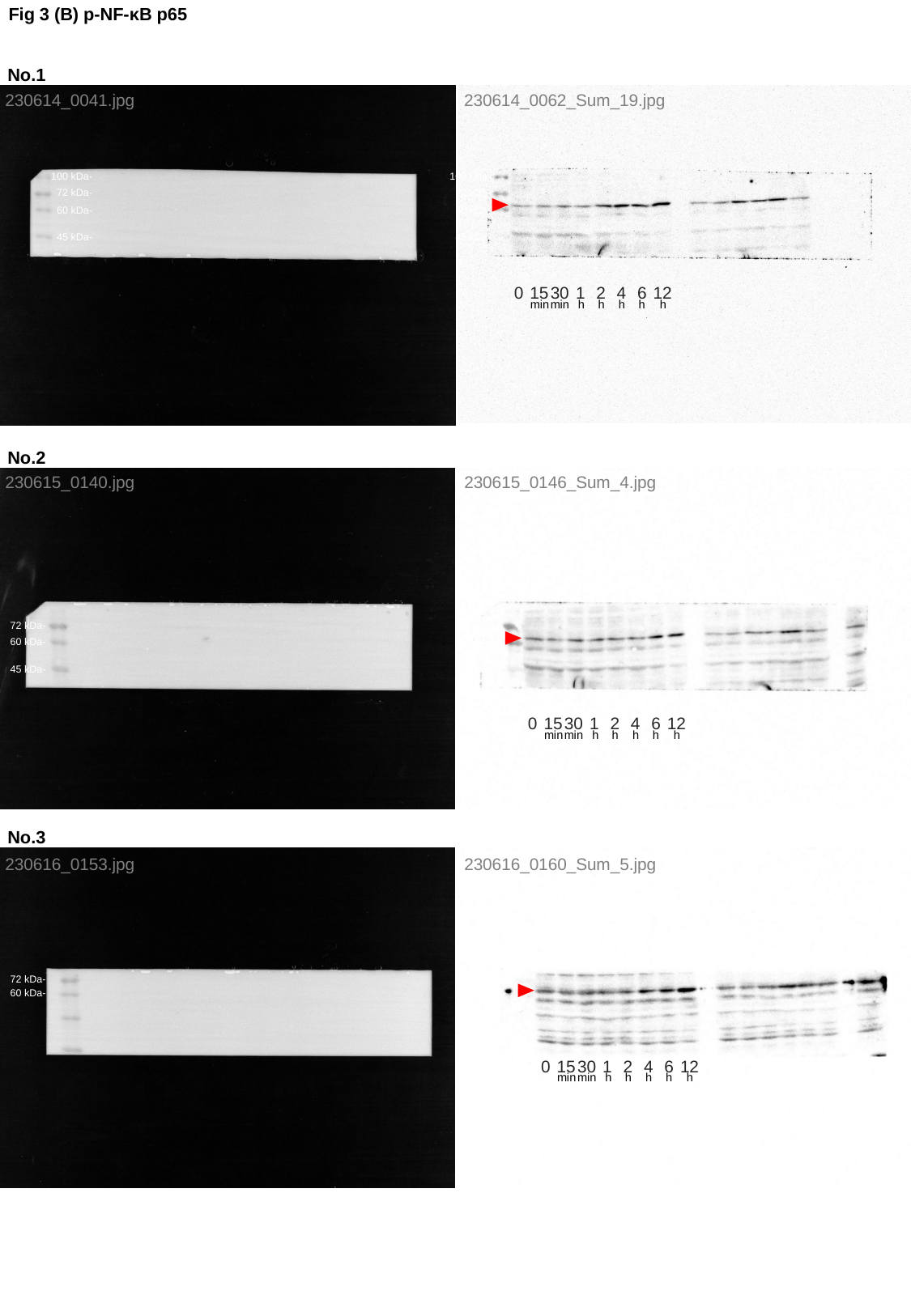

Fig 3 (B) p-NF-κB p65
No.1
230614_0041.jpg
230614_0062_Sum_19.jpg
100 kDa-
100 kDa-
72 kDa-
72 kDa-
60 kDa-
60 kDa-
45 kDa-
45 kDa-
0
15
30
1
2
4
6
12
min
min
h
h
h
h
h
No.2
230615_0140.jpg
230615_0146_Sum_4.jpg
72 kDa-
72 kDa-
60 kDa-
60 kDa-
45 kDa-
45 kDa-
0
15
30
1
2
4
6
12
min
min
h
h
h
h
h
No.3
230616_0153.jpg
230616_0160_Sum_5.jpg
72 kDa-
72 kDa-
60 kDa-
60 kDa-
0
15
30
1
2
4
6
12
min
min
h
h
h
h
h

## Slide 4
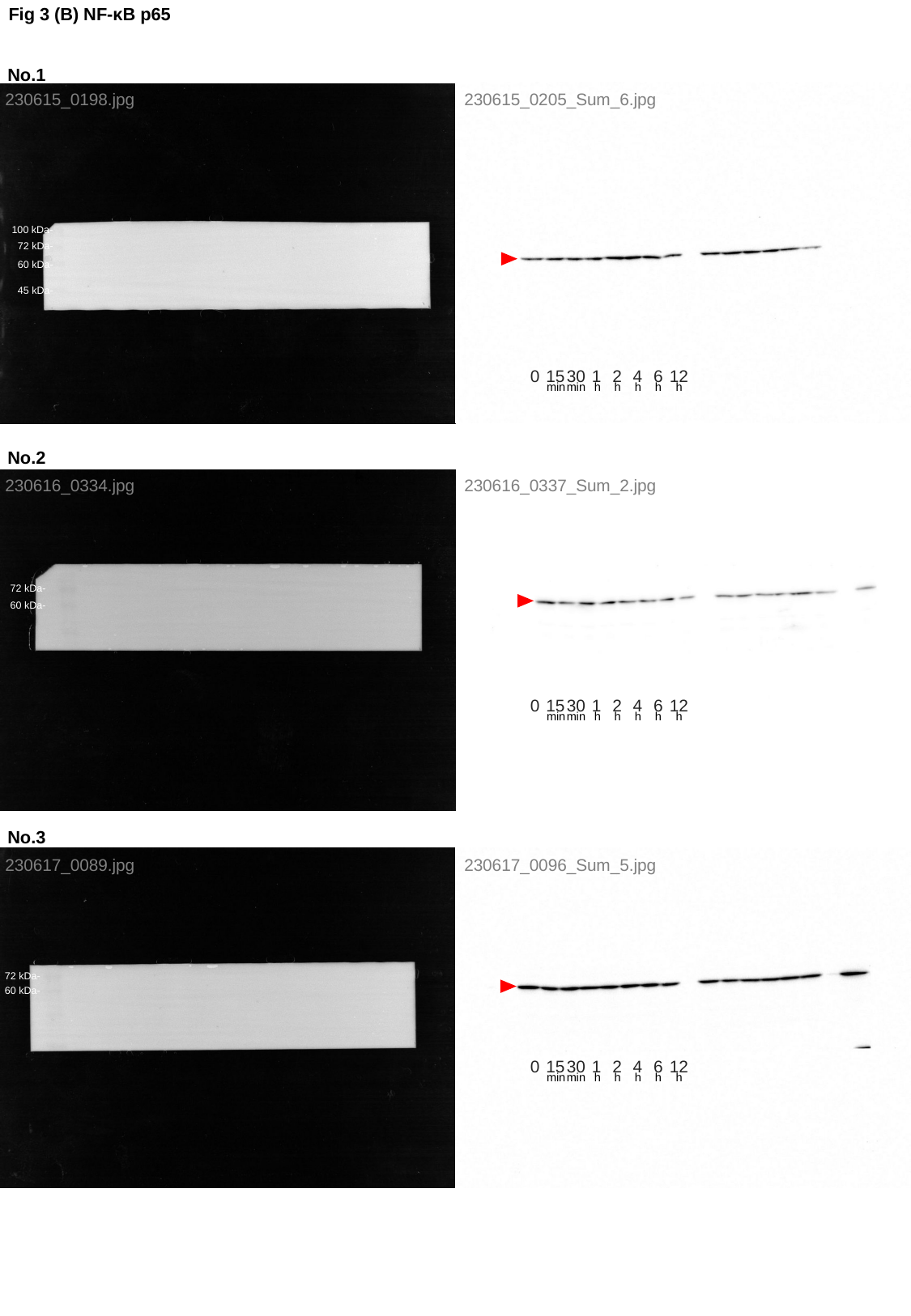

Fig 3 (B) NF-κB p65
No.1
230615_0198.jpg
230615_0205_Sum_6.jpg
100 kDa-
100 kDa-
72 kDa-
72 kDa-
60 kDa-
60 kDa-
45 kDa-
45 kDa-
0
15
30
1
2
4
6
12
min
min
h
h
h
h
h
No.2
230616_0334.jpg
230616_0337_Sum_2.jpg
72 kDa-
75 kDa-
60 kDa-
62 kDa-
0
15
30
1
2
4
6
12
min
min
h
h
h
h
h
No.3
230617_0089.jpg
230617_0096_Sum_5.jpg
72 kDa-
72 kDa-
60 kDa-
60 kDa-
0
15
30
1
2
4
6
12
min
min
h
h
h
h
h

## Slide 5
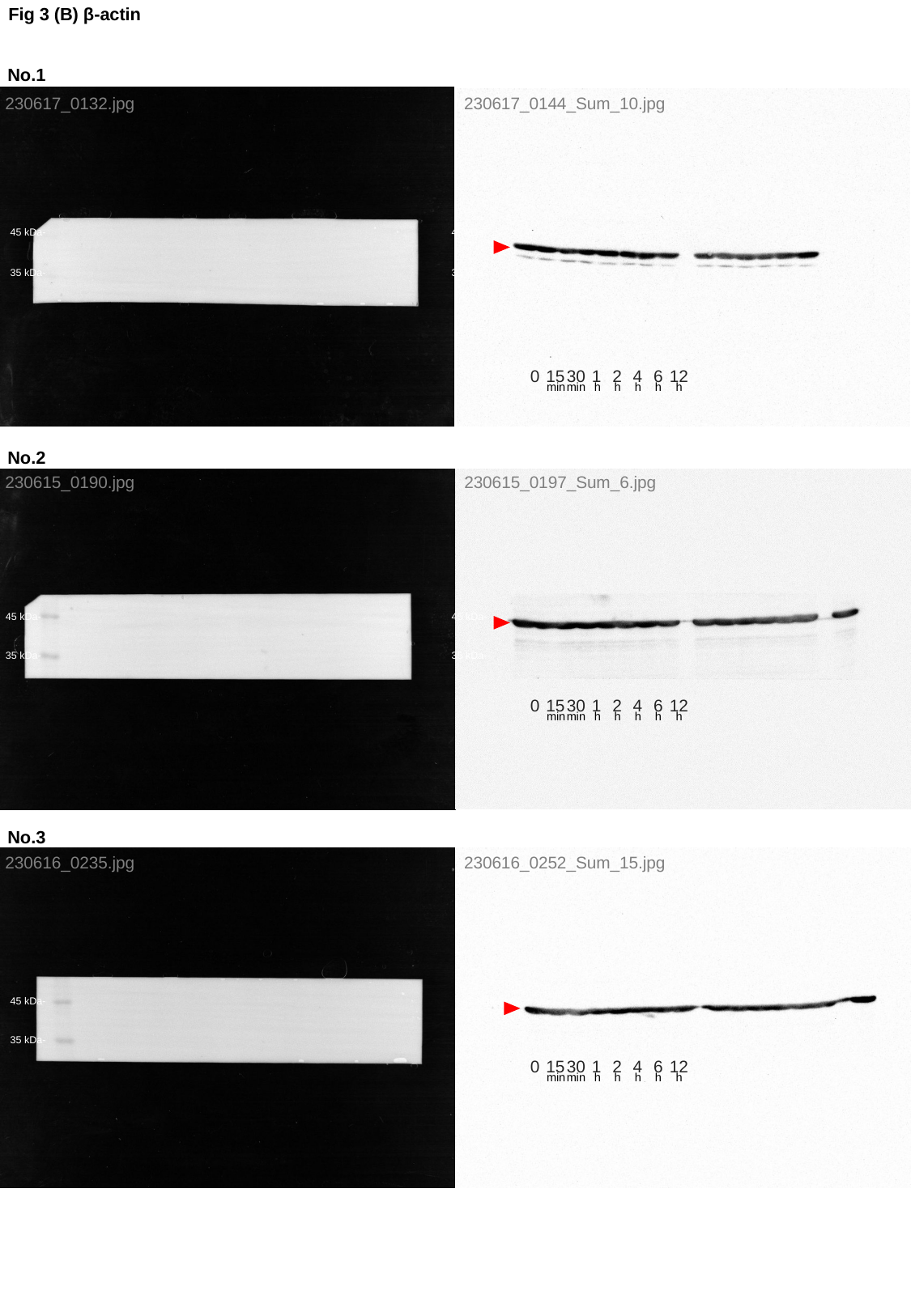

Fig 3 (B) β-actin
No.1
230617_0132.jpg
230617_0144_Sum_10.jpg
45 kDa-
45 kDa-
35 kDa-
35 kDa-
0
15
30
1
2
4
6
12
min
min
h
h
h
h
h
No.2
230615_0190.jpg
230615_0197_Sum_6.jpg
45 kDa-
45 kDa-
35 kDa-
35 kDa-
0
15
30
1
2
4
6
12
min
min
h
h
h
h
h
No.3
230616_0235.jpg
230616_0252_Sum_15.jpg
45 kDa-
45 kDa-
35 kDa-
35 kDa-
0
15
30
1
2
4
6
12
min
min
h
h
h
h
h

## Slide 6
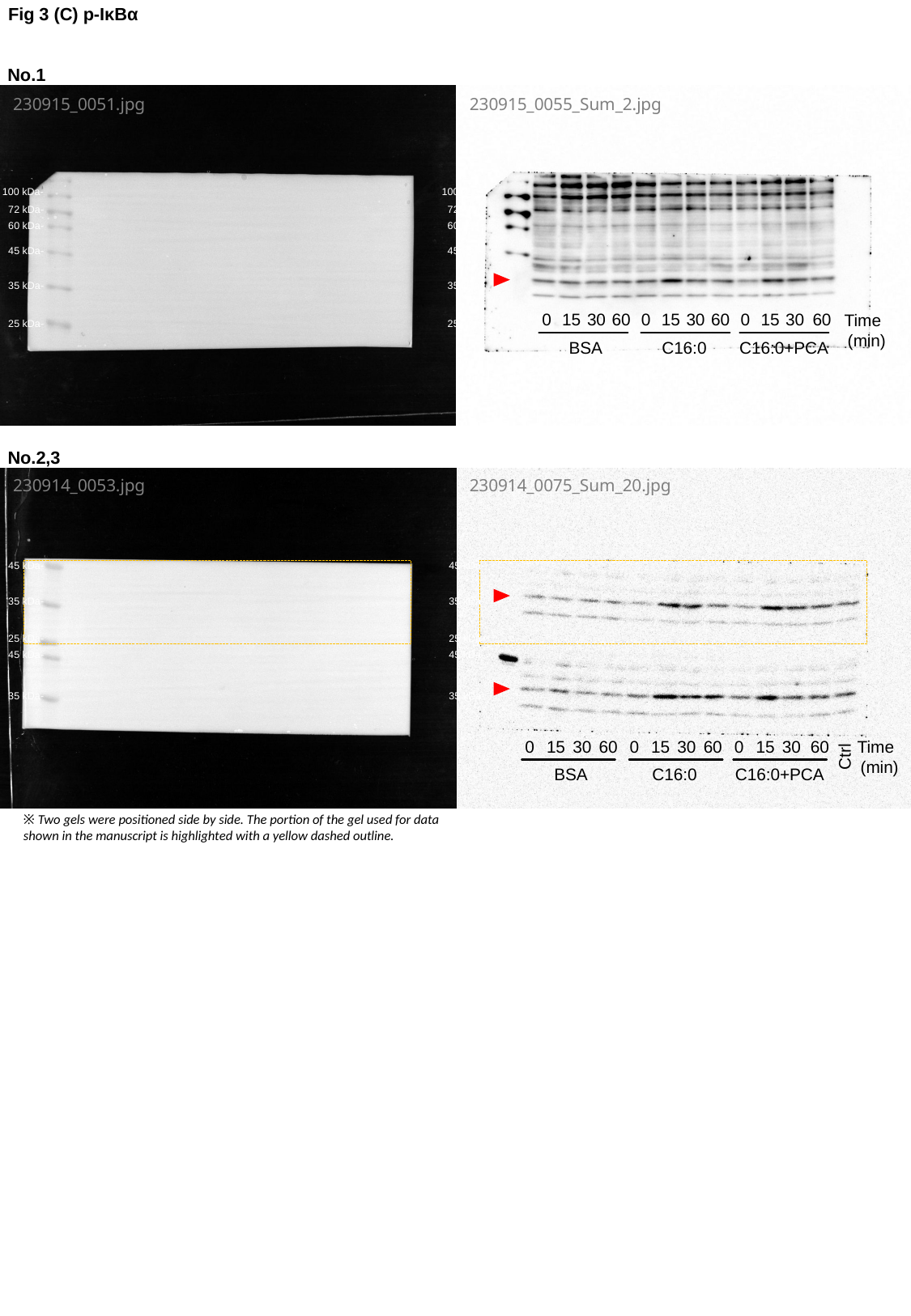

Fig 3 (C) p-IκBα
No.1
230915_0055_Sum_2.jpg
230915_0051.jpg
100 kDa-
100 kDa-
72 kDa-
72 kDa-
60 kDa-
60 kDa-
45 kDa-
45 kDa-
35 kDa-
35 kDa-
Time
 (min)
0
15
30
60
0
15
30
60
0
15
30
60
BSA
C16:0
C16:0+PCA
25 kDa-
25 kDa-
No.2,3
230914_0075_Sum_20.jpg
230914_0053.jpg
45 kDa-
45 kDa-
35 kDa-
35 kDa-
25 kDa-
25 kDa-
45 kDa-
45 kDa-
35 kDa-
35 kDa-
Time
 (min)
0
15
30
60
0
15
30
60
0
15
30
60
BSA
C16:0
C16:0+PCA
Ctrl
※ Two gels were positioned side by side. The portion of the gel used for data shown in the manuscript is highlighted with a yellow dashed outline.

## Slide 7
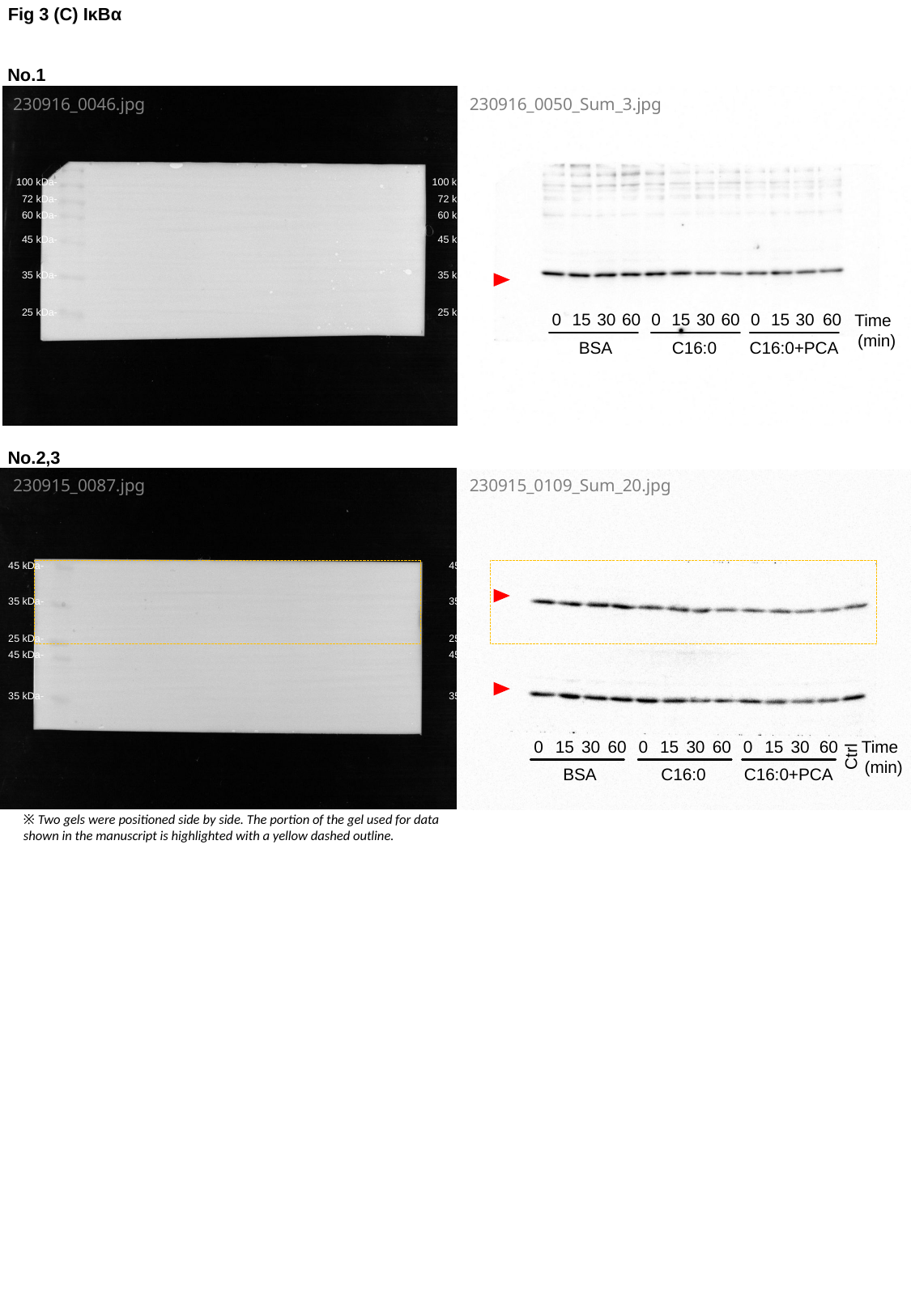

Fig 3 (C) IκBα
No.1
230916_0050_Sum_3.jpg
230916_0046.jpg
100 kDa-
100 kDa-
72 kDa-
72 kDa-
60 kDa-
60 kDa-
45 kDa-
45 kDa-
35 kDa-
35 kDa-
25 kDa-
25 kDa-
Time
 (min)
0
15
30
60
0
15
30
60
0
15
30
60
BSA
C16:0
C16:0+PCA
No.2,3
230915_0109_Sum_20.jpg
230915_0087.jpg
45 kDa-
45 kDa-
35 kDa-
35 kDa-
25 kDa-
25 kDa-
45 kDa-
45 kDa-
35 kDa-
35 kDa-
Time
 (min)
0
15
30
60
0
15
30
60
0
15
30
60
Ctrl
BSA
C16:0
C16:0+PCA
※ Two gels were positioned side by side. The portion of the gel used for data shown in the manuscript is highlighted with a yellow dashed outline.

## Slide 8
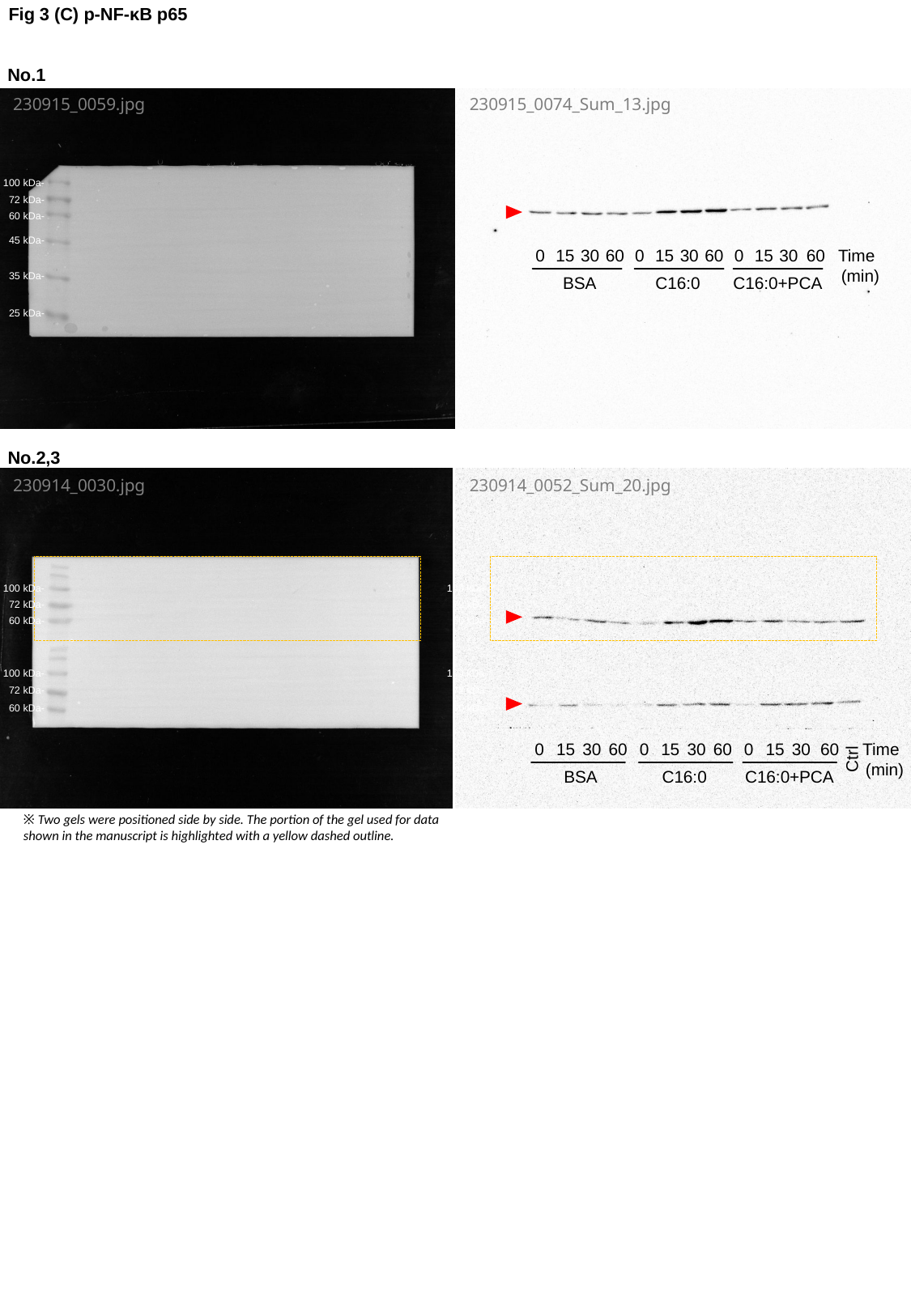

Fig 3 (C) p-NF-κB p65
No.1
230915_0074_Sum_13.jpg
230915_0059.jpg
100 kDa-
72 kDa-
60 kDa-
45 kDa-
Time
 (min)
0
15
30
60
0
15
30
60
0
15
30
60
BSA
C16:0
C16:0+PCA
35 kDa-
25 kDa-
No.2,3
230914_0052_Sum_20.jpg
230914_0030.jpg
100 kDa-
100 kDa-
72 kDa-
72 kDa-
60 kDa-
60 kDa-
100 kDa-
100 kDa-
72 kDa-
72 kDa-
60 kDa-
60 kDa-
Time
 (min)
0
15
30
60
0
15
30
60
0
15
30
60
Ctrl
BSA
C16:0
C16:0+PCA
※ Two gels were positioned side by side. The portion of the gel used for data shown in the manuscript is highlighted with a yellow dashed outline.

## Slide 9
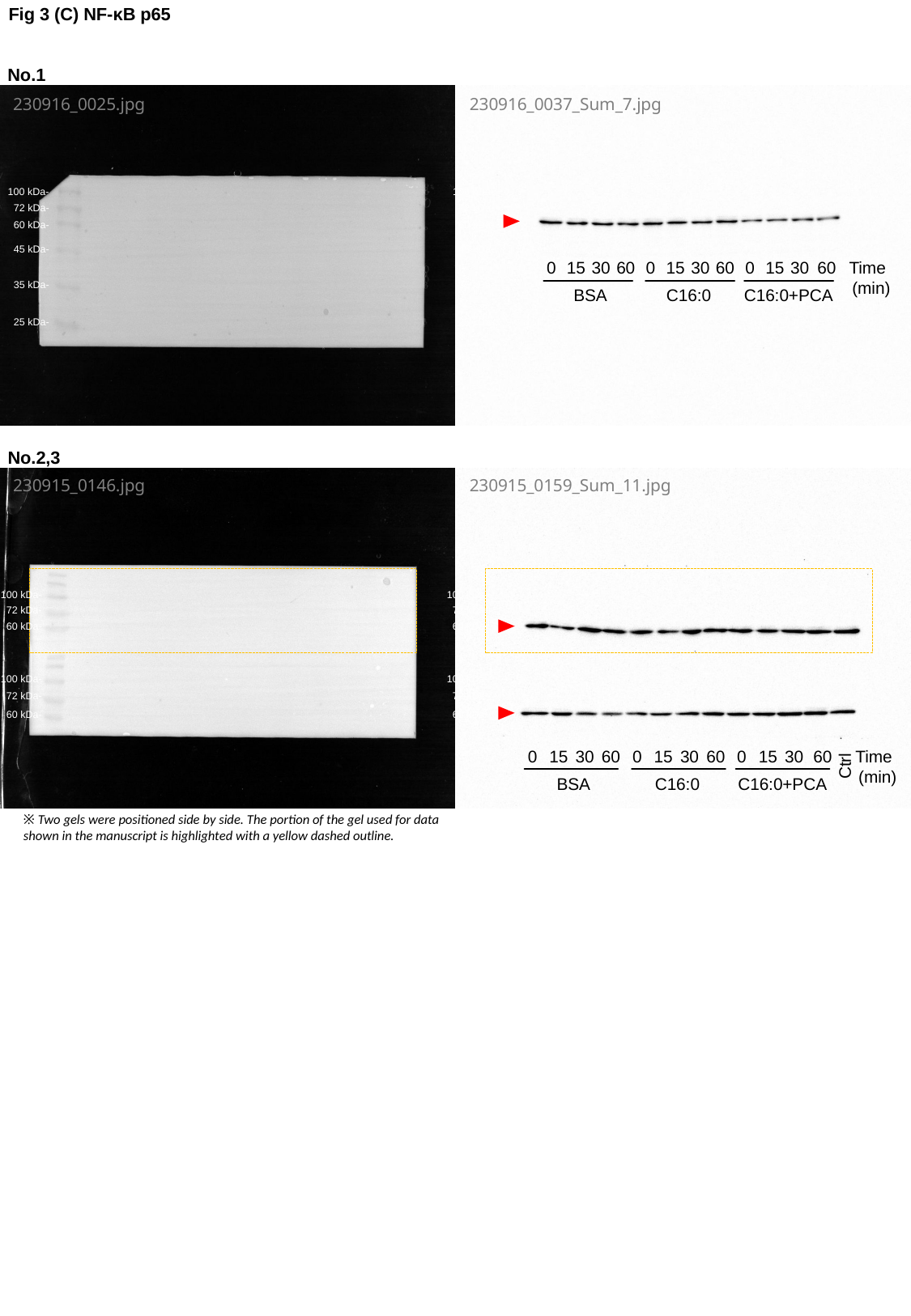

Fig 3 (C) NF-κB p65
No.1
230916_0037_Sum_7.jpg
230916_0025.jpg
100 kDa-
100 kDa-
72 kDa-
72 kDa-
60 kDa-
60 kDa-
45 kDa-
45 kDa-
Time
 (min)
0
15
30
60
0
15
30
60
0
15
30
60
BSA
C16:0
C16:0+PCA
35 kDa-
35 kDa-
25 kDa-
25 kDa-
No.2,3
230915_0159_Sum_11.jpg
230915_0146.jpg
100 kDa-
100 kDa-
72 kDa-
72 kDa-
60 kDa-
60 kDa-
100 kDa-
100 kDa-
72 kDa-
72 kDa-
60 kDa-
60 kDa-
Time
 (min)
0
15
30
60
0
15
30
60
0
15
30
60
Ctrl
BSA
C16:0
C16:0+PCA
※ Two gels were positioned side by side. The portion of the gel used for data shown in the manuscript is highlighted with a yellow dashed outline.

## Slide 10
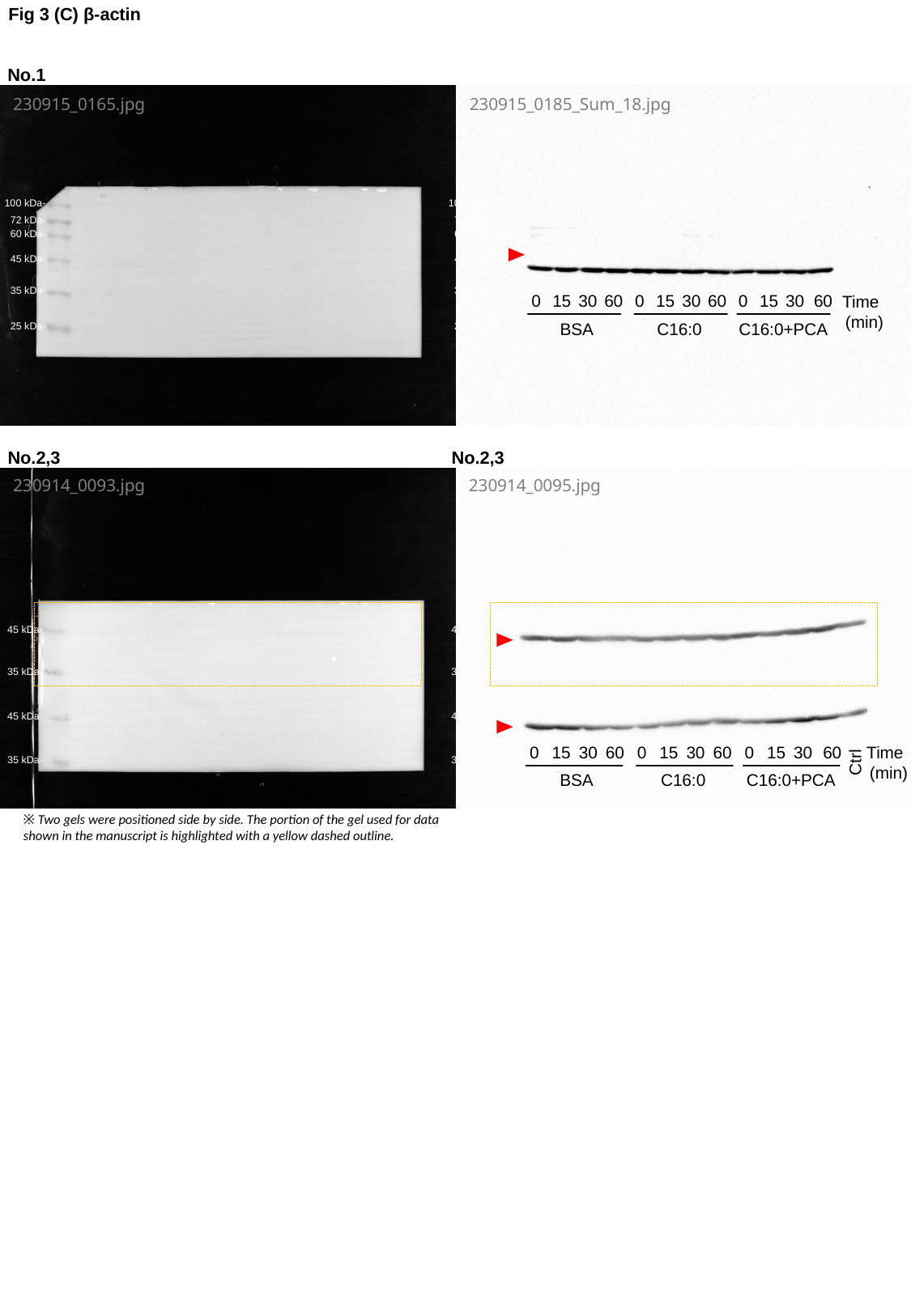

Fig 3 (C) β-actin
No.1
230915_0185_Sum_18.jpg
230915_0165.jpg
100 kDa-
100 kDa-
72 kDa-
72 kDa-
60 kDa-
60 kDa-
45 kDa-
45 kDa-
35 kDa-
35 kDa-
Time
 (min)
0
15
30
60
0
15
30
60
0
15
30
60
BSA
C16:0
C16:0+PCA
25 kDa-
25 kDa-
No.2,3
No.2,3
230914_0095.jpg
230914_0093.jpg
45 kDa-
45 kDa-
35 kDa-
35 kDa-
45 kDa-
45 kDa-
Time
 (min)
0
15
30
60
0
15
30
60
0
15
30
60
Ctrl
BSA
C16:0
C16:0+PCA
35 kDa-
35 kDa-
※ Two gels were positioned side by side. The portion of the gel used for data shown in the manuscript is highlighted with a yellow dashed outline.

## Slide 11
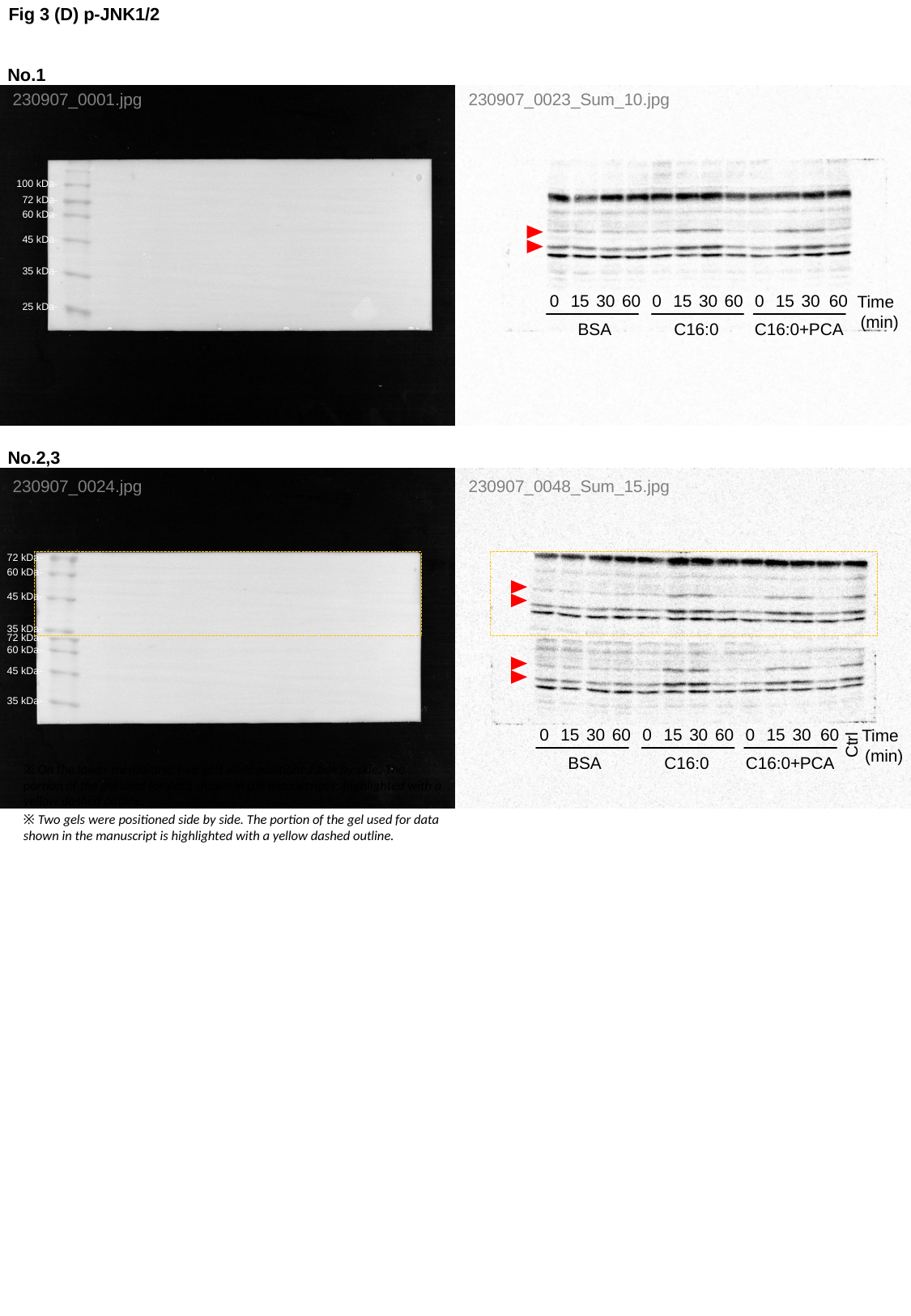

Fig 3 (D) p-JNK1/2
No.1
230907_0001.jpg
230907_0023_Sum_10.jpg
100 kDa-
100 kDa-
72 kDa-
72 kDa-
60 kDa-
60 kDa-
45 kDa-
45 kDa-
35 kDa-
35 kDa-
Time
 (min)
0
15
30
60
0
15
30
60
0
15
30
60
BSA
C16:0
C16:0+PCA
25 kDa-
25 kDa-
No.2,3
230907_0024.jpg
230907_0048_Sum_15.jpg
72 kDa-
72 kDa-
60 kDa-
60 kDa-
45 kDa-
45 kDa-
35 kDa-
35 kDa-
72 kDa-
72 kDa-
60 kDa-
60 kDa-
45 kDa-
45 kDa-
35 kDa-
35 kDa-
Time
 (min)
0
15
30
60
0
15
30
60
0
15
30
60
Ctrl
BSA
C16:0
C16:0+PCA
※ On the lower membrane, two gels were positioned side by side. The portion of the gel used for data shown in the manuscript is highlighted with a yellow dashed outline.
※ Two gels were positioned side by side. The portion of the gel used for data shown in the manuscript is highlighted with a yellow dashed outline.

## Slide 12
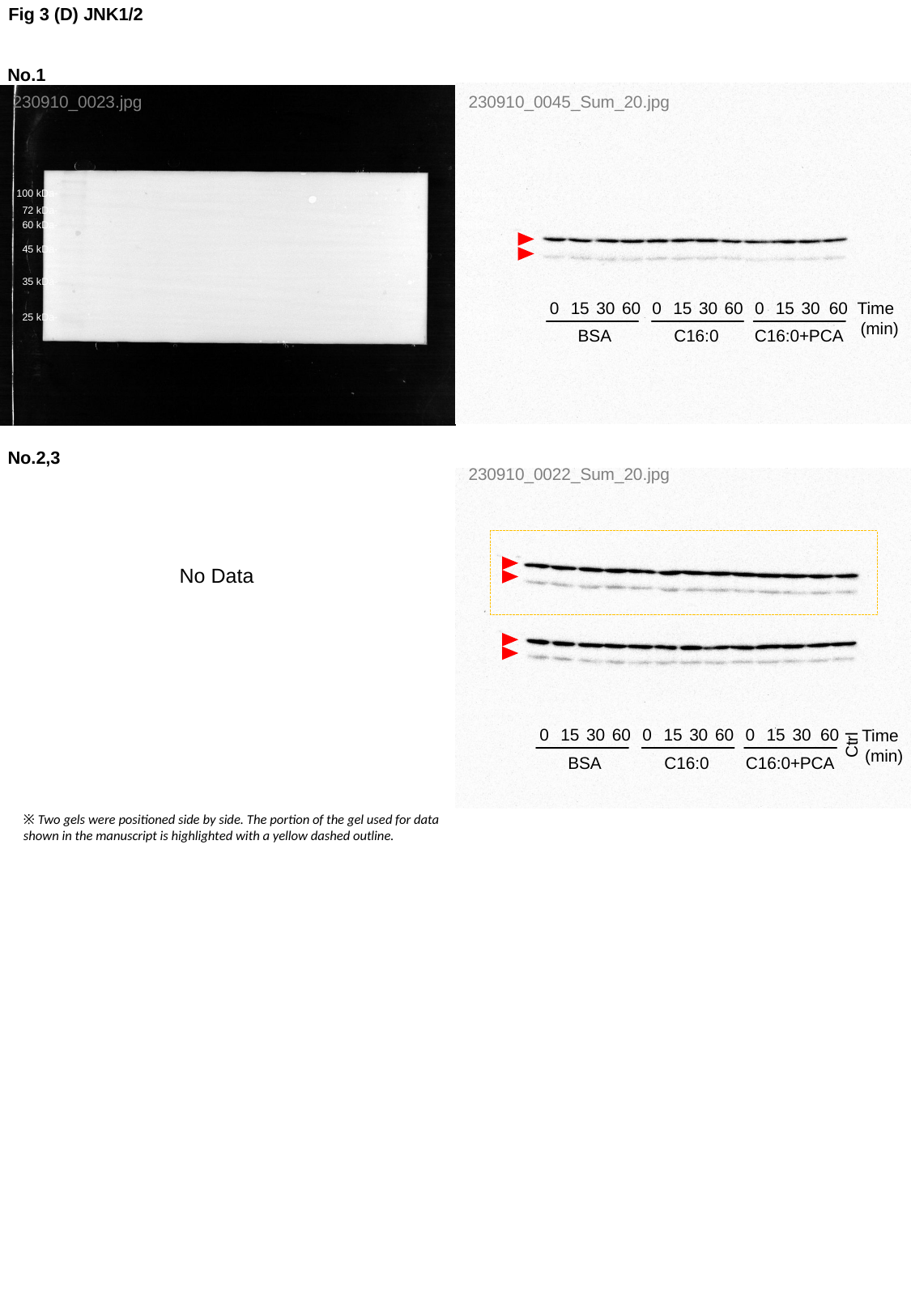

Fig 3 (D) JNK1/2
No.1
230910_0023.jpg
230910_0045_Sum_20.jpg
100 kDa-
100 kDa-
72 kDa-
72 kDa-
60 kDa-
60 kDa-
45 kDa-
45 kDa-
35 kDa-
35 kDa-
Time
 (min)
0
15
30
60
0
15
30
60
0
15
30
60
BSA
C16:0
C16:0+PCA
25 kDa-
25 kDa-
No.2,3
230910_0022_Sum_20.jpg
No Data
Time
 (min)
0
15
30
60
0
15
30
60
0
15
30
60
Ctrl
BSA
C16:0
C16:0+PCA
※ Two gels were positioned side by side. The portion of the gel used for data shown in the manuscript is highlighted with a yellow dashed outline.

## Slide 13
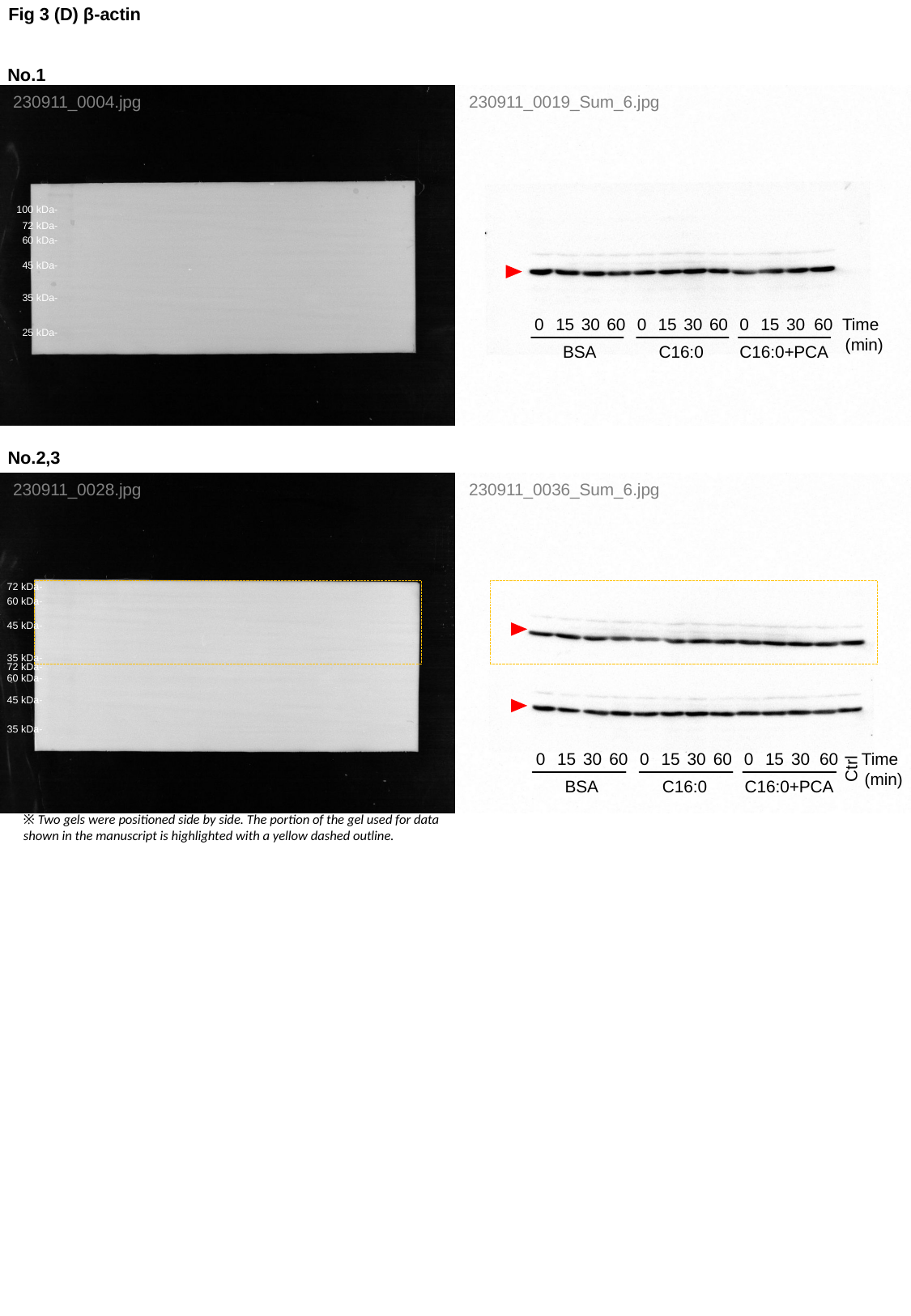

Fig 3 (D) β-actin
No.1
230911_0004.jpg
230911_0019_Sum_6.jpg
100 kDa-
100 kDa-
72 kDa-
72 kDa-
60 kDa-
60 kDa-
45 kDa-
45 kDa-
35 kDa-
35 kDa-
Time
 (min)
0
15
30
60
0
15
30
60
0
15
30
60
BSA
C16:0
C16:0+PCA
25 kDa-
25 kDa-
No.2,3
230911_0028.jpg
230911_0036_Sum_6.jpg
72 kDa-
72 kDa-
60 kDa-
60 kDa-
45 kDa-
45 kDa-
35 kDa-
35 kDa-
72 kDa-
72 kDa-
60 kDa-
60 kDa-
45 kDa-
45 kDa-
35 kDa-
35 kDa-
Time
 (min)
0
15
30
60
0
15
30
60
0
15
30
60
Ctrl
BSA
C16:0
C16:0+PCA
※ Two gels were positioned side by side. The portion of the gel used for data shown in the manuscript is highlighted with a yellow dashed outline.

## Slide 14
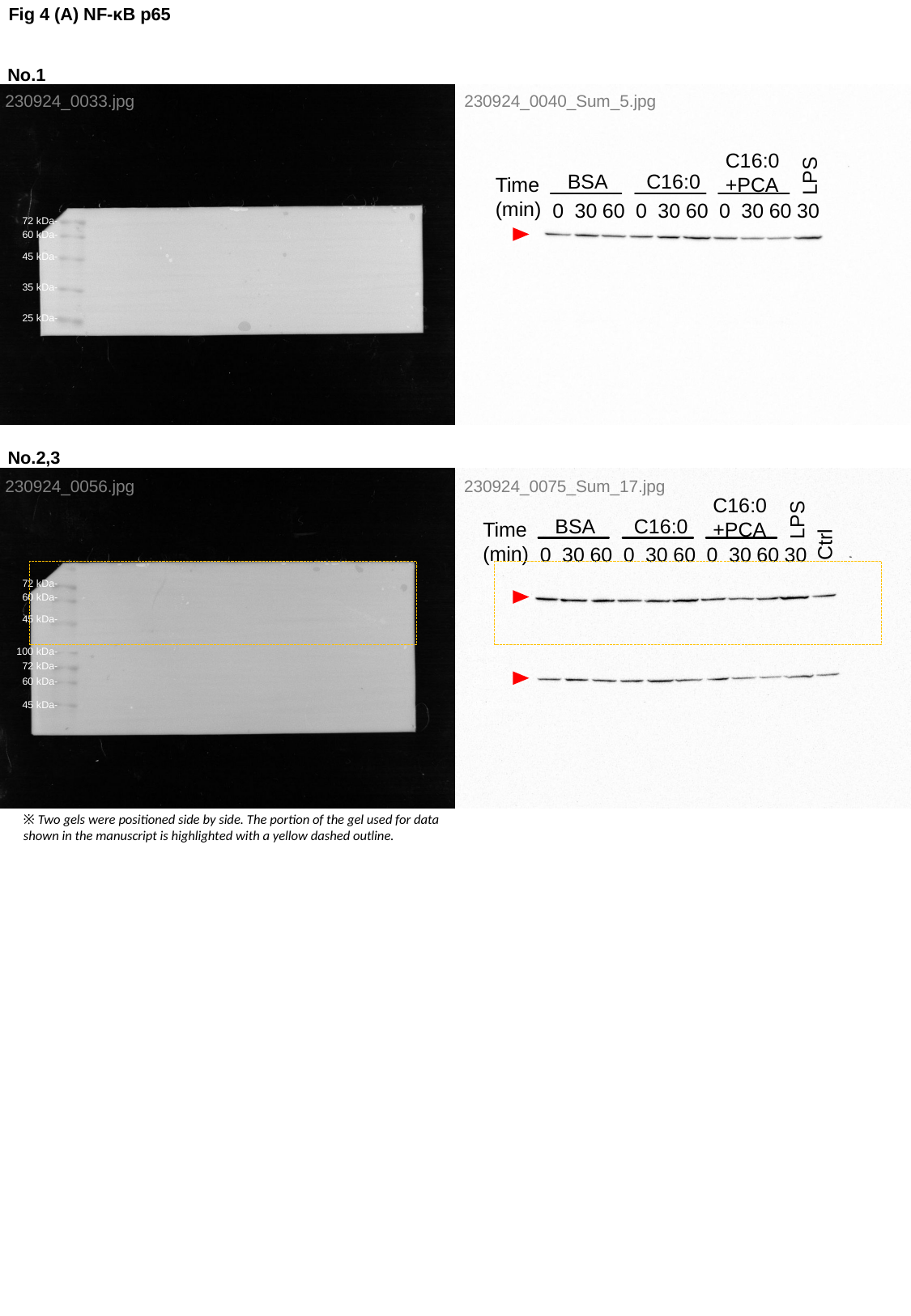

Fig 4 (A) NF-κB p65
No.1
230924_0033.jpg
230924_0040_Sum_5.jpg
C16:0
+PCA
LPS
BSA
C16:0
Time
(min)
0
30
60
0
30
60
0
30
60
30
72 kDa-
72 kDa-
60 kDa-
60 kDa-
45 kDa-
45 kDa-
35 kDa-
35 kDa-
25 kDa-
25 kDa-
No.2,3
230924_0056.jpg
230924_0075_Sum_17.jpg
C16:0
+PCA
LPS
BSA
C16:0
Time
(min)
0
30
60
0
30
60
0
30
60
30
Ctrl
72 kDa-
72 kDa-
60 kDa-
60 kDa-
45 kDa-
45 kDa-
100 kDa-
100 kDa-
72 kDa-
72 kDa-
60 kDa-
60 kDa-
45 kDa-
45 kDa-
※ Two gels were positioned side by side. The portion of the gel used for data shown in the manuscript is highlighted with a yellow dashed outline.

## Slide 15
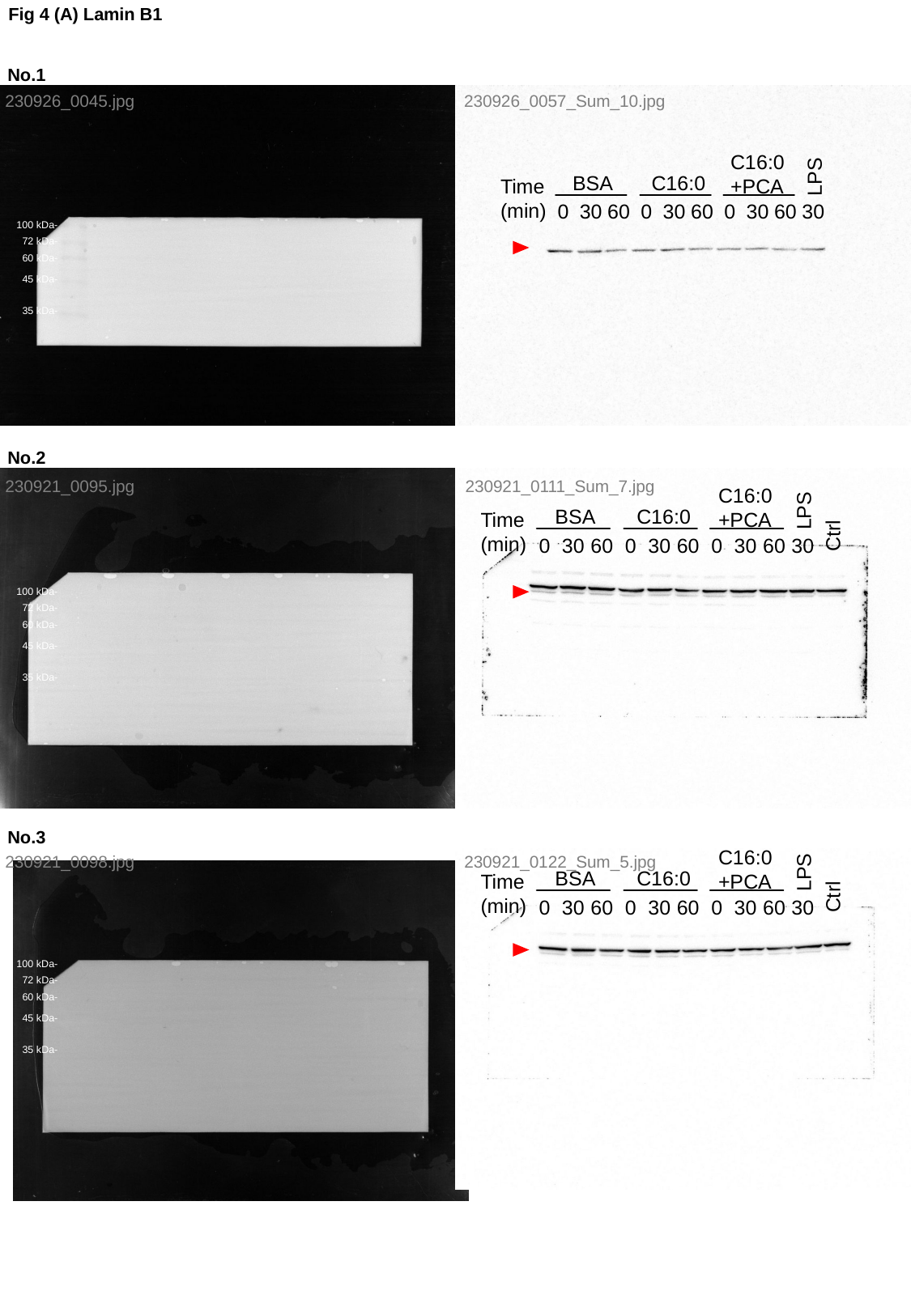

Fig 4 (A) Lamin B1
No.1
230926_0045.jpg
230926_0057_Sum_10.jpg
C16:0
+PCA
LPS
BSA
C16:0
Time
(min)
0
30
60
0
30
60
0
30
60
30
100 kDa-
100 kDa-
72 kDa-
72 kDa-
60 kDa-
60 kDa-
45 kDa-
45 kDa-
35 kDa-
35 kDa-
No.2
230921_0095.jpg
230921_0111_Sum_7.jpg
C16:0
+PCA
LPS
BSA
C16:0
Time
(min)
0
30
60
0
30
60
0
30
60
30
Ctrl
100 kDa-
72 kDa-
100 kDa-
60 kDa-
72 kDa-
45 kDa-
60 kDa-
45 kDa-
35 kDa-
35 kDa-
No.3
C16:0
+PCA
LPS
BSA
C16:0
Time
(min)
0
30
60
0
30
60
0
30
60
30
Ctrl
230921_0098.jpg
230921_0122_Sum_5.jpg
100 kDa-
72 kDa-
60 kDa-
100 kDa-
45 kDa-
72 kDa-
60 kDa-
35 kDa-
45 kDa-
35 kDa-

## Slide 16
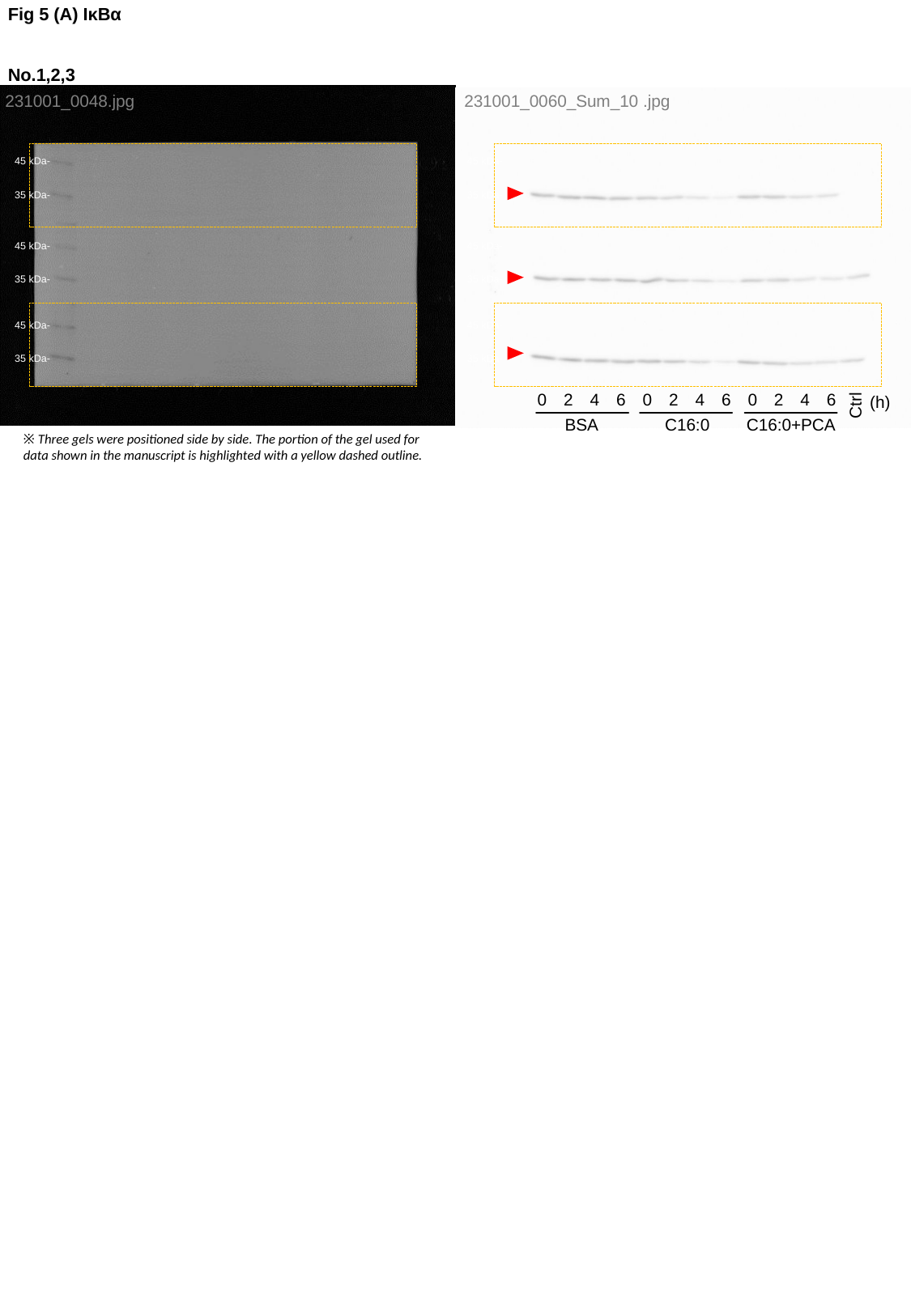

Fig 5 (A) IκBα
No.1,2,3
231001_0048.jpg
231001_0060_Sum_10 .jpg
45 kDa-
45 kDa-
35 kDa-
35 kDa-
45 kDa-
45 kDa-
35 kDa-
35 kDa-
45 kDa-
45 kDa-
35 kDa-
35 kDa-
0
2
4
6
0
2
4
6
0
2
4
6
(h)
Ctrl
BSA
C16:0
C16:0+PCA
※ Three gels were positioned side by side. The portion of the gel used for data shown in the manuscript is highlighted with a yellow dashed outline.

## Slide 17
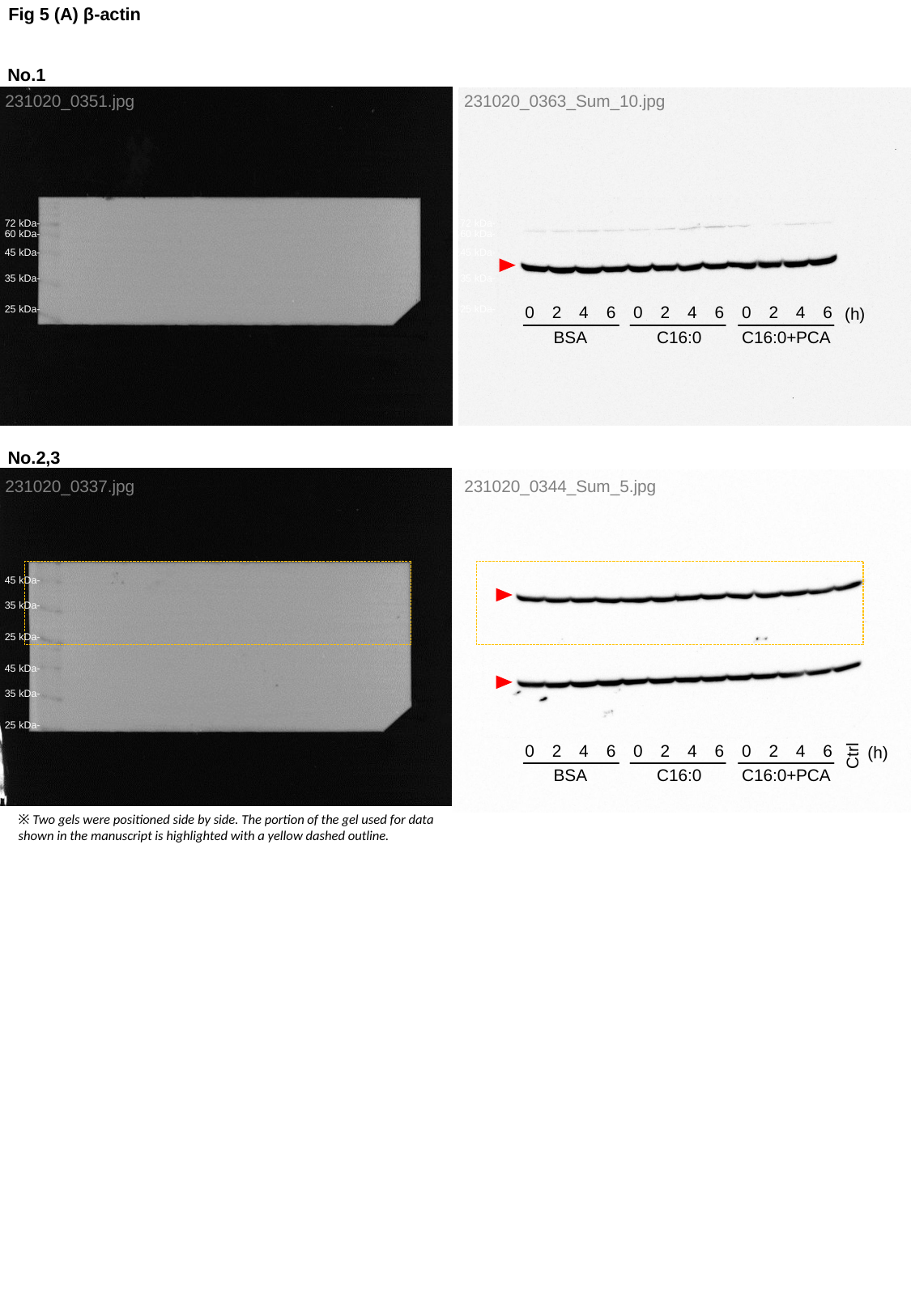

Fig 5 (A) β-actin
No.1
231020_0351.jpg
231020_0363_Sum_10.jpg
72 kDa-
72 kDa-
60 kDa-
60 kDa-
45 kDa-
45 kDa-
35 kDa-
35 kDa-
0
2
4
6
0
2
4
6
0
2
4
6
(h)
BSA
C16:0
C16:0+PCA
25 kDa-
25 kDa-
No.2,3
231020_0337.jpg
231020_0344_Sum_5.jpg
45 kDa-
45 kDa-
35 kDa-
35 kDa-
25 kDa-
25 kDa-
45 kDa-
45 kDa-
35 kDa-
35 kDa-
25 kDa-
25 kDa-
0
2
4
6
0
2
4
6
0
2
4
6
(h)
Ctrl
BSA
C16:0
C16:0+PCA
※ Two gels were positioned side by side. The portion of the gel used for data shown in the manuscript is highlighted with a yellow dashed outline.

## Slide 18
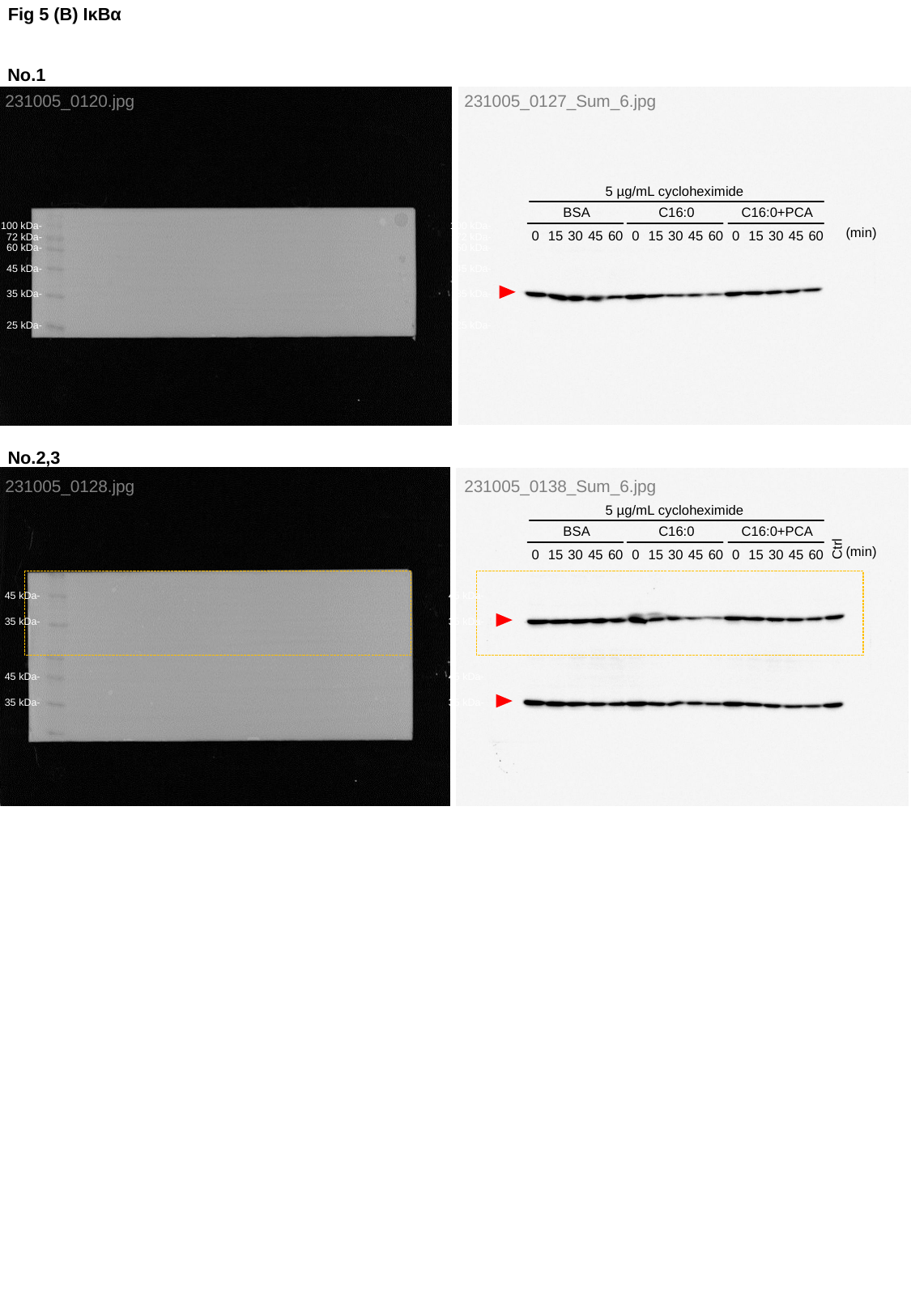

Fig 5 (B) IκBα
No.1
231005_0120.jpg
231005_0127_Sum_6.jpg
5 µg/mL cycloheximide
BSA
C16:0
C16:0+PCA
(min)
0
15
30
45
60
0
15
30
45
60
0
15
30
45
60
100 kDa-
100 kDa-
72 kDa-
72 kDa-
60 kDa-
60 kDa-
45 kDa-
45 kDa-
35 kDa-
35 kDa-
25 kDa-
25 kDa-
No.2,3
231005_0128.jpg
231005_0138_Sum_6.jpg
5 µg/mL cycloheximide
BSA
C16:0
C16:0+PCA
(min)
0
15
30
45
60
0
15
30
45
60
0
15
30
45
60
Ctrl
45 kDa-
45 kDa-
35 kDa-
35 kDa-
45 kDa-
45 kDa-
35 kDa-
35 kDa-

## Slide 19
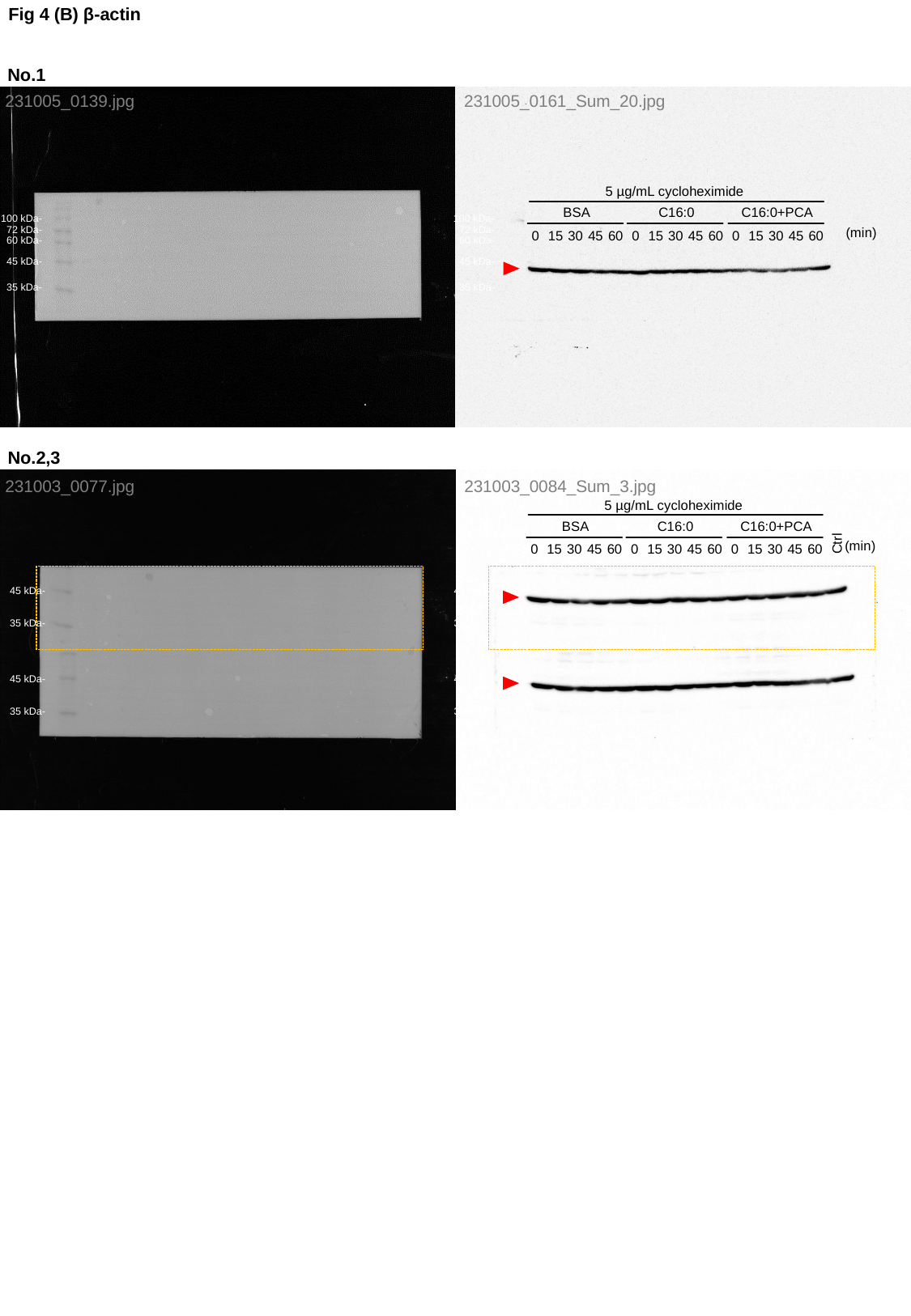

Fig 4 (B) β-actin
No.1
231005_0139.jpg
231005_0161_Sum_20.jpg
5 µg/mL cycloheximide
BSA
C16:0
C16:0+PCA
(min)
0
15
30
45
60
0
15
30
45
60
0
15
30
45
60
100 kDa-
100 kDa-
72 kDa-
72 kDa-
60 kDa-
60 kDa-
45 kDa-
45 kDa-
35 kDa-
35 kDa-
No.2,3
231003_0077.jpg
231003_0084_Sum_3.jpg
5 µg/mL cycloheximide
BSA
C16:0
C16:0+PCA
(min)
0
15
30
45
60
0
15
30
45
60
0
15
30
45
60
Ctrl
45 kDa-
45 kDa-
35 kDa-
35 kDa-
45 kDa-
45 kDa-
35 kDa-
35 kDa-
